# Global DNA hypermethylation pattern and unique gene expression signature in liver cancer from patients with Indigenous American ancestry

**DOI:** 10.18632/oncotarget.27890

**Published:** 2021-03-02

**Authors:** Juan Pablo Cerapio, Agnès Marchio, Luis Cano, Ignacio López, Jean-Jacques Fournié, Béatrice Régnault, Sandro Casavilca-Zambrano, Eloy Ruiz, Anne Dejean, Stéphane Bertani, Pascal Pineau

**Affiliations:** ^1^Sorbonne Université, Institut Pasteur, Unité Organisation Nucléaire et Oncogenèse, INSERM, U 993, Paris, France; ^2^Institut Pasteur, Unité Organisation Nucléaire et Oncogenèse, INSERM, U 993, Paris, France; ^3^Université de Rennes 1, INSERM, CNRS, U 1241 NUMECAN, Rennes, France; ^4^Centre de Recherches en Cancérologie de Toulouse, Université de Toulouse, INSERM, UPS, UMR 1037, CNRS, ERL 5294, Toulouse, France; ^5^Institut Pasteur, Centre d'Innovation et Recherche Technologique, Plateforme de Génotypage des Eucaryotes, Paris, France; ^6^Instituto Nacional de Enfermedades Neoplásicas, Departamento de Patología, Banco de Tejidos Tumorales, Lima, Peru; ^7^Instituto Nacional de Enfermedades Neoplásicas, Departamento de Cirugía en Abdomen, Lima, Peru; ^8^Université de Toulouse, IRD, UPS, UMR 152 PHARMADEV, Toulouse, France; ^*^These authors contributed equally to this work

**Keywords:** hepatitis B virus, indigenous people, integrative genomics, liver cancer

## Abstract

Hepatocellular carcinoma (HCC) usually afflicts individuals in their maturity after a protracted liver disease. Contrasting with this pattern, the age structure of HCC in Andean people displays a bimodal distribution with half of the patients developing HCC in adolescence and early adulthood. To deepen our understanding of the molecular determinants of the disease in this population, we conducted an integrative analysis of gene expression and DNA methylation in HCC developed by 74 Peruvian patients, including 39 adolescents and young adults. While genome-wide hypomethylation is considered as a paradigm in human HCCs, our analysis revealed that Peruvian tumors are associated with a global DNA hypermethylation. Moreover, pathway enrichment analysis of transcriptome data characterized an original combination of signatures. Peruvian HCC forgoes canonical activations of IGF2, Notch, Ras/MAPK, and TGF-β signals to depend instead on Hippo/YAP1, MYC, and Wnt/β-catenin pathways. These signatures delineate a homogeneous subtype of liver tumors at the interface of the proliferative and non-proliferative classes of HCCs. Remarkably, the development of this HCC subtype occurs in patients with one of the four Native American mitochondrial haplogroups A-D. Finally, integrative characterization revealed that Peruvian HCC is apparently controlled by the PRC2 complex that mediates cell reprogramming with massive DNA methylation modulating gene expression and pinpointed retinoid signaling as a potential target for epigenetic therapy.

## INTRODUCTION

Hepatocellular carcinoma (HCC), the main form of primary liver cancer, is one of the leading causes of tumor-related death worldwide [1]. HCC exhibits a high degree of molecular heterogeneity translated to some extent in its clinical presentation [2]. The transcriptome analysis of HCC delineates two major classes (i.e., proliferative and non-proliferative) with differences in pathway activation, phenotypes, and prognosis [3]. The non-proliferative HCC class is correlated to chronic hepatitis C virus infection and alcohol consumption, and it is associated with low circulating α-fetoprotein (AFP) levels, low-grade cancer cells with retention of a certain degree of well-differentiated hepatocyte signaling, and a good prognosis [4]. On the opposite side, the proliferative HCC class is associated with hepatitis B virus (HBV) infection, higher serum AFP levels, more aggressive tumors, and shorter survival [4]. This latter class is further subdivided into hepatocyte- and progenitor-like clusters. Genome-wide hypomethylation is viewed as a transversal hallmark of all HCC subtypes, whereas CpG-rich regions are found focally hypermethylated [5, 6].

This molecular classification of HCCs relies almost exclusively on genomic data from HCC patients of North America, Asia-Pacific, and Europe [2]. Indeed, there is still a scarcity of molecular information available from HCC patients of Africa, Latin America, and Oceania despite distinctive characteristics exhibited by the disease in these regions. For example, a significant fraction of HCC cases in South America manifests with unusual early-age onset [7, 8]. That is especially prevalent in the Andean Natives of Peru, in which non-cirrhotic, non-fibrolamellar HCCs develop in young patients with underlying infection by HBV subtype F1b [9, 10]. In these tumors, the DNA repair machinery is constitutively activated in liver cancer cells, despite a low HBV replication level and a low-to-moderate mutation rate at major gene targets [10, 11]. Intriguingly, a similar early-age onset of HCC, associated with the same clade of HBV, has also been described in Alaskan Native people, 10,000 km distant from the Andean communities of Peru [12]. This observation has raised the hypothesis of a particular dynamics in liver cancer shared by people with Indigenous American ancestries [8].

People with Indigenous ancestry remain starkly underrepresented in cancer genomics studies, which eventually limits the usefulness of available molecular classifications for these populations [13, 14]. Yet, it has been demonstrated that cancer biology is impacted by ancestry [15, 16]. The Peruvian population retains among the highest proportions of Indigenous American genetic architecture of all Latin Americans [17–19]. Accordingly, the peculiar presentation of HCC in the Andean Native people of Peru supports the idea that liver cancer pathogenesis might be modulated by the patients’ Indigenous American background, either through their genetic architecture or by anthropological determinants conditioning individual exposome [20]. However, establishing a link between disease presentation and ethnic or genetic background still requires to characterize the molecular hallmarks of these tumors [21, 22].

Here, we conducted an integrative analysis of gene expression and DNA methylation in HCC developed by patients from Peru. We report an original model of DNA hypermethylation associated with a gene expression signature that does not conform with the current molecular classification of HCCs. Altogether, our findings uncover a major role for anthropological background in molecular oncology with the characterization of a clinically relevant molecular subtype of HCC in patients with Indigenous American ancestry. To the best of our knowledge, the present study represents the first integrative genomics characterization of a molecular subtype of cancer that preferentially affects people with Indigenous ancestry.

## RESULTS

### Peruvian HCC coincides with Indigenous American ancestry and exhibits age-related features

**Figure 1:**
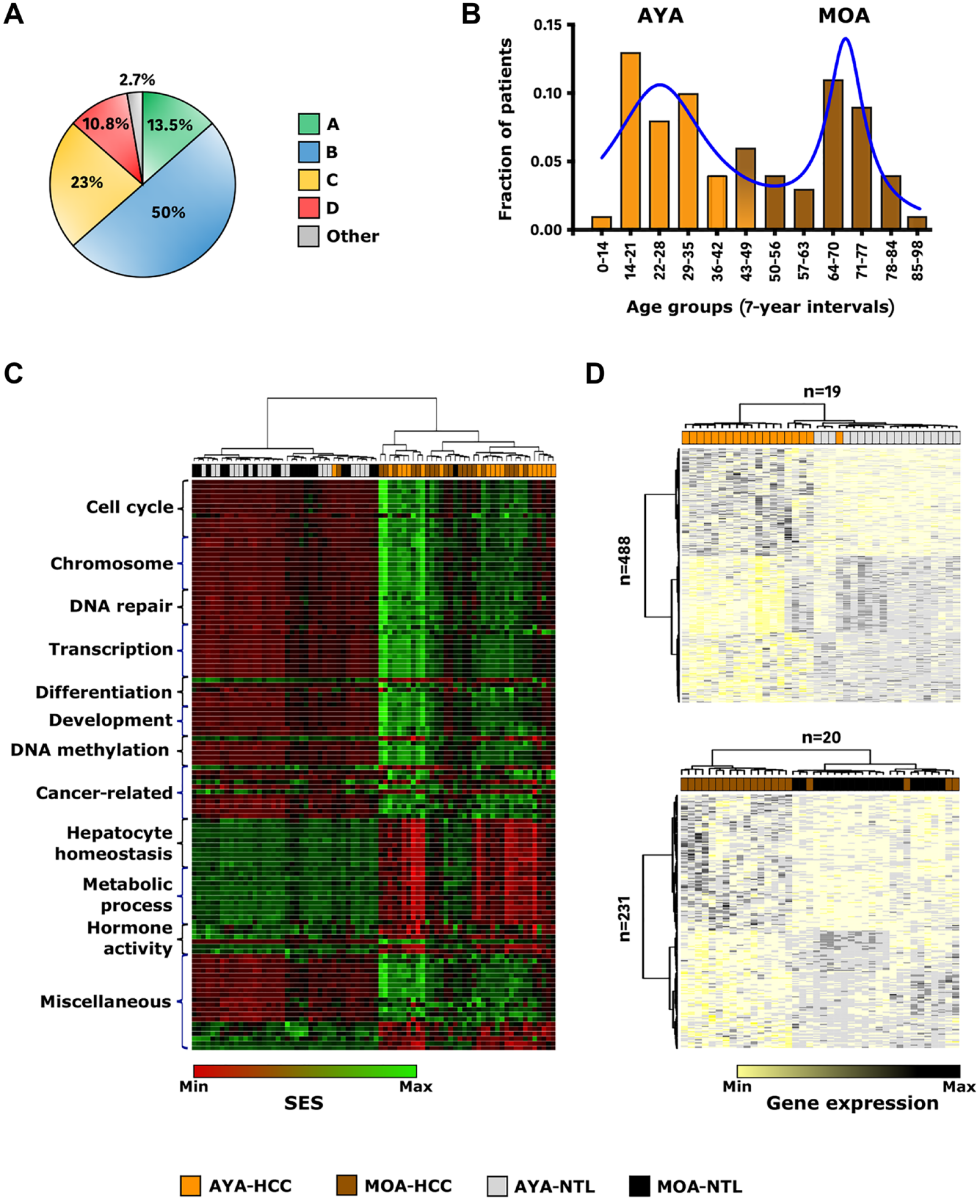
Indigenous American ancestry coincides with early-age onset of HCC and a peculiar transcriptome landscape. (**A**) Pie chart representing the distribution of the 74 Peruvian HCC patients by mtDNA haplogroups (Green: A; Blue: B; Yellow: C; Red: D; Grey: other). (**B**) Histogram showing the age distribution of the 74 Peruvian HCC patients at the time of diagnosis. X-axis shows age groups (7-year interval); Y-axis shows the fractions of patients according to the age groups (Orange: AYA; Brown: MOA). Solid delineation (blue) represents the histogram curve fitting. (**C**) Heatmap-based unsupervised hierarchical clustering of HCCs and NTLs in AYA (*n* = 19) and MOA (*n* = 20) (top dendrogram), produced from color-coded SES values of the 118 gene sets with significant differential HCC/NTL. (**D**) Hierarchically clustered heatmaps of HCC/NTLs (top dendrograms) in AYA (*n* = 19) (upper panel) and MOA (*n* = 20) (lower panel), produced from color-coded expression levels of the 488 AYA-specific and 231 MOA-specific DEGs (left dendrograms). (C, D) Black: MOA-NTL; Brown: MOA-HCC; Grey: AYA-NTL; Orange: AYA-HCC.

The 74 Peruvian patients with HCC included in the present study carried mitochondrial DNA (mtDNA) haplotypes of the four ancestral lineages (A–D) shared by Indigenous American populations (Figure 1A and Table 1) [23]. This series was in keeping with the bimodal age structure described in Peruvian HCC patients, with 52.7% patients represented by adolescents and young adults (AYA) aged 44 or younger (*n* = 39) and 47.3% middle and old age individuals (MOA) above age 44 (*n* = 35) (Figure 1B and Table 1) [24]. Tumor size normalization indicated a 3-fold elevation of AFP in AYA with 9,273 ± 14,613 ng/mL/tumor-cm, compared to the 3,305 ± 7,737 ng/mL/tumor-cm in MOA (*p* < 0.05). MOA presented a significantly higher, albeit still very mild, rate of liver cirrhosis than AYA (11.5% vs. 0%; *p* < 0.05). No fibrolamellar variant that usually occurs in younger individuals was reported in the series despite the significant fraction of AYA. Presence of HBV DNA was detected in at least one specimen of the matched pair of liver tissues (tumor and/or non-tumor) in 86.5% of patients (*n* = 64), with 92.3% of AYA (*n* = 36) and 80% of MOA (*n* = 28) (*p* > 0.05). Phylogenetic analysis of the HBV DNA sequences clustered all isolates of the series within the Indigenous subgenotype F1b [25]. Overall, this landscape has fueled the idea that two distinct age-related pathophysiological biologies coexist inherently in Peruvian communities with Indigenous American ancestry [8, 12].

**Table 1:** Baseline clinicopathologic features of the Peruvian HCC patients

Feature	Overall	AYA	MOA	Statistical significance
**Cohort**	74	39	35	
**MtDNA haplogroup**				*p* > 0.05^**^
A	10 (13.5%)	6 (15.4%)	4 (11.4%)	
B	37 (50%)	19 (48.7%)	18 (51.4%)	
C	17 (23%)	11 (28.2%)	6 (17.2%)	
D	8 (10.8%)	3 (7.7%)	5 (14.3%)	
ND	2 (2.7%)		2 (5.7%)	
**Age (years)**				*p* < 0.0001^*^
Mean ± SD	45.9 ± 22.5	27 ± 9	67 ± 11.3	
Median	44	27	68	
Range	[13–94]	[13–44]	[46–94]	
Interquartile range	43.2	13	16	
**Gender**				*p* > 0.05^**^
Female	23 (31%)	15 (38.5%)	8 (22.9%)	
Male	51 (69%)	24 (61.5%)	27 (77.1%)	
**Tumor size (cm)**				*p* > 0.05^*^
Mean ± SD	14.4 ± 7.5	14.2 ± 7.1	14.6 ± 8.1	
Range	[5–30]	[5–25]	[7–30]	
Interquartile range	8	8.5	8.5	
More than 10 cm	59 (79.7%)	31 (79.5%)	28 (80%)	
**Tumor growth pattern**				*p* > 0.05^**^
Acinar	2 (2.7%)		2 (5.7%)	
Solid	8 (10.8%)	4 (10.3%)	4 (11.4%)	
Trabecular	51 (68.9%)	27 (69.2%)	24 (68.6%)	
ND	13 (17.6%)	8 (20.5%)	5 (14.3%)	
**Tumor grade**				*p* > 0.05 ^**^
Well differentiated	2 (2.7%)	1 (2.5%)	1 (2.8%)	
Moderately differentiated	55 (74.3%)	32 (82.1%)	23 (65.7%)	
Poorly differentiated	15 (20.3%)	5 (12.9%)	10 (28.7%)	
ND	2 (2.7%)	1 (2.5%)	1 (2.8%)	
**Cirrhosis**				*p* < 0.05^**^
Positive	4 (5.4%)		4 (11.4%)	
Negative	70 (94.6%)	39 (100%)	31 (88.6%)	
**HBV DNA**				*p* > 0.05^**^
Positive	64 (86.5%)	36 (92.3%)	28 (80%)	
Negative	10 (13.5%)	3 (7.7%)	7 (20%)	

Footnote: Percentages are expressed as a ratio of the total patients investigated for the considered parameter. Tumor architecture and grading were defined according to the World Health Organization (WHO) Classification of Tumors, 5th Edition. Mean values are presented with ± standard deviation (SD). AFP: α-fetoprotein; HBV: hepatitis B virus; mtDNA: mitochondrial DNA; ND: not determined. ^*^ *t*-test; ^**^χ^2^ test.

### Pathway- and gene-centric approaches uncover a peculiar transcriptome landscape in Peruvian HCC

Transcriptome profiling was performed in a subset of 39 HCC and non-tumor liver (NTL) matched pair tissues (HCC/NTLs) for whom high-quality RNA was available [RNA integrity number (RIN) > 7]. We first followed a pathway-centric approach by using the previously described Sample Enrichment Score (SES) data mining method for scoring transcriptome hallmarks in single samples [26]. Data validation was carried out with random gene sets and dataset authentication by gender (Supplementary Figure 1). The SES method was applied to Peruvian HCC/NTLs using KEGG, Reactome, Gene Ontology-Cellular Component, and MsigDB Hallmarks. An age-stratification into AYA (*n* = 19) and MOA (*n* = 20) was used to explore the transcriptome according to the median (age 44 years old). Four comparisons were performed: (i) AYA-HCC vs. MOA-HCC, (ii) AYA-NTL vs. MOA-NTL, (iii) AYA-HCC vs. AYA-NTL, and (iv) MOA-HCC vs. MOA-NTL. Surprisingly, AYA vs. MOA (i and ii) did not exhibit noticeable differences, whereas a differential enrichment of 118 gene sets was found in HCC vs. NTL (iii and iv) (Supplementary Figure 2). Tumor gene set enrichments observed in age subsets were similar (Figure 1C and 1D). They corresponded to cell fate determination, cell cycle activation, epigenetic modifications, DNA damage response, as well as cancer-related pathways including Wnt/β-catenin, PI3K/AKT/mTOR, Slit2/Robo1, MYC, and ErbB2-ErbB3 axes (Figure 1C and Supplementary Table 1). Also, potentially important gene sets pertaining to iron metabolism, cellular senescence, sumoylation, and viral activity were enriched in tumors, albeit to a lesser extent. On the opposite, lower scores corresponding to depleted expression in HCC included hepatocyte identity or steroid signaling (Figure 1C and Supplementary Table 1). Of note, the retinoid signaling pathway score was also significantly lower in HCC (mean SES: 19.8) than in NTL (mean SES: 25) (*p* < 5.6E-10). Insight regarding immune status was gained by using gene sets designed to evaluate tumor infiltration with stromal or immune cells, as well as T-cell activation vs. immune escape effectors (IEGS33) or other effectors of the immunogenomic landscape [26–28]. None of the tumors belonged to the immune class, nor displayed fibrosis activation signature, further confirming the unusual microenvironment of Peruvian HCC [29, 30]. Interestingly, Peruvian HCC/NTLs displayed a low-level of inflammatory infiltration, at odds with prior data on the immune microenvironment in HCC (Supplementary Figure 3) [31, 32]. Finally, transcriptomic deconvolution identified the major fraction of leukocytes in HCC/NTLs as myeloid cells (Supplementary Figure 3) [28].

### Gene expression signature characterizes Peruvian HCC as a distinct molecular subtype

**Figure 2:**
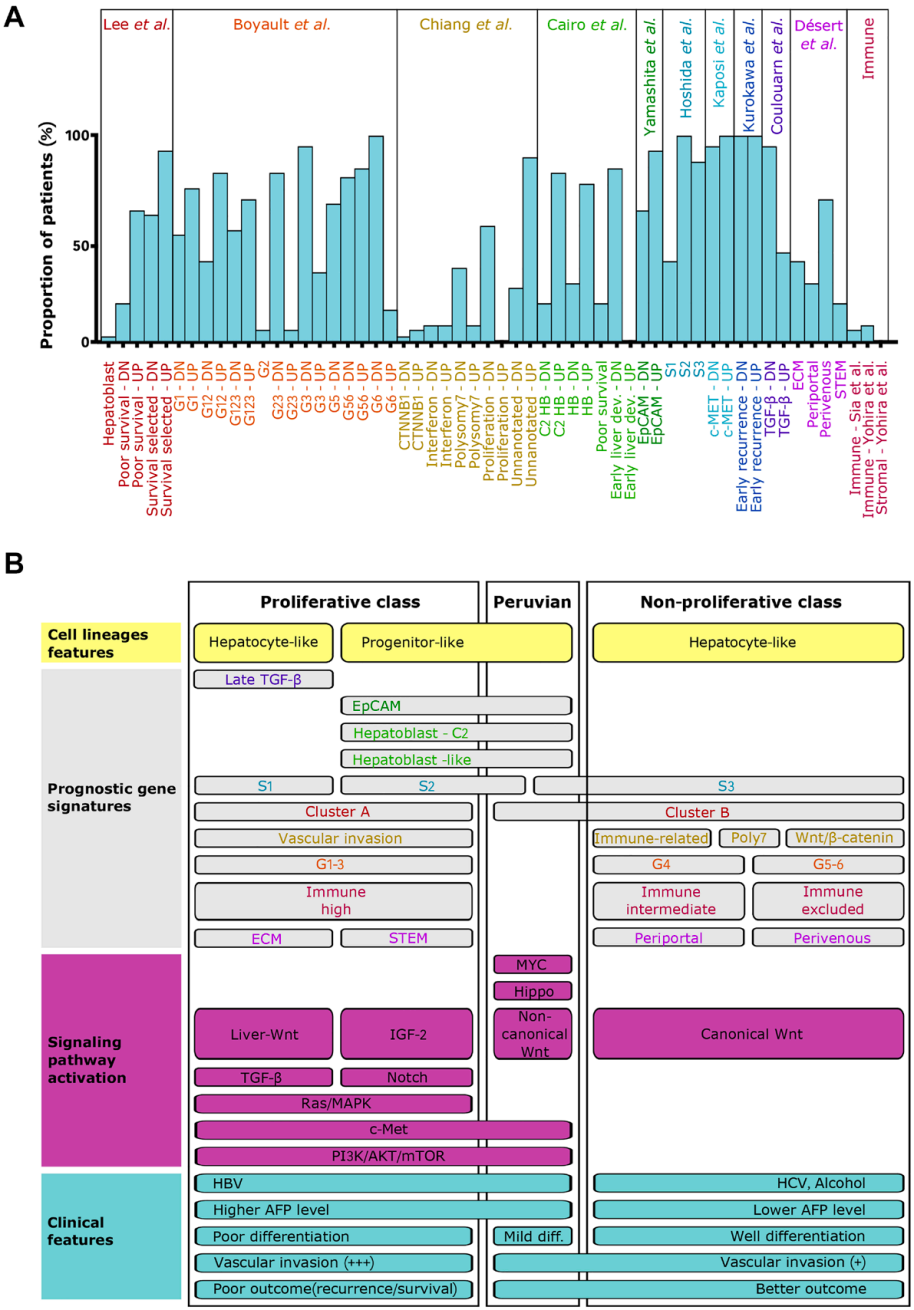
Peruvian HCC corresponds to an atypical positioning within the molecular classification. (**A**) Column chart showing the evaluation of published molecular signatures for HCC in Peruvian specimens. Molecular signatures were evaluated as the ratio of HCC/NTLs using SES (log2). DN: downregulated gene sets; UP: upregulated gene sets. (**B**) Schematic visualization of the unifying molecular classification of HCCs, integrating Peruvian specimens [3, 4]. Proliferative (left) and non-proliferative (right) classes are established according to prognostic gene signatures and signaling pathway activation. Associated clinical features are overlapped.

After batch-effect removal, gene expression data of the Peruvian patients were compared to HCC/NTL microarray datasets of 195 patients from other continental origins with clinical and demographic information (Supplementary Table 2). Out of the initial 118 gene sets, 56 were specific to Peruvian patients, pointing at a drastic loss of hepatocyte identity, including more extensive alterations in retinoids, carbohydrates, fatty, and bile acids metabolisms, or in xenobiotics-modifying enzymes (Supplementary Figure 4 and Supplementary Table 3). Downregulation of estrogens and somatotropin signaling pathways were also more pronounced in Peruvian tumors. Meanwhile, cell proliferation in Peruvian HCC was more dependent on activation of Hippo/YAP1 and c-Met/HGF proliferation pathways, DNA methylation and repair, and the activity of chromatin modifiers such as that of Polycomb Repressive Complex 2 (PRC2). The position of the Peruvian tumors within the molecular classification of HCCs was then explored using SES on previously reported signatures (Figure 2A) [3, 4]. Of note, Peruvian HCC transcriptomes blended two (S2 and S3) of the three reference clusters defined by Hoshida and collaborators [33]. Likewise, Peruvian tumors tended to be a composite of G1, G3, and G6 signatures defined by Boyault and collaborators [34]. Peruvian HCCs fell into the progenitor-like cluster of the proliferative class, but with *sui generis* peculiarities both in signaling pathway activation and clinical presentation (Figure 2B). Overall, Peruvian HCC did not conform with the molecular classes of HCC published previously. Moreover, Peruvian HCC displays relatively low rates of vascular invasion and recurrence despite a commonly heavy tumor burden (mean: 14 cm in diameter) [9, 35]. These findings indicate that the form of HCC developed by Peruvians with Indigenous American ancestry represents a molecular subtype divergent from the unifying molecular classification of HCCs [3, 4].

**Figure 3:**
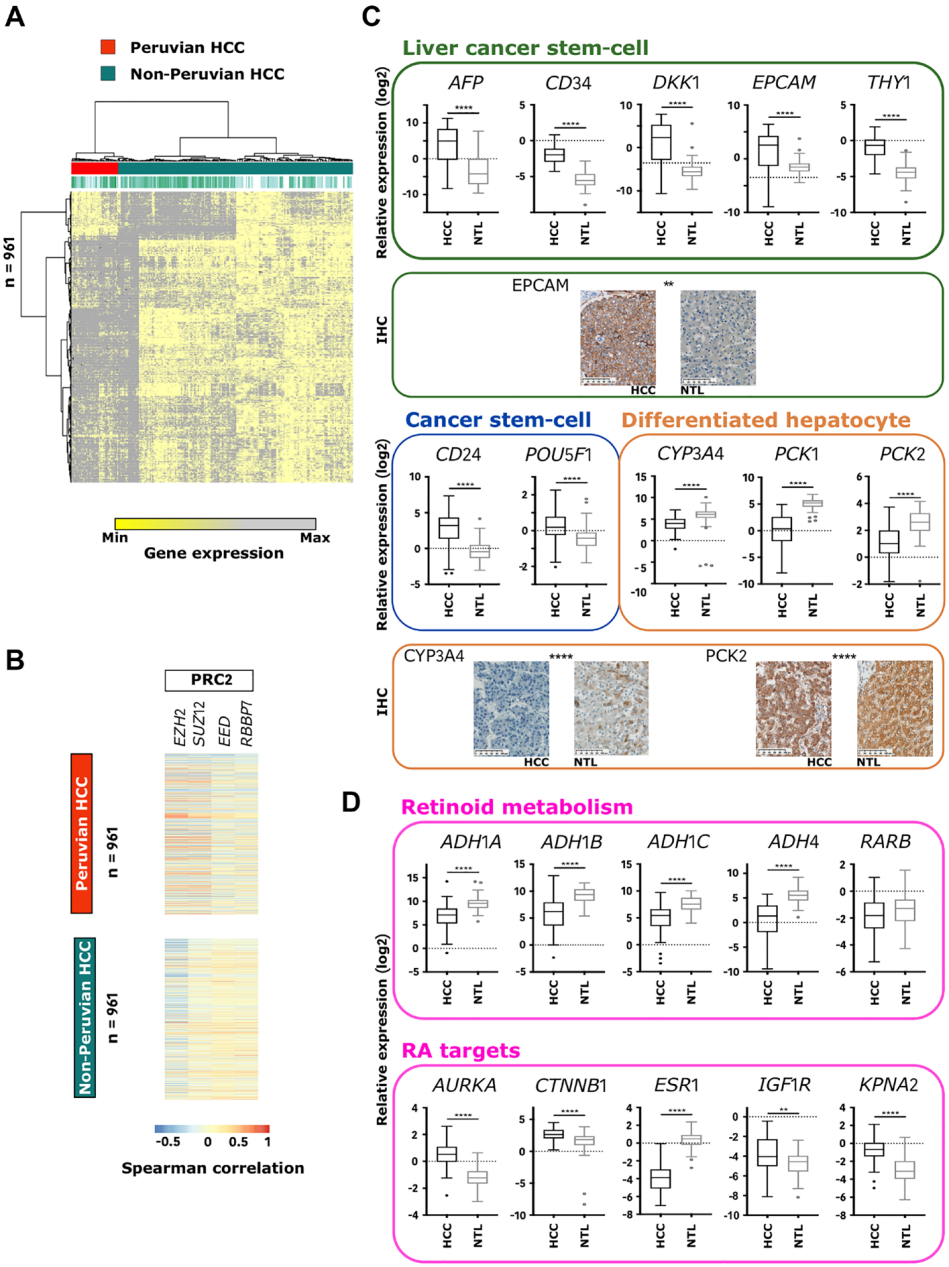
Amerind signature identifies Peruvian HCC as a distinct phenotypic cluster. (**A**) Heatmap-based unsupervised hierarchical clustering of HCCs from France (*n* = 81), Peru (*n* = 39), Taiwan (*n* = 97), and Turkey (*n* = 17) (top dendrogram), produced from color-coded expression levels of the 961 genes (left dendrogram) identified in the Amerind signature. Red: Peruvian HCCs; Jade green: HCCs from elsewhere. (**B**) Heatmap showing Spearman correlation coefficient between the 961 genes forming the Amerind signature and mRNA expression of the main PRC2 components (i.e., *EZH2*, *SUZ12*, *EED*, and *RBBP7*) in Peruvian and non-Peruvian HCCs. (**C**) Box-and-whisker plots representing relative gene expression (log2) in Peruvian HCC/NTLs (*n* = 65) of selected markers of liver cancer stem cell: *AFP*, *CD34*, *DKK1*, *EPCAM*, and *THY1*; cancer stem cell: *CD24* and *POU5F1*; and differentiated hepatocyte: *CYP3A4*, *PCK1*, and *PCK2*, measured by qPCR. Images: IHC captures; Scale bars: 100 μm. (**D**) Box-and-whisker plots representing relative expression (log2) in Peruvian HCC/NTLs (*n* = 65) of selected genes involved in retinoid metabolism: *ADH1A*, *ADH1B*, *ADH1C*, *ADH4*, and *RARB*; and retinoic acid (RA)-regulated targets: *AURKA*, *CTNNB1*, *ESR1*, *IGF1R*, and *KPNA2*, measured by qPCR. (C, D) Error bars represent confidence intervals. ^**^ *p* < 0.01; ^****^ *p* < 0.0001.

To further characterize this molecular subtype, HCC/NTL differentially expressed genes (DEGs) extracted from the 56 Peruvian-specific gene sets were scrutinized (AYA: 19,774 and MOA: 14,523). A derivation set was built by randomly selecting 21 Peruvian and 99 non-Indigenous American HCC datasets; and the discovery set was made with the remaining 18 Peruvian and 96 non-Indigenous American HCCs. A 961 gene signature was defined (hereinafter referred to as “Amerind signature”), of which 806 were upregulated and 155 downregulated in Peruvian HCC (Figure 3A and Supplementary Table 4). A major subset (48%) of downregulated DEGs defined hepatocyte identity (*n* = 74), whereas 16.4% of the upregulated DEGs were associated with cancer-related activation pathways (*n* = 136), 13.7% with chromatin organization (*n* = 111), and 12% with steroid hormone signaling (*n* = 97). The Amerind signature supported the idea that cell-fate maintenance defects are paramount in Peruvian HCC. Accordingly, 83 genes encoding for pivotal effectors in cellular allostasis and homeostasis, such as homeotic genes of *HOXA* and *HOXB* clusters, *PAX6* and *PAXIP1*, and 64 genes involved in the retinoid metabolism and liver specification, such as *ALDH1A2*, *ALDH1A3*, *RARB*, *RXRB*, and *RXRG*, were differentially expressed in Peruvian HCC [36]. Remarkably, gene expressions of the four core components of the PRC2 complex (i.e., *EED*, *EZH2*, *RBBP7*, and *SUZ12*) were significantly increased in Peruvian tumors (Supplementary Table 5). Some of the genes forming the Amerind signature (e.g., *BRCA1*, *FANCD2*, *MKI67*, *POLA1*, *POLD3*, *SOX9*, *YWHAZ*, and *ZNF207*) were displaying a stronger correlation of expression (either positive or negative) with PRC2 components in Peruvian HCC than in tumors from elsewhere, suggesting that PRC2 complex might be a key-driver of Peruvian liver tumorigenesis (Figure 3B). Quantitative PCR (qPCR) and immunohistochemistry (IHC) assays conducted in an independent cohort of Peruvian HCC patients (*n* = 65) confirmed downregulation of differentiated hepatocyte markers, upregulation of progenitor cell markers, and supported a dramatic disruption of retinoids signaling (Figure 3C and 3D, Supplementary Figure 5 and Supplementary Table 5) [37].

### DNA hypermethylation predominates in Peruvian HCC

**Figure 4:**
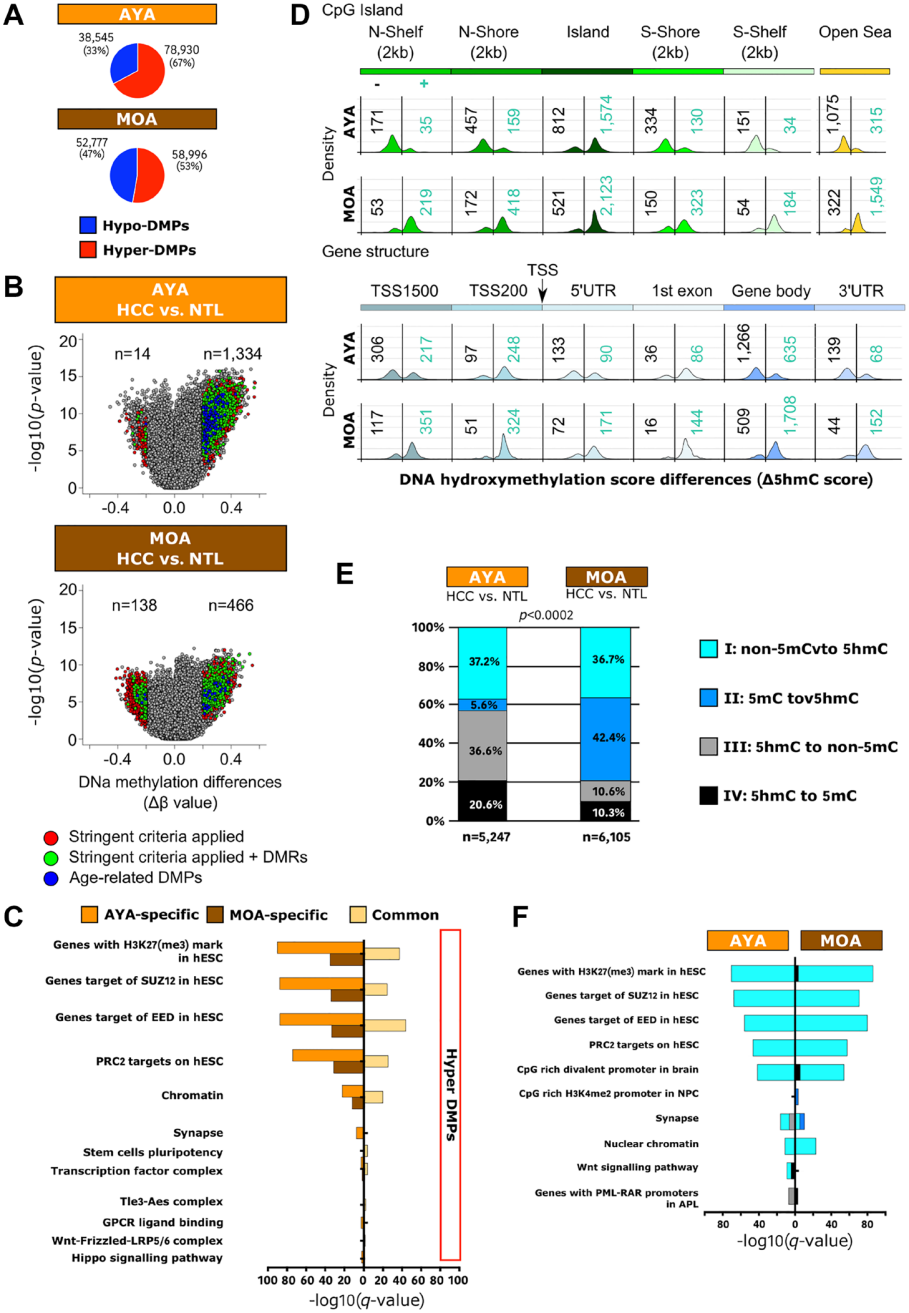
Peruvian HCC is associated with a genome-wide hypermethylation pattern, and DNA hydroxymethylation represents a relevant epigenetic mark in Peruvian HCC. (**A**) Pie charts representing proportions of hyper- and hypo-DMPs in AYA (upper panel) and MOA (lower panel). TSS: transcription start site; UTR: untranslated transcribed region. Blue: hypomethylated DMPs; Red: hypermethylated DMPs. (**B**) Volcano plots displaying HCC/NTL DNA methylation differences in AYA (upper panel) and MOA (lower panel). DMPs were identified by Δβ score ≥ |0.2|. Colored dots indicate significantly hyper- and hypo-methylated 5mCs when stringent criteria were applied [37]. Blue: age-related DMPs previously identified [39]; Green: 5mCs in differentially methylated regions (DMRs) defined as regions having at least two significant neighboring CpG sites displaying > 20% methylation differences in the same direction within a 250 bp region [37]; Red: DMPs. (**C**) Histogram presentation of the most significantly enriched gene ontologies associated with hyper-DMPs specific to AYA (orange) and MOA (brown), or common to both age groups (yellow), according to KEGG and Reactome terms. H3K27(me_3_): trimethylated lysine 27 in histone 3. (**D**) Schematic view of the number of 5hmC positions within CpG island (upper panel) and gene structure (lower panel) in Peruvian HCC/NTLs. Density plots show the frequency distribution of 5hmC gain (blue numbers) and loss (black numbers) in HCC cells for the given genomic region. (**E**) Bar plots representing the distribution of hydroxymethylation changes at individual CpGs in AYA (left) and in MOA (right). (**F**) Histogram presentation of the most significantly enriched gene ontologies associated with the four hydroxymethylation categories, according to KEGG and Reactome terms. NPC: neuronal precursor cells. (E, F) Black: 5hmC to 5mC; Dark blue: 5mC to 5mC; Light blue: non-5mC to 5hmC; Grey: 5hmC to non-5mC.

We previously determined that mutations rates at key-genes for HCC were rather low in Peruvian HCC (e.g., *TP53* and *TERT* are mutated in 14% and 17% of cases, respectively) [11]. Epigenetic changes appeared thus as plausible surrogate alterations in the liver carcinogenesis of Peruvian patients. Methylome profiling was performed on a larger series of 70 HCC/NTLs, including 36 AYAs and 34 MOAs. Mean β values indicating the degree of cytosine methylation (5mC) were compared between HCC and NTL. The high degree of cell purity monitored by SES in Peruvian HCC/NTLs ruled out 5mC contamination by leukocytes (Supplementary Figure 3) [27]. Upwards methylation shifts were overwhelming in Peruvian HCC (Figure 4A). Differentially methylated positions (DMPs) were then assessed with a *q*-value of 0.05 and a Δβ score that differed by at least 0.2 [38, 39]. Remarkably, the proportion of hypermethylated DMPs in AYA and MOA significantly contrasted with the corresponding values observed in studies conducted on HCCs of patients from North America, East Asia, and Europe (Supplementary Figure 6) [2, 39, 40]. Such trend toward higher levels of global genome methylation was observed in induced pluripotent stem cells (Supplementary Figure 6) [41]. In Peruvian HCC, hypermethylated DMPs were primarily detected in CpG islands within TSS 200 and TSS 1500 promoter regions; in contrast, hypomethylated DMPs were rather spotted on non-CpG island regions into gene bodies (Supplementary Figure 6). Previously reported stringent criteria were then applied to identify the most biologically meaningful DMPs in Peruvian HCC, meanwhile discarding age-related 5mCs [39, 42]. With these filtering criteria, hypermethylation was prevailing for 99% and 77% of DMPs in AYA and MOA, respectively (*p* < 0.0001) (Figure 4B). Functional enrichment analysis of AYA and MOA DMP-associated genes revealed DNA methylation shift on genes originally identified in human embryonic stem cells (hESCs) as targets of PRC2 presenting H3K27(me_3_) mark (Figure 4C). Less relevant classes were involved in transcription, cell potency, and cancer-associated pathways such as the Hippo/YAP1 and Wnt/β-catenin axes. Remarkably, some hypermethylated AYA-specific DMPs (*n* = 138) have been associated with neuronal differentiation known to represent a context of extreme epigenetic plasticity [43].

To provide further insights into the dynamics of DNA methylation marks, 5-hydroxymethylcytosine (5hmC) detection was performed on a subset of 31 HCC/NTLs, including 15 AYAs and 16 MOAs. 5hmC is commonly seen as an intermediate in active DNA demethylation, but it might also represent an intrinsically meaningful epigenetic mark *per se* [44]. The 5hmC score revealed that minor subsets of DMPs (4.2% in AYA and 5% in MOA) were hydroxymethylated in HCC or NTL tissues. The largest fractions of 5hmC in HCC/NTLs were detected in CpG islands and within open sea regions located into gene bodies in both age groups. However, a contrasting phenomenon was observed between AYA and MOA: cancer cells in AYA presented with a loss of hydroxymethylation along CpG islands (in shores, shelves, and open seas) located into TSS 1500 promoters, 3’ and 5’UTR regions, and gene bodies; whereas in MOA, cancer cells displayed a gain of hydroxymethylation on the same spots, suggesting an age-specific feature (Figure 4D). We then classified 5hmC positions in four categories: (I) unmethylated CpGs in NTLs converted to 5hmC in HCCs (non-5mC to 5hmC; AYA: 1,954 and MOA: 2,239), (II) methylated CpGs in NTLs converted to 5hmC in HCCs (5mC to 5hmC; AYA: 293 and MOA: 2,586), (III) 5hmC in NTLs converted to unmethylated in HCCs (5hmC to non-5mC; AYA: 1,919 and MOA: 647), and (IV) 5hmC in NTLs converted to methylated in HCC (5hmC to 5mC; AYA: 1,081 and MOA: 633). Categories III and IV were remarkably abundant in AYA, while categories I and II were predominating in MOA (*p* < 1E-04) (Figure 4E). *De novo* hydroxymethylation (Category I: non-5mC to 5hmC) was associated with the most consistent enrichments both in AYA and MOA, and outlined once more the crucial role of PRC2 in Peruvian HCC. Other ontological enrichment, both in AYA and MOA, pointed out an association between cell fate, CpG-rich bivalent promoter region in brain cells, and genes controlling synapse formation (Figure 4F). These findings suggest that, in Peruvian HCC, 5hmC acquisition might be part of a PRC2-driven cell reprogramming.

### Integrative genomics reveals PRC2-driven reprogramming of cancer cells in Peruvian HCC

**Figure 5:**
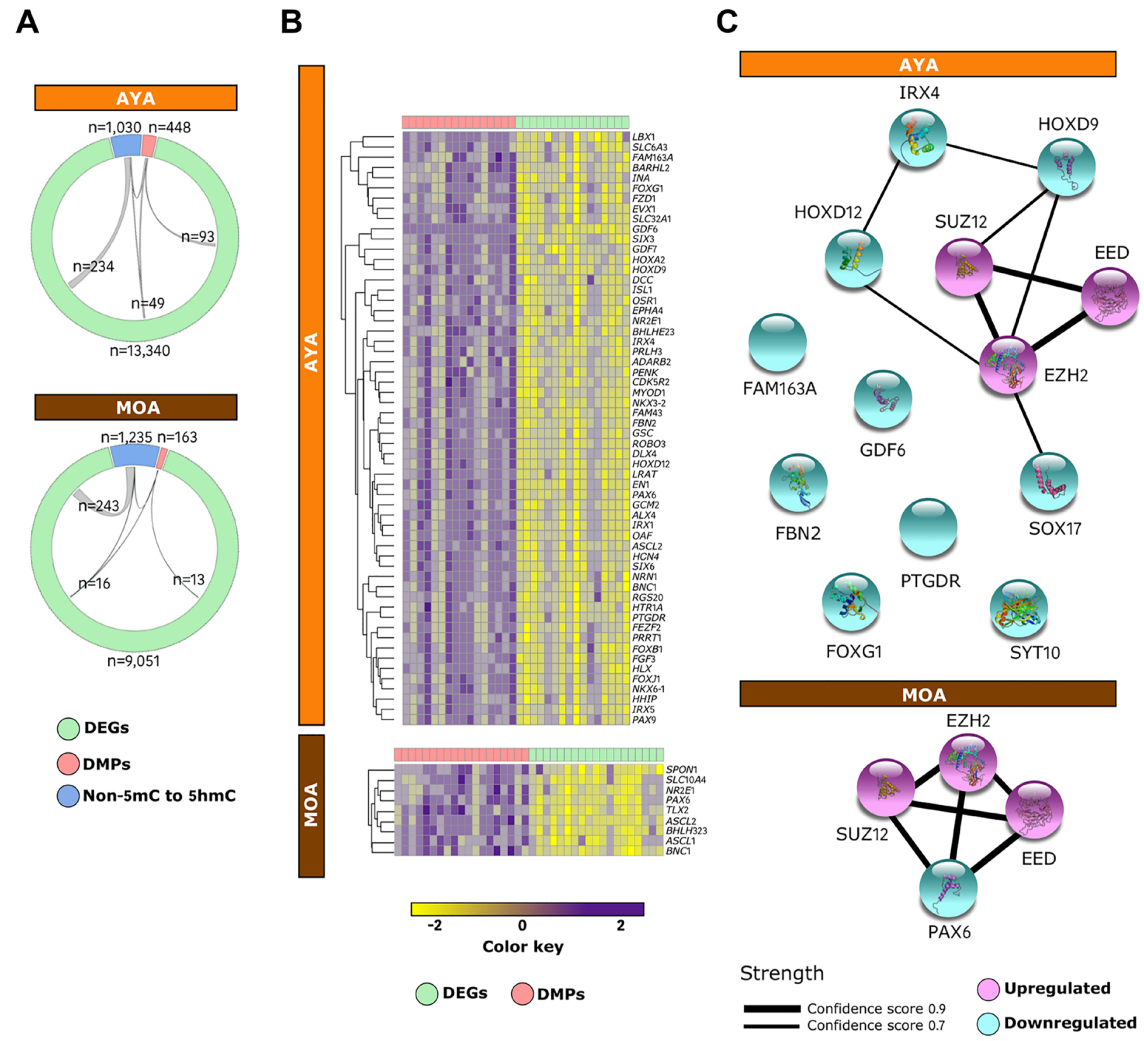
PRC2 complex mediates cancer cell reprogramming via DNA methylation in Peruvian HCC. (**A**) Circos plots displaying the relationships between downregulated DEGs (green), DMPs (red), and *de novo* 5hmCs (blue) in AYA (*n* = 15) (upper panel) and MOA (*n* = 16) (lower panel). (**B**) Heatmap of the integrative status of PRC2 targets in AYA (upper panel) and MOA (lower panel). The color key shows levels of expression (green) and methylation (red) independently scaled between -2 to 2. (**C**) Mapping of the protein–protein interaction network for PRC2 targets displaying high correlation (Spearman ρ > 0.5) between gene expression and DNA methylation in AYA (upper panel) and MOA (lower panel). Edges represent protein–protein associations meant to be specific and meaningful and line thickness indicates the strength of data support, with edge confidence scores ranging from 0.7 (thinner lines) to 0.9 (thicker lines). Nodes are filled with known or predicted 3D structure. Blue: downregulated genes; Purple: upregulated genes.

The integrative analysis uncovered an ample overlap between genes experiencing shifts in transcription and DNA methylation or hydroxymethylation, with a difference of magnitude between AYA (142 DMPs specific to AYA and 283 non-5mC to 5hmC) and MOA (29 DMPs specific to MOA and 259 non-5mC to 5hmC) (Figure 5A). Furthermore, within the subset of genes concomitantly hypermethylated and downregulated in AYA (51%) and MOA (48%), a large proportion were PRC2 targets (Figure 5B). Several genes showing the higher correlation between methylation and expression (Spearman ρ > 0.5) were known members of protein–protein networks important for development, including homeobox-containing *IRX4*, *HOXD9*, *HOXD12,* and *SOX17* in AYA and *PAX6* in MOA (Figure 5C). In contrast, DEGs associated with *de novo* hydroxymethylation process neither define a confident network nor an association with PRC2 complex (Supplementary Figure 7). This observation suggests that PRC2 complex influences primarily gene expression via DNA methylation, mediating cancer cell reprogramming and disease progression, especially in AYA; whereas in MOA, the role devoted to these marks, albeit similar, appears to be less extensive.

### *In vitro* targeting of the retinoid signaling

**Figure 6:**
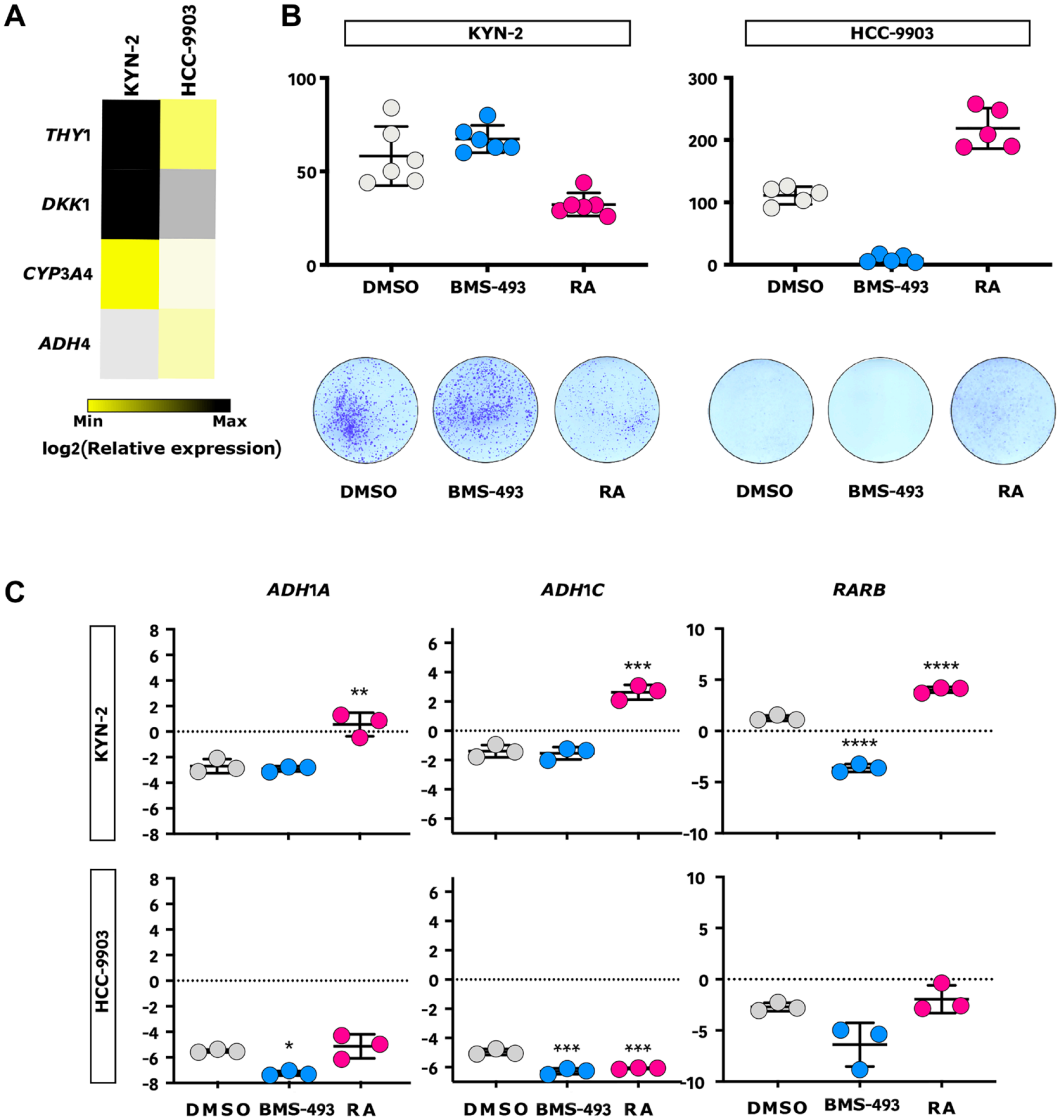
RA inhibits progenitor-like liver cancer cell growth *in vitro*. (**A**) Heatmap representation of the relative expression of *ADH4*, *CYP3A4*, *DKK1*, and *THY1* progenitor-like gene markers in KYN-2 and HCC-9903 cells measured by qPCR (*n* = 3). (**B**) Outline of the effect of RA, RA inverse agonist (BMS-493), and vehicle control (DMSO) on KYN-2 (left) and HCC-9903 (right) in a clonogenic assay. Upper panel: qualitative dot plots comparing the numbers of colony-forming units (CFUs) according to the treatment allocated; Lower panel: respective illustrations of CFUs. (**C**) Qualitative dot plots of the relative expression of three RA-mediated genes (i.e., *ADH1A*, *ADH1C*, and *RARB*) in KYN-2 (upper panel) and HCC-9903 (lower panel) according to the treatment allocated, measured by qPCR (*n* = 3). (B, C) Blue: BMS-493; Grey: DMSO; Red: RA. Error bars represent standard deviations. ^*^ *p* < 0.05; ^**^ *p* < 0.01; ^***^ *p* < 0.001; ^****^ *p* < 0.0001.

The genomic analysis of Peruvian HCC evidenced a weaker retinoid signaling signature in tumor cells, which could pinpoint novel targets and drugs for anticancer targeted therapy (Figure 1C and Supplementary Table 1) [45]. We hypothesized that this weaker retinoid signaling could be responsible for the increased proliferation; hence, the pharmacological response to RA should antagonize this process. Through a phenotypic exploration by qPCR of 57 different liver cancer cell lines, we selected hepatocyte-like HCC-9903 and progenitor-like KYN-2 cell lines, which display different patterns of gene expression for the retinoid signaling pathway, to assess their proliferative response to RA or RA inverse agonist BMS-493 stimulation in a clonogenic assay (Figure 6A and 6B, Supplementary Figure 8) [46]. In HCC-9903 cells, RA stimulated a doubling of CFUs, whereas BMS-493 abrogated them. By contrast, RA induced a 50% decrease of CFUs in progenitor-like KYN-2 cells, whereas BMS-493 was ineffective. These results were further confirmed by qPCR assessment of the RA-mediated modulation of *ADH1A*, *ADH1C*, and *RARB* gene expressions in KYN-2, taken here as a proxy for retinoid metabolism (Figure 6C) [47]. Progenitor-like cells, functionally close to Peruvian HCC, responded to RA stimulus, suggesting that RA-based treatment could efficiently contribute to reducing liver cancer cell proliferation in patients with Native American ancestry. This hypothesis is reinforced by the fact that serum retinol levels were depleted in 55.3% of individuals in an independent cohort of Peruvian HCC patients (*n* = 47), which included nine patients with severe retinol deficiency (Supplementary Table 6).

## DISCUSSION

HCC is one of the most heterogeneous forms of cancer, both in terms of molecular signature and phenotypic diversity. Considerable efforts have been made to elaborate on a clinically relevant molecular classification of HCCs [3, 4]. However, attempts at building a unifying classification, that includes the whole heterogeneity of HCC, will remain an ongoing process as long as some populations are underinvestigated [48, 49]. In this respect, there are still conspicuously underreported populations in the field of cancer research, among which Indigenous patients occupy a prominent position. The early-age onset HCC described in a significant fraction of Native American patients, originating from Alaskan and Andean regions, infected with the same endemic HBV subgenotype F1b, represents an illustration of this situation [10, 12].

In the present study, we report that Indigenous American haplogrouping coincides with peculiarities in both gene expression and DNA methylation in Peruvian HCC patients. Integrative genomics established that, while Peruvian HCC falls roughly into the progenitor-like cluster of the proliferative class (albeit in cahoots with cluster B of the non-proliferative class), it constantly displays idiosyncratic traits in signaling pathway activation [3, 4]. In that regard, Peruvian HCC forgoes some of the canonical hallmarks of the proliferative class (e.g., IGF2, Notch, Ras/MAPK, and TGF-β) [3, 4]. More importantly, the Amerind signature for HCC is remarkably enriched with genes involved in the Hippo/YAP1, MYC, and Wnt/β-catenin pathways, as well as in Polycomb epigenetic repressors and DNA repair effectors (e.g., *BRCA1*, *FANCD2*, and *TP53*). The significant gain of stem cell-like features observed at the transcriptome level in Peruvian HCC involves the activity of epigenome regulators, concomitant with a massive loss in hepatocyte markers, in the context of aberrant nuclear receptor signaling involving retinoids or steroids. From a molecular standpoint, Peruvian HCC features a homogeneous, albeit hybrid, phenotypic subtype between the proliferative and non-proliferative classes that were presumably mutually exclusive according to the classification of HCCs [3, 4]. Besides, Peruvian tumors appear to be also distinct from the cyclin-driven HCC subclass, which displays an activation of the ATR and E2F pathways and downregulation of MYC targets [50]. The molecular divergence of Peruvian HCC, with tumors from elsewhere, is evidenced by hierarchical clustering relying on a large and meaningful gene expression signature. Whether this molecular phenotype is due to anthropological specificities embedded in genome architecture, to extrinsic etiological cues, or to subtle interplays between both components remains to be ascertained. In this view, the distinctive tumor dynamics could result from an incomplete adaptation of people with Native American background to the endemic HBV subgenotype F1b [25, 51, 52].

The clinical epidemiology of HCC in Peru is characterized by the dual occurrence of an early-onset form of HCC in younger individuals, concomitant with a more conventional older population of patients [8, 24]. This presentation initially suggested two inherent age-related natural histories of the disease among the Peruvians. However, comparative transcriptome analysis between AYA and MOA uncovered a difference of degree rather than nature in terms of gene expression, instead indicating a biological continuum throughout the aging process. Withal, while 5hmC gain corroborated the functional enrichment analysis of the transcriptome, DNA methylation exhibited an age-dependent pattern with significantly more methylated CpGs in AYA compared to MOA. Interestingly, Peruvian HCC in AYA, and to a lesser extent in MOA, exhibited high levels of DNA methylation, contrasting with the global hypomethylation pattern considered as a hallmark of HCC and other carcinomas [5]. Altogether, these findings suggest that liver tumorigenesis in Peruvians is the outcome of a DNA methylation program disruption, especially in AYA. It uncovers a unique biological model of global DNA hypermethylation dynamics in cancer development, somewhat reminiscent of that observed in the reprogramming of cells to pluripotency [41]. In this context, tumor cells might originate from either misprogrammed liver progenitors unable to implement a full-fledged hepatic specification; or an epithelial/mesenchymal plasticity fostered by inappropriate microenvironmental cues that trigger the conversion of differentiated liver cells into cancer cells [53, 54].

There are some limitations to recognize in the present study. Because of the clinical epidemiology of HCC in Peru, we did not have the opportunity to incorporate in our experimental design HCC patients with anthropological backgrounds other than Native American ethnicity. Therefore, it remains difficult to come to a decisive conclusion on the respective contributions of human genome architecture and exposome in the molecular epidemiology of HCC in Peru. The comparison with patients from East Asia, the Middle East, and Western Europe was achieved using publicly available datasets produced in different periods. Although we used up-to-date normalization tools to avoid batch effects, we cannot rule out the fact that this comparison is not free of bias [55]. Nonetheless, the present study stresses the necessity to justly reconsider the potentially prominent roles played both by human genome architecture and biogeography in the molecular epidemiology of cancer affecting underreported minorities and Indigenous patients, notably in low- and middle-income countries [56].

Finally, our findings have implications for the development of therapeutics tailored to this newly identified molecular subtype of HCC. Some authors have discussed the role of RA action in the prevention and treatment of MYCN-positive liver cancer stem cells, and it is apparent that a profound alteration of retinoid signaling is a crucial characteristic of Peruvian HCC [45]. Herein, clonogenic assays have demonstrated *in vitro* the ability of RA to block the proliferation of HCC cells that have a constitutive defect of the retinoid signaling pathway. This preliminary result would represent a new avenue in molecularly-targeted therapy: the reversal and maintenance of HCC cells into a metastable innocuous hepatocyte phenotype by active, RA-mediated DNA methylation reprogramming [6, 45, 57]. The present study establishes a foundation for the dissection of the functional importance of RA-mediated epigenetic control in HCC and therapeutics tailored to patients with Indigenous American ancestry.

## MATERIALS AND METHODS

### Study conduct

Written informed consent was provided by patients or legal guardians for their information and samples to be stored and used for research. The study was approved by the Ethics Evaluation Committee of the National Cancer Institute of Peru (INEN), protocol N° 10-05.

### Study design and sampling

The study was conducted retrospectively on a series of 74 surgical specimens resected from patients treated at INEN (Lima, Peru) between August 2006 and August 2016. Patients with HCC underwent anatomic liver resection as previously described [35]. None of the patients was treated with chemotherapy or radiation prior to tumor resection. NTLs were obtained from tumor-free margins of the resected surgical pieces. About 50 mg of HCC/NTL were promptly harvested after surgical removal, flash-frozen with liquid nitrogen, and stored at –150°C in the INEN Cancer Research Biobank. Comprehensive histopathological diagnosis and patient follow-up were performed as previously described [9, 35]. Different subsets of HCC/NTLs for profiling transcriptome (*n* = 39), methylome (*n* = 70), and hydroxymethylome (*n* = 31) were selected *ad rem* in keeping with the age structure and the age-related biomedical features.

### Nucleic acids extraction

Tissues were pounded under liquid nitrogen and then digested at 37°C for 8 hours in RNase-free tissue lysis buffer containing proteinase K and sodium dodecyl sulfate (SDS). Genomic DNA was extracted twice with phenol and once with chloroform, precipitated in ethanol, and resuspended in TE buffer (10 mM Tris; 0.1 mM EDTA, pH 8.0). The resulting DNA pools were quantified using Qubit^®^ dsDNA Broad-Range Assay Kit (Invitrogen). Total RNA was isolated from flash-frozen HCC/NTLs using TRI Reagent^®^ (Sigma-Aldrich) and Lysin Matrix D homogenization system (MP Biomedicals). The RNA pools were quantified using Qubit^®^ RNA Broad-Range Assay Kit (Invitrogen). RIN was assessed using the RNA 6000 Nano LabChip^®^ Kit and a 2100 Bioanalyzer System (Agilent Technologies).

### Mitochondrial haplogroup determination

Primers mt16023 and mt16422 were designed to amplify the mtDNA D-loop hypervariable region 1 (HVR1) containing genetic variants for Indigenous American mtDNA haplogroup determination (Supplementary Table 7) [58]. PCR reactions were carried out using 10 ng DNA in a 50 μL reaction volume with 2 U of Platinum™ Taq DNA polymerase (Invitrogen) and 20 pmols of primers. Touchdown amplification was performed with an initial denaturation temperature at 94°C for 2 min, followed by 18 cycles of 94°C for 15 s, annealing temperatures starting at 70°C for 15 s (decreasing at 3°C after every three cycles), 72°C 30s for extension. This step was followed by 20 cycles of 94°C for 15 s, 50°C for 15 s, 72°C for 30 s, and finally 72°C for 3 min. PCR amplicons were sequenced according to a Sanger method and haplotypes were determined using Mitomaster sequence analysis tool version Beta 1 [59].

### Hepatitis B virus DNA detection and phylogeny

HBV detection and phylogeny was performed as previously described [10]. Briefly, HCC/NTLs were screened for the presence of HBV DNA on a QX200™ Droplet Digital™ PCR System (Bio-Rad) using the TaqMan^®^ Pathogen Detection Assay Pa03453406_s1 and the Human TaqMan^®^ Copy Number Reference Assay as a reference (both Thermo Fisher Scientific). Reaction mixtures consisted of 100 ng of total DNA in a 20 μL reaction volume with 10 μL of QX200™ ddPCR™ EvaGreen^®^ Supermix (Biotium) and 1x primers, blended with 70 μL of QX200™ Droplet Generation Oil (Bio-Rad), according to the manufacturer’s workflow. PCR reactions were performed in duplex with an initial denaturation temperature at 95°C for 10 min, followed by 40 cycles at 94°C for 30 s with a 2.5°C/sec ramp rate, 59°C for 1 min with a 2.5°C/sec ramp rate, 98°C for 5 min, and hold at 4°C. Data were analyzed using the QuantaSoft™ software version 1.7 (Bio-Rad), with auto-analysis settings for duplex experiment. HBV sequences were produced using the BigDye™ Terminator v3.1 Cycle Sequencing Kit (Applied Biosystems). HBV phylogeny was computed using Kimura two-parameter model and neighbor-joining method on the Molecular Evolutionary Genetics Analysis (MEGA) software version 7 [60].

### Transcriptome profiling

RNA profiling was performed using GeneChip™ Human Transcriptome Array 2.0 and GeneChip™ WT PLUS Reagent Kit (Applied Biosystems), according to the manufacturer’s instructions. Alternatively, transcriptome datasets for comprehensive comparative analyses were obtained from Gene Expression Omnibus (GEO) for HCC/NTLs. Gene expression data normalization and batch correction were performed using the Robust Multichip Average (RMA) and ComBat algorithm (sva R package), respectively [55]. These datasets were then collapsed using Fred’s Softwares algorithm (collapse_genes-09). A pathway-centric approach using SES was applied to our dataset based on four curated databases by Fred’s Softwares: KEGG, Reactome (reactome_2018803), Gene Ontology-Cellular Component (C5_CC), and MSigDB Hallmarks (H), according to the recommended workflow (two-tailed *t*-test with *p*-value < 1.7E-05 as significance level and fold change < 0.7 and > 1.5 as thresholds) [26]. Fred’s Software source codes and databases are available at: https://sites.google.com/site/fredsoftwares/products. A gene-centric approach was applied to identify DEGs using limma R package with a *q*-value of 0.05, following the recommended workflow [61]. The identification of the Amerind signature was carried out using the Signature Evaluation Tool (SET) with the lowest error rate at 0.075, as recommended [62]. Reference signatures (C6) from the MSigDB version 7.1 were evaluated on HCC/NTLs using SES [63].

### Methylome profiling

DNA methylation profiling was performed with bisulfite treatment using the Infinium^®^ HumanMethylation450K and MethylationEPIC BeadChip arrays (Illumina), according to the manufacturer’s instructions. DNA methylation patterns were normalized using Noob correction method [64]. DMPs were identified using minfi R package with a *q*-value of 0.05, following the recommended workflow [38]. Stringent criteria were used to account for the most biologically meaningful DMPs, as described previously [39]. These filtering criteria applied to each DMP consisted in: (1) the mean Δβ value in HCC/NTL was ≥ 20%; (2) More than 70% of the HCCs had β values greater than 2 standard deviations above the mean methylation level of all 70 NTLs; and (3) the mean Δβ value for NTLs was ≤ 25% (for hypermethylated sites) or ≥ 25% (for hypomethylated sites). DNA hydroxymethylation profiling was performed with oxidative bisulfite treatment using the Infinium^®^ MethylationEPIC BeadChip array. 5hmC detection was achieved according to the 5hmC-score method identifying 5hmC positions between the bisulfite- and oxidative bisulfite-treated replicates within each sample, as previously reported [65]. Briefly, hydroxymethylated CpGs were identified with a 5hmC-score ≥ 0.3 in at least three samples. 5hmC-scores were determined independently in HCC and NTL tissues. Finally, definite 5hmC were defined as those falling in the third quartile of the 5hmC-score density distribution, which encompassed more than 80% of identified CpGs. Gene list functional enrichment analysis was performed using ToppFun on four curated databases: KEGG, Reactome, Gene Ontology-Cellular Component, and MsigDB Hallmarks, with a Bonferroni *q*-value of 0.05.

### Integrative analysis of DNA methylation and gene expression data

Associated gene symbols were used to merge DEGs with 5mC positions and/or with non-5mC to 5hmC positions in R environment, according to the HUGO Gene Nomenclature Committee (HGNC) [66]. Spearman’s ρ was calculated between gene expression and β value or 5hmC-score for each DEG/5hmC-specific or DEG/non-5mC to 5hmC dyad, respectively. Those genes with Spearman’s ρ > 0.05 were evaluated on STRING version 10 for protein–protein interaction network and enrichment analysis, with default setting [67].

### Quantitative transcript detection

qPCR assays were performed as previously described [11], with the following modifications: relative gene expression was calculated according to the ΔΔC_T_ method using the geometric mean of five housekeeping gene expression as references: *TRIM44*, *HMBS*, *LMF2*, *CIPC*, and *EME2*. These reference genes were selected from the transcriptome profiling of the 39 Peruvian HCC/NTLs, to which geNorm and NormFinder algorithms were applied [68]. The list of DNA primer pairs for qPCR is provided in Supplementary Table 7.

### Immunohistochemistry and blood tests

Paired tissues were formalin-fixed, paraffin-embedded as previously described [9]. IHC was performed on HCC/NTL sections at the Histo Pathology High Precision (H2P2) platform with certification to International Organization for Standardization (ISO) 9001 (Rennes, France). The list of antibodies used for IHC is provided in Supplementary Table 7. Serum AFP levels were monitored from blood samples using electro-chemiluminescence immunoassay (ECLIA); and serum retinol levels were measured retrospectively using high-performance liquid chromatography (HPLC).

### Clonogenic assay

Hepatocyte-like HCC-9903 and progenitor-like KYN-2 HCC cell lines were cultivated using Gibco™ DMEM, High Glucose, GlutaMAX™ Supplement with 10% fetal bovine serum, and 100 U/mL Gibco™ Penicillin-Streptomycin at 37°C in a 5% CO_2_ humidified atmosphere. Clonogenic assays were performed as previously described [69]. Briefly, 5E+03 HCC-9903 and 7.5E+02 KYN-2 cells/well were respectively incubated for 14 days in 6-well plates either with 5 μM RA (Sigma), 5 μM RA inverse agonist BMS-493 (Tocris Bioscience), or DMSO as vehicle control. Cells were then washed three times with cold PBS, fixed with ice-cold 100% methanol, and stained for 30 minutes with 3 mL of 0.5% crystal violet solution (0.5% crystal violet; 4% formaldehyde; 30% ethanol; 0.17% NaCl). The supernatant was then discarded and CFUs were counted using a stereo-microscope. Assays were conducted in duplicate.

### Statistics

Statistical analysis was computed using the R software environment version 3.5.3. Box plots and heatmaps with *t*-test and χ^2^ test were charted using the Prism software version 8 (GraphPad). Statistical significance was evaluated one- or two-tailed according to the circumstances.

### Data availability

The datasets generated during this work have been deposited in the GEO repository with accessions GSE111580, GSE136247, GSE136319, GSE136380, and GSE136583. Additional analyzed datasets are available in GEO with accessions GSE141521, GSE17548, GSE37988, GSE45436, GSE56588, GSE62232, and GSE63898; as well as in the database of Genotypes and Phenotypes (dbGaP) with the study accession phs000178.v11.p8 (project ID: TCGA-LIHC). HBV sequences are available in the European Nucleotide Archive (ENA) repository with the secondary study accession ERP023329.

## SUPPLEMENTARY MATERIALS





## Abbreviations

5mC: 5-methylcytosine
5hmC: 5-hydroxymethylcytosine
AFP: α-fetoprotein
AYA: adolescents and young adults
CFU: colony-forming unit
DEG: differentially expressed gene
DMP: differentially methylated position
DMR: differentially methylated region
GEO: Gene Expression Omnibus
H3K27(me_3_): trimethylated lysine 27 in histone 3
HBV: hepatitis B virus
HCC: hepatocellular carcinoma
HCC/NTL: tumor and non-tumor matched pair tissues
hESC: human embryonic stem cell
IHC: immunohistochemistry
MOA: middle and old age individuals
mtDNA: mitochondrial DNA
NTL: non-tumor liver
PRC2: polycomb repressive complex 2
qPCR: quantitative PCR
RA: retinoic acid
RIN: RNA integrity number
SES: sample enrichment score
TSS: transcription start site
UTR: untranslated transcribed region
