# Supplementary material for: Global DNA hypermethylation pattern and unique gene expression signature in liver cancer from patients with Indigenous American ancestry
Source: Oncotarget. 2021 Mar 2;12(5):475–92. doi: 10.18632/oncotarget.27890 (PMC7939527; doi:10.18632/oncotarget.27890)
Supplement: Supplementary file 1 [file oncotarget-12-475-s001.pdf]

Global DNA hypermethylation pattern and unique gene expression signature in liver cancer from patients with Indigenous American ancestry

SUPPLEMENTARY MATERIALS

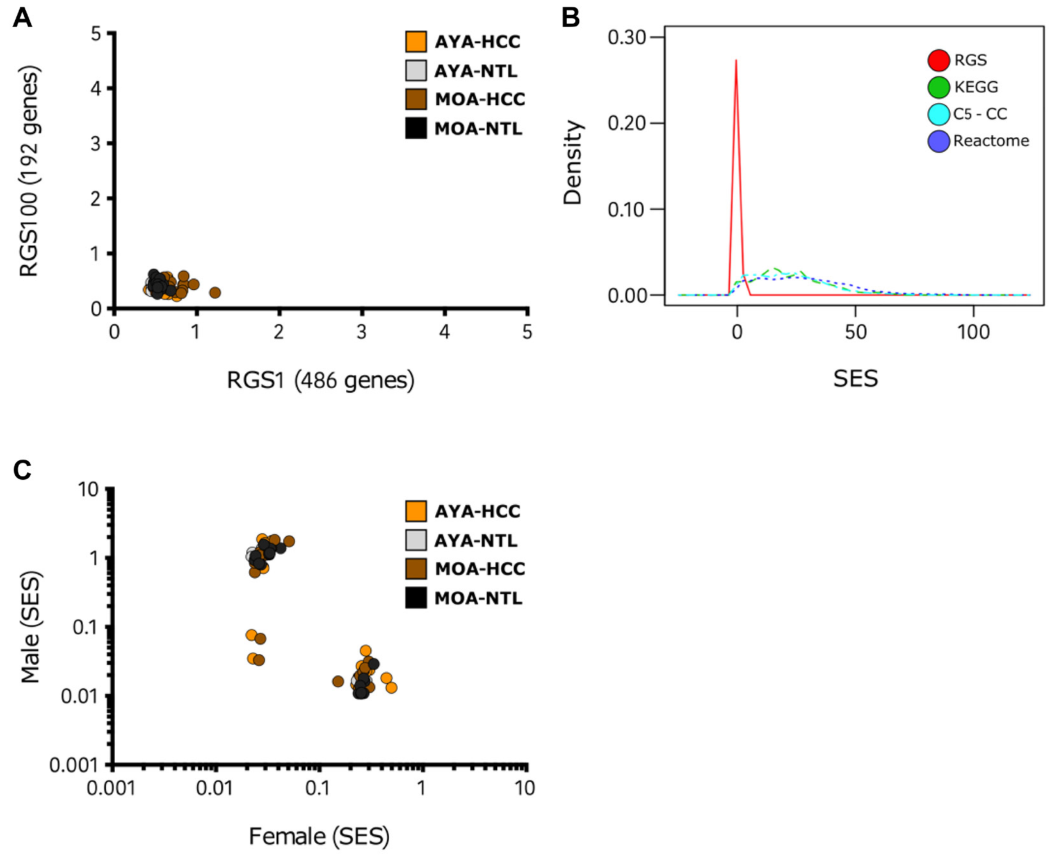

Supplementary Figure 1: Transcriptome quality control. (**A**) Scatter plot from SES with two random gene sets (RGS) – RGS1 (X-axis) and RGS100 (Y-axis) – in AYA (*n* = 19) and MOA (*n* = 20) [1]. (**B**) Control curve chart of SES (density) for 1,000 control RGS (red solid curve) and all gene sets from curated databases (dashed curves). Dark blue: Reactome; Green: KEGG; Light blue: Gene Ontology-Cellular Component (C5-CC). (**C**) Scatter plot from SES of female (X-axis) and male (Y-axis) gene sets in HCC/NTLs from AYA and MOA patients [1]. (A, C) Black: MOA-NTL; Brown: MOA-HCC; Grey: AYA-NTL; Orange: AYA-HCC.

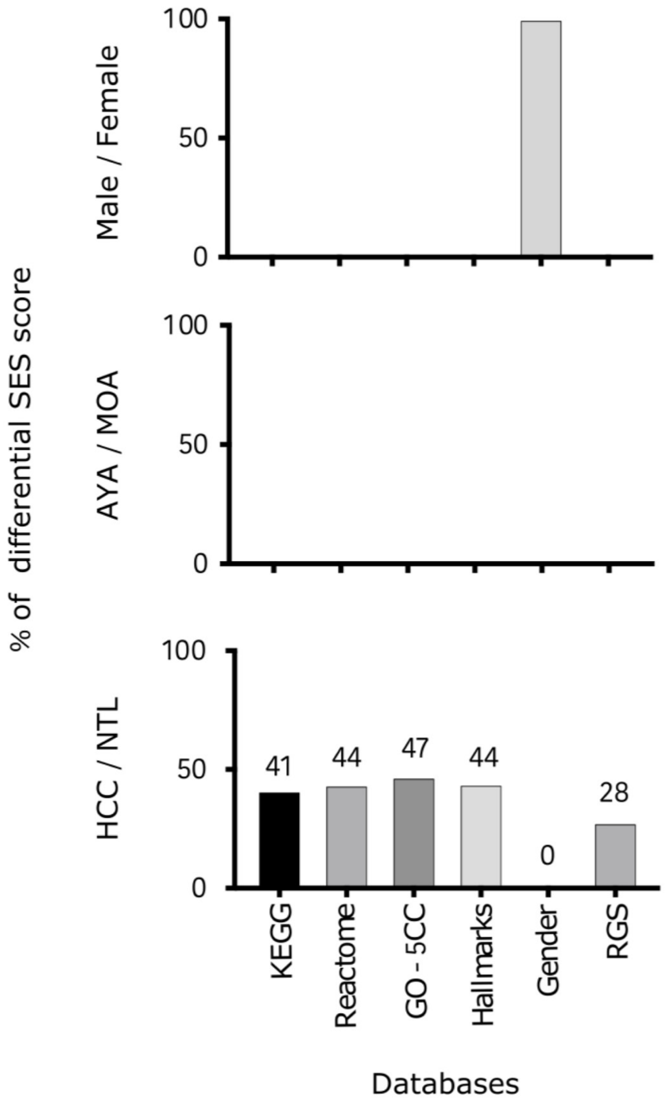

Supplementary Figure 2: Percentages of gene sets with significant different SES in HCC/NTLs (*n* = 39) according to four curated databases. [i.e., KEGG, Reactome (reactome_2018803), Gene Ontology-Cellular Component (C5_CC), and MSigDB Hallmarks (H)]. SES analysis was performed according to the specified criteria: gender (Male vs. Female) (upper histogram), patient age groups (AYA vs. MOA) (middle histogram), and tissue types (HCC vs. NTL) (lower histogram). A corrected *p-value* lower than 3.33E-06 was used as threshold.

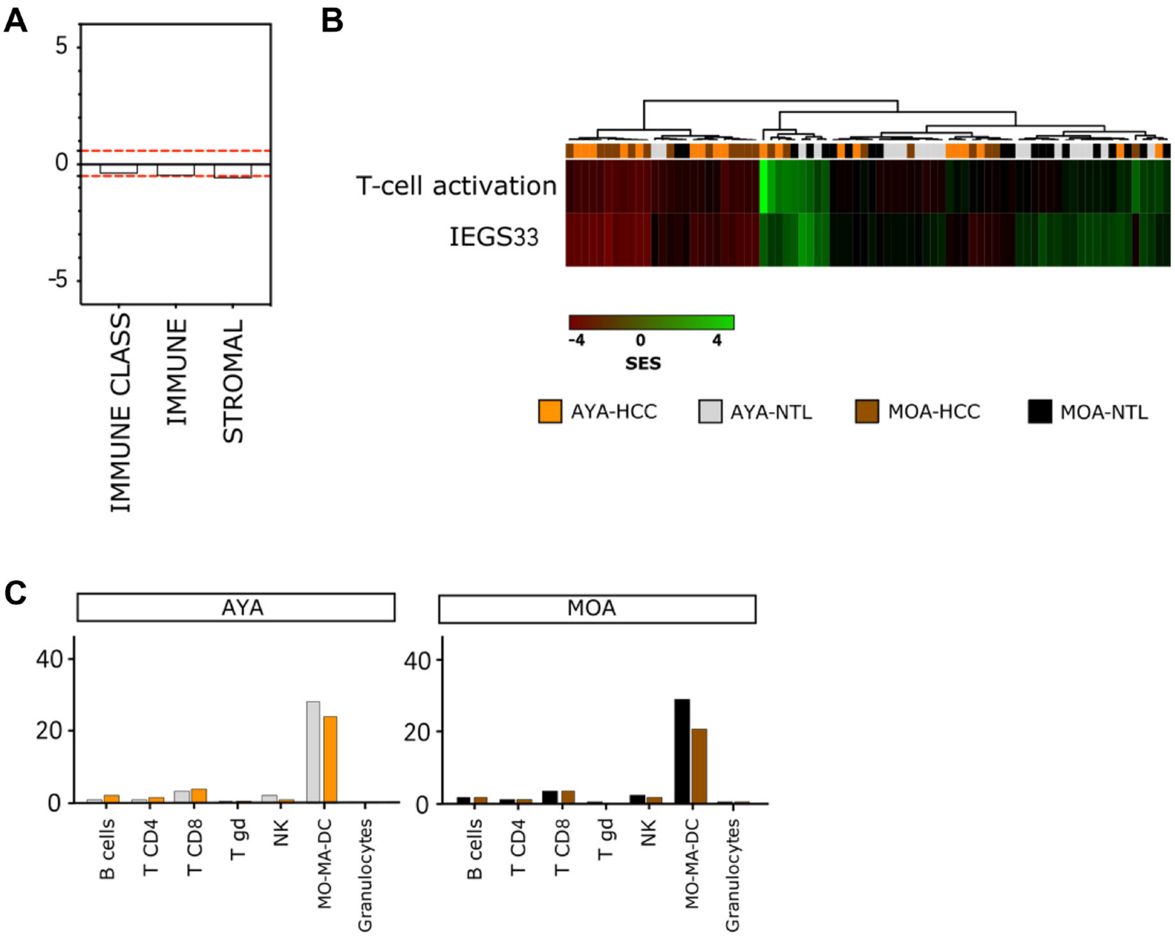

Supplementary Figure 3: Immune cell expression profiling in Peruvian HCC according to SES analysis. (**A**) Column chart showing the evaluation of published molecular signatures for immune class of HCC (immune class) and immune infiltration (immune and stromal) [2–4]. Molecular signatures were evaluated as the ratio of HCC/NTL using SES (log2). Statistical significance threshold was set at ± log2 fold changes (corrected *p-value*) (red dashed lines). (**B**) Heatmap-based unsupervised hierarchical clustering of HCCs and NTLs in AYA ($n = 19$) and MOA ($n = 20$) (top dendrogram), produced from SES of T-cell activation and immune escape effectors (IEGS33) gene sets in HCCs and NTLs [1]. (**C**) Histogram representations of leukocytes composition by transcriptome deconvolution in HCC/NTLs of AYA and MOA. MO-MA-DC: myeloid lineage; NK: natural killer cells [5]. (B, C) Black: MOA-NTL; Brown: MOA-HCC; Grey: AYA-NTL; Orange: AYA-HCC.

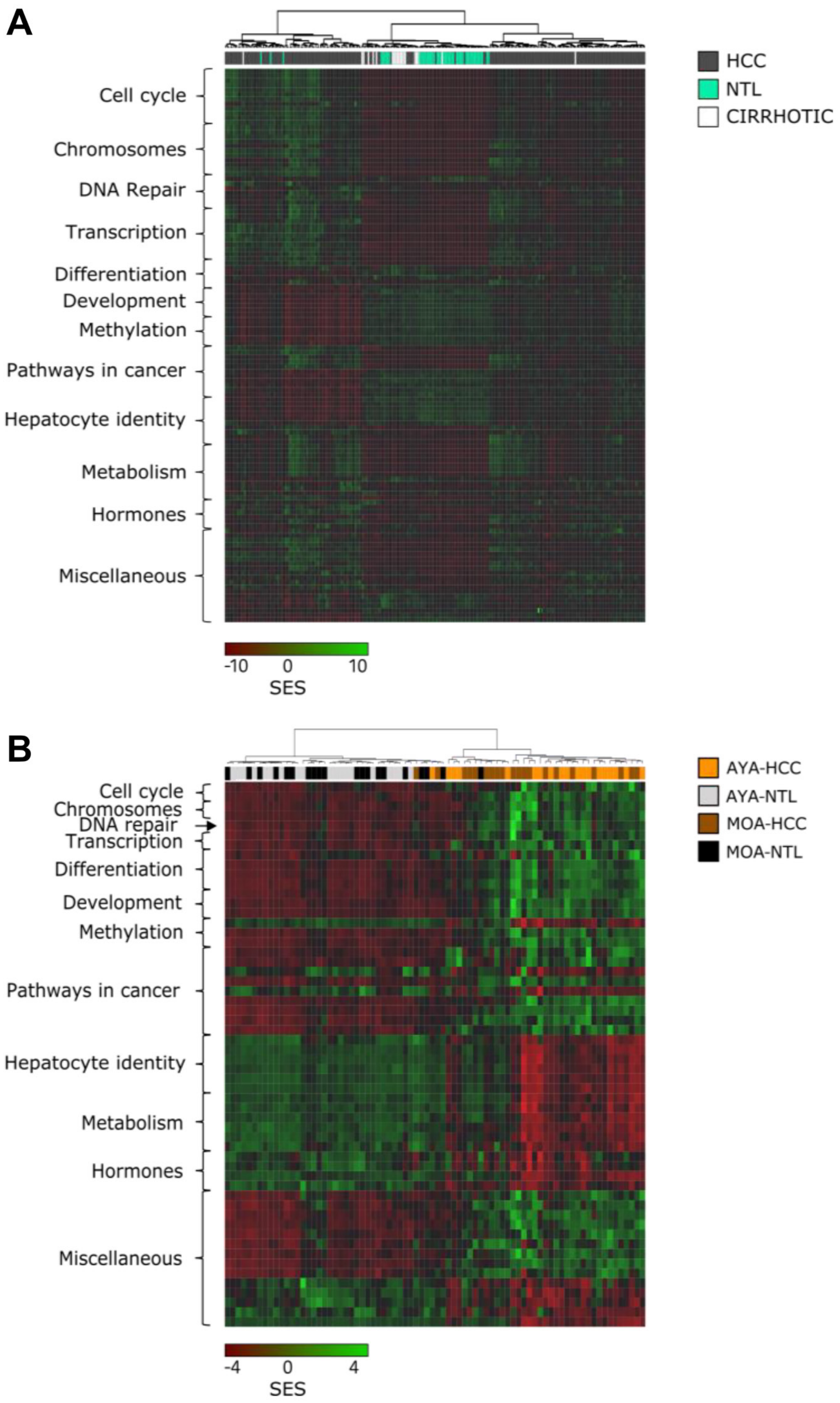

Supplementary Figure 4: Heatmap-based unsupervised hierarchical clustering of HCC/NTLs from different origins, produced from SES of significant gene sets. (**A**) Heatmap-based unsupervised hierarchical clustering of HCCs (n = 195) (dark grey) and non-cirrhotic (n = 47) (green) and cirrhotic (n = 20) (white) NTLs (top dendrogram) in non-Amerind, French (GSE62232) (n = 81), Taiwanese (GSE45436) (n = 72), and Turkish (GSE17548) (n = 17) patients, produced from SES of the 118 gene sets [6–8]. (**B**) Heatmap-based unsupervised hierarchical clustering of HCCs and NTLs (top dendrogram) in Peruvian AYA (n = 19) and MOA (n = 20) patients, produced from SES of the 56 Peruvian HCC-specific gene sets. Black: MOA-NTL; Brown: MOA-HCC; Grey: AYA-NTL; Orange: AYA-HCC.

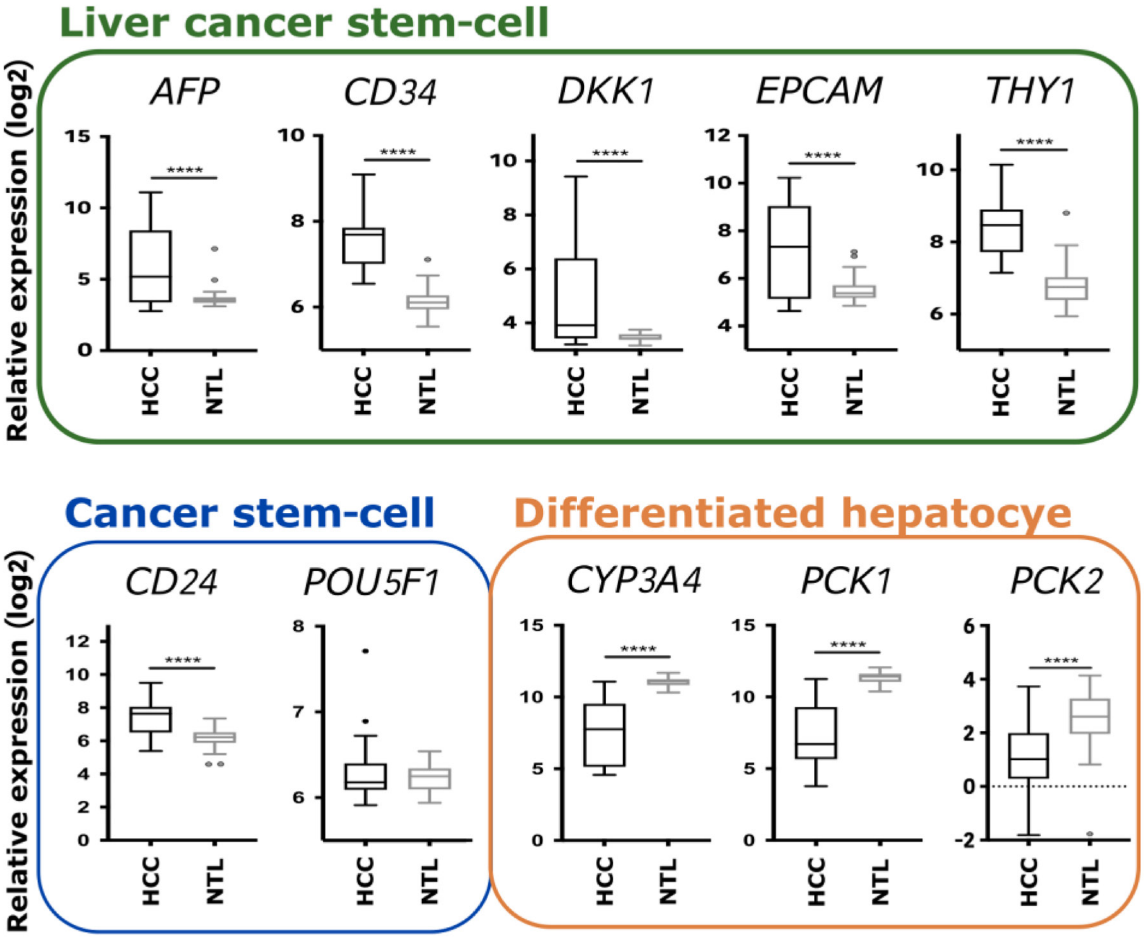

Supplementary Figure 5: Expression of some marker genes in Peruvian HCC. Box-and-whisker plots representing gene expression (log2) in Peruvian HCC/NTLs ($n = 39$) of *AFP*, *CD34*, *CYP3A4*, *DKK1*, *EPCAM*, and *THY1* liver cancer stem-cell gene markers (upper panel); CD24 and POU5F1 cancer stem-cell gene markers (left – lower panel); and PCK1 and PCK2 differentiated hepatocyte gene markers (right – lower panel), as measured by gene expression microarray (Affymetrix Human Genome U133 Plus 2.0 array). $^{****}p < 0.0001$.

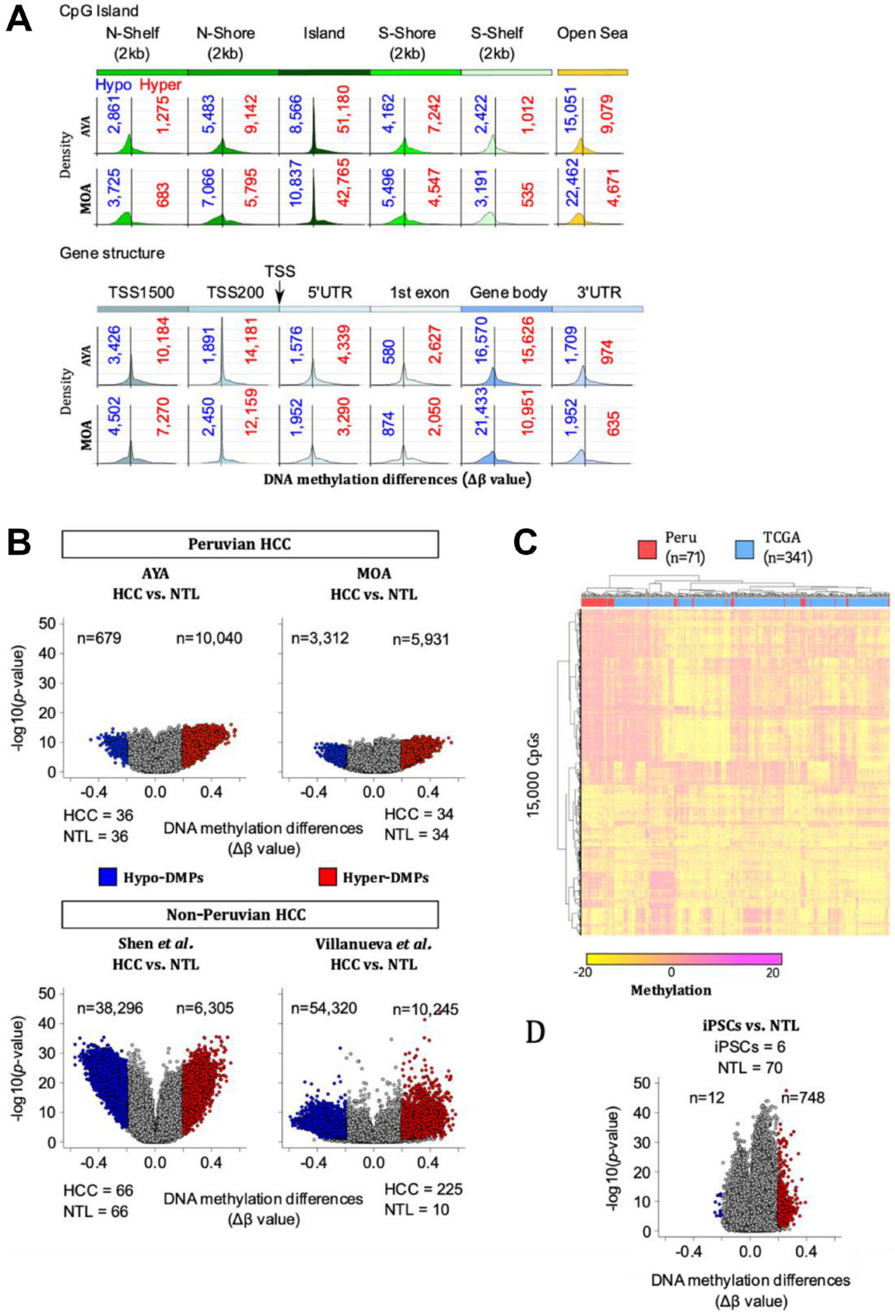

Supplementary Figure 6: Comparative profiling of DNA methylation dynamics in HCC and relevant biological model. (**A**) Schematic view of the number of hyper- and hypo-DMPs within CpG island structure (upper panel) and gene structure (lower panel). Density plots show the frequency distribution of DMP differences between hyper- and hypo-methylated 5mCs for the given genomic region. Blue: hypomethylated DMPs; Red: hypermethylated DMPs. (**B**) Volcano plots displaying HCC/NTL DNA methylation differences in Peruvian AYA and MOA (upper panel) (GSE136319, GSE136380) and Asian (GSE37988) and European (GSE56588) patients (lower panel) [9, 10]. (**C**) Heatmap-based unsupervised hierarchical clustering of HCCs from different origins (top dendogram), produced from β values of cancer-specifically hypermethylated CpGs (n = 15,000) (left dendogram), as previously described in [11]. Blue: data from The Cancer Genome Atlas - Liver Hepatocellular Carcinoma (TCGA-LIHC); Red: Peruvian specimen data. (**D**) Volcano plot displaying Peruvian NTL vs. induced pluripotent stem cells (iPSCs) (GSE141521) DNA methylation differences [12]. (B,D) DMPs were identified by Δβ score ≥ |0.2| and adjusted *p-value* < 0.05. Blue: hypomethylated DMPs; Red: hypermethylated DMPs.

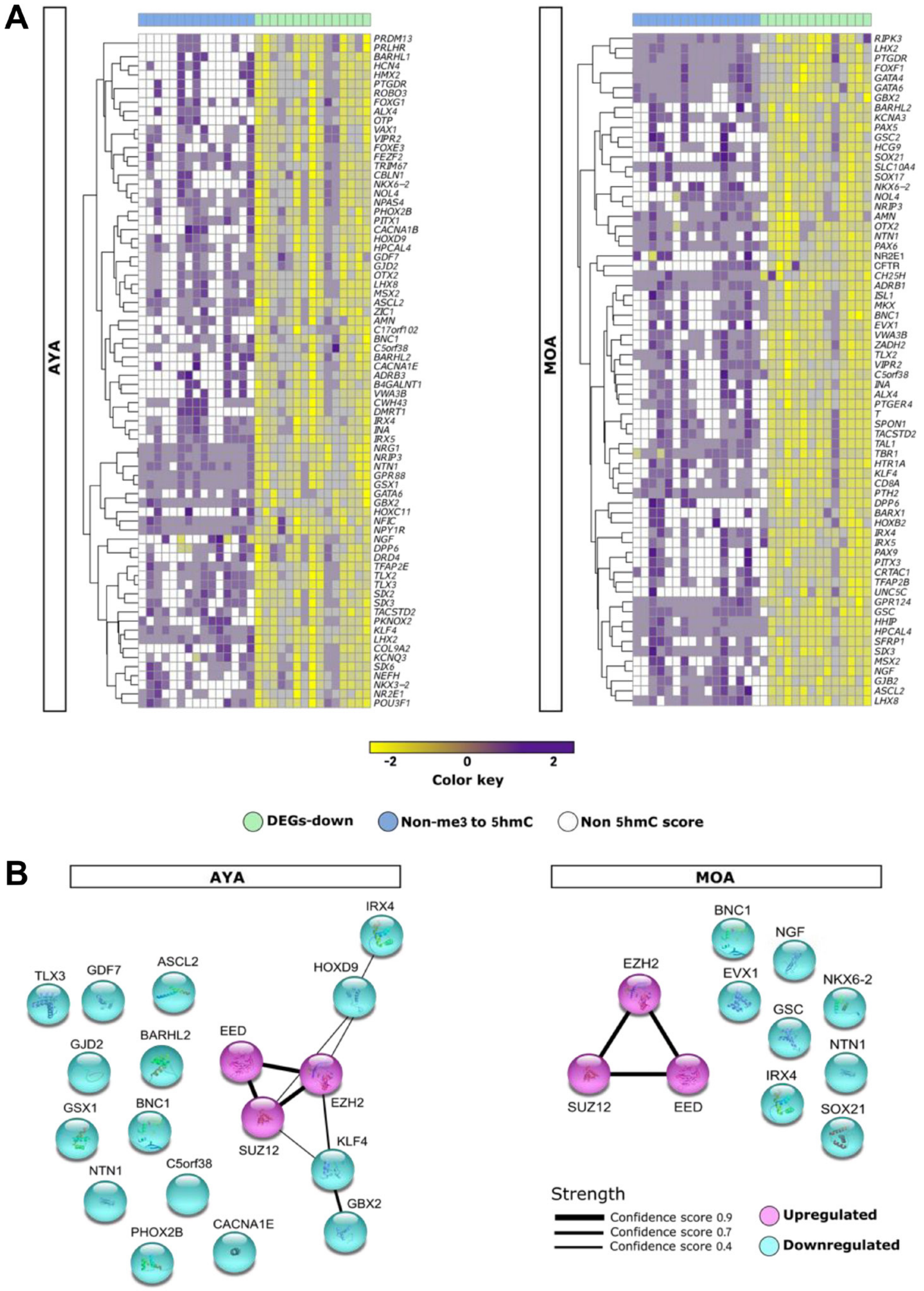

Supplementary Figure 7: Integrative analysis of DNA hydroxymethylation and gene expression data. (**A**) Heatmap of the integrative status of PRC2 targets in AYA (left panel) and MOA (right panel). The color key shows levels of expression (green) and hydroxymethylation (blue) independently scaled between -2 to 2. Absence of 5hmC cue at focal position is represented in white in the heatmap. (**B**) Mapping of the protein–protein interaction network for PRC2 targets displaying high correlation (Spearman $\rho > 0.5$) between gene expression and DNA hydroxymethylation in AYA (left panel) and MOA (right panel). Edges represent protein–protein associations meant to be specific and meaningful and line thickness indicates the strength of data support, with edge confidence scores ranging from 0.7 (thinner lines) to 0.9 (thicker lines). Nodes are filled with known or predicted 3D structure. Blue: downregulated genes; Purple: upregulated genes.

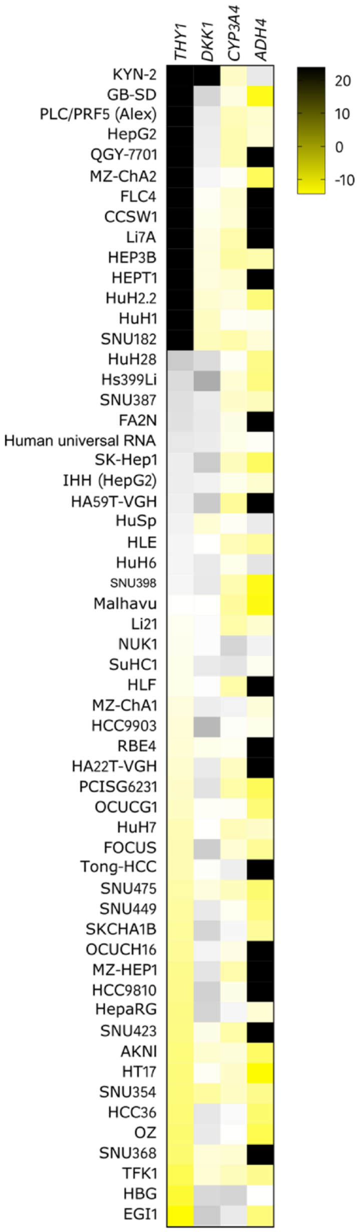

Supplementary Figure 8: Heatmap representation of the relative expression (mean of triplicate experiments) of ADH4, CYP3A4, DKK1, and THY1 progenitor-like gene markers as measured in 57 different HCC cell lines measured by qPCR [13].

Supplementary Table 1: Results of HCC/NTL SES analysis in AYA and MOA patients. See Supplementary Table 1

Supplementary Table 2: Baseline clinicodemographic features of the Peruvian and non-Amerind HCC patients scrutinized using Gene Expression Omnibus (GEO) HCC/NTL datasets from Affymetrix Human Genome U133 Plus 2.0 array. See Supplementary Table 2

Supplementary Table 3: Results of HCC/NTL SES analysis in non-Amerind patients. See Supplementary Table 3

Supplementary Table 4: Data on the 961 HCC/NTL-differentially expressed genes constituent of the Amerind signature. See Supplementary Table 4

Supplementary Table 5: Baseline demographical and clinical features of the Peruvian HCC patients scrutinized using qPCR assays

	Overall	AYA	MOA	Statistical significance
	(n = 65)	(n = 40)	(n = 25)	
**Haplogroup**				
A	9 (14.5%)	7 (17.9%)	2 (8.7%)	$p > 0.05$
B	30 (48.5%)	18 (46.2%)	12 (52.2%)	(χ^2 test)
C	14 (22.5%)	10 (25.6%)	4 (17.4%)	
D	9 (14.5%)	4 (10.3%)	5 (21.7%)	
**Age (years)**				
Mean ± SD	41 ± 21.02	25.8 ± 7.2	64.7 ± 11.1	$p < 0.0001$
Median	32	25.5	65	(t-test)
Range	[13–83]	[13–44]	[46–83]	
**Gender**				
Male	44 (67.7%)	24 (60%)	20 (80%)	$p > 0.05$
Female	21 (32.3%)	16 (40%)	5 (20%)	(χ^2 test)
**A-fetoprotein, mg/ml**				
Mean ± SD	7.56E+04 ± 1,49E+05	8.76E+04 ± 1.56E+05	5.86E+04 ± 1.40E+05	$p > 0.05$
Median	2.85E+3	5.51E+3	4.88E+2	(t-test)
Range	[0.6–6.38E+05]	[0.6–6.38E+05]	[1–5.46E+05]	
**Tumour size (cm)**				
Mean ± SD	14.5 ± 8.8	15.5 ± 9.2	13.4 ± 8.1	$p > 0.05$
Range	[4–30]	[5–30]	[4–30]	(t-test)
**Tumour primary pattern**				
Solid	3 (7.7%)	2 (8.7%)	1 (6.2%)	$p > 0.05$
Trabecular	36 (92.3%)	21 (91.3%)	15 (93.8%)	(χ^2 test)
**Tumour differentiation**				
Moderately	46 (79.3%)	29 (80.6%)	17 (77.3%)	$p > 0.05$
Poorly	12 (20.7%)	7 (19.4%)	5 (22.7%)	(χ^2 test)
**Cirrhosis**				
Positive	0 (0%)	0 (0%)	0 (0%)	Not applicable
Negative	32 (100%)	17 (100%)	15 (100%)	
**HBV infection**				
Positive	53 (81.5%)	35 (87.5%)	18 (72%)	$p > 0.05$
Negative	12 (18.5%)	5 (12.5%)	7 (28%)	(χ^2 test)

Supplementary Table 6: Baseline demographical features of the Peruvian HCC patients scrutinized for serum retinol level

Feature	Overall (n = 47)	AYA (n = 26)	MOA (n = 21)	Statistical significance
**Age (years)**				$p < 0.0001$ (t-test)
Mean ± SD	44.1 ± 20.7	27.9 ± 7.8	64.1 ± 11.9	
Median	37	28	63	
Range	[12–84]	[12–40]	[46–84]	
Interquartile range	37	13.7	18.5	
**Gender**				$p > 0.05$ (χ^2 test)
Female	18 (38.3%)	12 (46.2%)	6 (28.6%)	
Male	29 (61.7%)	14 (53.8%)	15 (71.4%)	
**Retinol (µg/dL)**				$p > 0.05$ (t-test)
Mean ± SD	28.1 ± 19.6	27 ± 20.2	28.8 ± 19.3	
Median	26	26	21.5	
Range	[2–72]	[2–68]	[4–72]	
Interquartile range	26	33	26.7	

Blood samples were collected between August 2018 and February 2020 and stored at –80°C prior analysis, 17 months in average. Analysis of variance (ANOVA) did not reveal any significant difference of retinol level in function of the storage time ($p > 0.05$). Deficiency was considered for serum retinol levels < 30 µg/dL and severe deficiency < 10 µg/dL, according to the WHO Vitamin and Mineral Nutrition Information System (VMNIS) (WHO/NMH/NHD/MNM/11.3) [14].

Supplementary Table 7: References of DNA primers for PCR assays and antibodies for IHC

**Commercial PCR assays**			
**Gene symbol (HGNC)**	**Catalogue number**	**Provider**	
AFP	QT00085183	Qiagen QuantiTect Primer Assay primers	
THY1	QT00023569	Qiagen QuantiTect Primer Assay primers	
EPCAM	QT00000371	Qiagen QuantiTect Primer Assay primers	
PCK1	QT00001197	Qiagen QuantiTect Primer Assay primers	
ADH1B	QT01160635	Qiagen QuantiTect Primer Assay primers	
ADH4	QT00000784	Qiagen QuantiTect Primer Assay primers	
ESR1	QT00044492	Qiagen QuantiTect Primer Assay primers	
CD34	qHSACID0007456	BioRad	
IGF1R	qHSACID0015667	BioRad	
KPNA2	qHSACID0012953	BioRad	
**Homemade PCR assays**			
**Gene symbol (HGNC) or name***	**Product size (bp)**	**Reverse primer (5′→3′)**	**Sense primer (5′→3′)**
mt16023*	-	GTTCTTTCATGGGGAAGCA	-
mt16422*	-	ATTGATTTCACGGAGGATGG	-
CYP3A4	99	GGATGAAAGAAAGTCGCCTCG	TGTGGGACTCAGTTTCTTTTGA
CD24	119	GGCACTGCTCCTACCCAC	TGGTGGTGGCATTAGTTGGA
POU5F1	85	GGTGGAGGAAGCTGACAACA	GGTTCGCTTTCTCTTTCGGG
PCK2	102	GCTGGAAAGTGGAGTGTGTG	CAACCCCAAAGAAGCCGTTC
ADH1A	92	TCTGTCTCATTGGCTGTGGA	GCCAAACACAGCACAGGTAG
ADH1C	81	CGTTTGAAGTCATCGGTCGG	CAATGACACTTGTGCCACATG
RARB	87	TCACAGATCTCCGTAGCATCA	TGAGAGGTGGCATTGATCCA
AURKA	84	GCATCATGGACCGATCTAAAGA	CGAGAACACGTTTTGGACCT
CTNNB1	90	TGGACTTGATATTGGTGCCCA	TGGCCATATCCACCAGAGT
DKK1	157	CCTGAGGCACAGTCTGATGA	TCCGAGGAGAAATTGAGGAA
**IHC tag antibodies**			
**Protein name**	**Catalogue number**	**Provider**	
AFP	NBP1-90233	Abcam	
CYP3A4	NBP2-37502SS	Novusbio	
EPCAM	14-9526	eBiosciences	
PCK2	NBP2-33685	Novusbio	
POU5F1	NBP2-55475	Novusbio	
THY1	NBP2-52961	Novusbio	

SUPPLEMENTARY REFERENCES

1. Tosolini M, Algans C, Pont F, Ycart B, Fournié JJ. Large-scale microarray profiling reveals four stages of immune escape in non-Hodgkin lymphomas. Oncoimmunology. 2016; 5:e1188246. https://doi.org/10.1080/2162402X.2016.1188246. [PubMed]
2. Yoshihara K, Shahmoradgoli M, Martínez E, Vegesna R, Kim H, Torres-Garcia W, Treviño V, Shen H, Laird PW, Levine DA, Carter SL, Getz G, Stemke-Hale K, et al. Inferring tumour purity and stromal and immune cell admixture from expression data. Nat Commun. 2013; 4:2612. https://doi.org/10.1038/ncomms3612. [PubMed]
3. Sia D, Jiao Y, Martinez-Quetglas I, Kuchuk O, Villacorta-Martin C, Castro de Moura M, Putra J, Camprecios G, Bassaganyas L, Akers N, Losic B, Waxman S, Thung SN, et al. Identification of an immune-specific class of hepatocellular carcinoma, based on molecular features. Gastroenterology. 2017; 153:812–26. https://doi.org/10.1053/j.gastro.2017.06.007. [PubMed]
4. Okrah K, Tarighat S, Liu B, Koeppen H, Wagle MC, Cheng G, Sun C, Dey A, Chang MT, Sumiyoshi T, Mounir Z, Cummings C, Hampton G, et al. Transcriptomic analysis of hepatocellular carcinoma reveals molecular features of disease progression and tumor immune biology. NPJ Precis Oncol. 2018; 2:25. https://doi.org/10.1038/s41698-018-0068-8. [PubMed]
5. Tosolini M, Pont F, Poupot M, Vergez F, Nicolau-Travers ML, Vermijlen D, Sarry JE, Dieli F, Fournié JJ. Assessment of tumor-infiltrating TCRVγ9Vδ2 γδ lymphocyte abundance by deconvolution of human cancers microarrays. Oncoimmunology. 2017; 6:e1284723. https://doi.org/10.1080/2162402X.2017.1284723. [PubMed]
6. Yildiz G, Arslan-Ergul A, Bagislar S, Konu O, Yuzugullu H, Gursoy-Yuzugullu O, Ozturk N, Ozen C, Ozdag H, Erdal E, Karademir S, Sagol O, Mizrak D, et al. Genome-wide transcriptional reorganization associated with senescence-to-immortality switch during human hepatocellular carcinogenesis. PLoS One. 2013; 8:e64016. https://doi.org/10.1371/journal.pone.0064016. [PubMed]
7. Schulze K, Imbeaud S, Letouzé E, Alexandrov LB, Calderaro J, Rebouissou S, Couchy G, Meiller C, Shinde J, Soysouvanh F, Calatayud AL, Pinyol R, Pelletier L, et al. Exome sequencing of hepatocellular carcinomas identifies new mutational signatures and potential therapeutic targets. Nat Genet. 2015; 47:505–11. https://doi.org/10.1038/ng.3252. [PubMed]
8. Wang HW, Hsieh TH, Huang SY, Chau GY, Tung CY, Su CW, Wu JC. Forfeited hepatogenesis program and increased embryonic stem cell traits in young hepatocellular carcinoma (HCC) comparing to elderly HCC. BMC Genomics. 2013; 14:736. https://doi.org/10.1186/1471-2164-14-736. [PubMed]
9. Shen J, Wang S, Zhang YJ, Kappil M, Wu HC, Kibriya MG, Wang Q, Jasmine F, Ahsan H, Lee PH, Yu MW, Chen CJ, Santella RM. Genome-wide DNA methylation profiles in hepatocellular carcinoma. Hepatology. 2012; 55:1799–808. https://doi.org/10.1002/hep.25569. [PubMed]
10. Villanueva A, Portela A, Sayols S, Battiston C, Hoshida Y, Méndez-González J, Imbeaud S, Letouzé E, Hernandez-Gea V, Cornella H, Pinyol R, Solé M, Fuster J, et al. DNA methylation-based prognosis and epidrivers in hepatocellular carcinoma. Hepatology. 2015; 61:1945–56. https://doi.org/10.1002/hep.27732. [PubMed]
11. Cancer Genome Atlas Research Network. Comprehensive and integrative genomic characterization of hepatocellular carcinoma. Cell. 2017; 169:1327–41. https://doi.org/10.1016/j.cell.2017.05.046. [PubMed]
12. Nishino K, Takasawa K, Okamura K, Arai Y, Sekiya A, Akutsu H, Umezawa A. Identification of an epigenetic signature in human induced pluripotent stem cells using a linear machine learning model. Hum Cell. 2021; 34:99–110. https://doi.org/10.1007/s13577-020-00446-3. [PubMed]
13. Pineau P, Marchio A, Nagamori S, Seki S, Tiollais P, Dejean A. Homozygous deletion scanning in hepatobiliary tumor cell lines reveals alternative pathways for liver carcinogenesis. Hepatology. 2003; 37:852–61. https://doi.org/10.1053/jhep.2003.50138. [PubMed]
14. World Health Organization. Global prevalence of vitamin A deficiency in populations at risk 1995–2005: WHO Global Database on Vitamin A Deficiency. World Health Organization. Geneva; 2009.
