# Supplementary material for: Global DNA hypermethylation pattern and unique gene expression signature in liver cancer from patients with Indigenous American ancestry
Source: Oncotarget. 2021 Mar 2;12(5):475–92. doi: 10.18632/oncotarget.27890 (PMC7939527; doi:10.18632/oncotarget.27890)
Supplement: Supplementary file 2 [file oncotarget-12-475-s002.docx]

**Supplementary Table 1: Results of HCC/NTL SES analysis in AYA and MOA patients**

| **Patient group** | **Gene set class** | **Gene set** | **Database ID** | **Database** | **Collection** | **HCC SES mean** | **NTL SES mean** | **Fold change** | ***p*-value** |
| --- | --- | --- | --- | --- | --- | --- | --- | --- | --- |
| AYA | Cell cycle | Activation of the pre-replicative complex | R-HSA-68962 | Reactome | - | 8.2 | 2.1 | 4 | 1.6E-05 |
| AYA | Cell cycle | Activation of ATR in response to replication stress | R-HSA-176187 | Reactome | - | 6 | 1.2 | 5 | 1.2E-05 |
| AYA | Cell cycle | Activation of E2F1 target genes at G1S | R-HSA-539107 | Reactome | - | 9.5 | 4 | 2.4 | 7.5E-06 |
| AYA | Cell cycle | DNA strand elongation | R-HSA-69190 | Reactome | - | 14.5 | 4.9 | 2.9 | 3.0E-06 |
| AYA | Cell cycle | KEGG DNA replication | HSA03030 | KEGG | - | 16.4 | 7.8 | 2.1 | 3.0E-06 |
| AYA | Cell cycle | TP53 regulates transcription of genes involved in G1 cell cycle arrest | R-HSA-6804116 | Reactome | - | 11.6 | 6.8 | 1.7 | 2.3E-06 |
| AYA | Cell cycle | Unwinding of DNA | R-HSA-176974 | Reactome | - | 15.7 | 5.2 | 3 | 1.6E-06 |
| AYA | Cell cycle | MET activates PTK2 signalling | R-HSA-8874081 | Reactome | - | 24.7 | 14.1 | 1.8 | 6.9E-07 |
| AYA | Cell cycle | KEGG cell cycle | HSA04110 | KEGG | - | 13.9 | 7.5 | 1.9 | 3.2E-07 |
| AYA | Cell cycle | DNA replication | R-HSA-69306 | Reactome | - | 21.8 | 14.2 | 1.5 | 2.8E-07 |
| AYA | Cell cycle | TP53 regulates transcription of cell cycle genes | R-HSA-6791312 | Reactome | - | 21.8 | 13.9 | 1.6 | 2.7E-07 |
| AYA | Cell cycle | GO MCM complex | GO:0042555 | Gene Ontology | Cellular component | 24.1 | 10.4 | 2.3 | 1.7E-07 |
| AYA | Chromosome | GO centriole | GO:0005814 | Gene Ontology | Cellular component | 6.4 | 3.7 | 1.7 | 2.5E-05 |
| AYA | Chromosome | GO microtubule organizing centre part | GO:0005815 | Gene Ontology | Cellular component | 8.4 | 5.7 | 1.5 | 2.1E-05 |
| AYA | Chromosome | GO condensed chromosome centromeric region | GO:0000780 | Gene Ontology | Cellular component | 8.3 | 2.7 | 3.1 | 8.4E-06 |
| AYA | Chromosome | Chromosome maintenance | GO:0000780 | Gene Ontology | Cellular component | 7.6 | 2.4 | 3.2 | 5.1E-06 |
| AYA | Chromosome | Condensation of prophase chromosomes | R-HSA-73886 | Reactome | - | 10 | 3 | 3.3 | 3.5E-06 |
| AYA | Chromosome | GO condensed chromosome | GO:0000793 | Gene Ontology | Cellular component | 6.1 | 1.9 | 3.2 | 2.7E-06 |
| AYA | Chromosome | GO chromosome centromeric region | GO:0000775 | Gene Ontology | Cellular component | 11.6 | 5.3 | 2.2 | 2.4E-06 |
| AYA | Chromosome | Packaging of telomere ends | R-HSA-171306 | Reactome | - | 10.4 | 3.4 | 3.1 | 1.7E-06 |
| AYA | Chromosome | DNA damage/telomere stress induced senescence | R-HSA-2559586 | Reactome | - | 10.8 | 4.2 | 2.5 | 3.6E-07 |
| AYA | Chromosome | GO chromosome | GO:0005694 | Gene Ontology | Cellular component | 28.2 | 18.1 | 1.6 | 2.7E-07 |
| AYA | Chromosome | GO chromosome telomeric region | GO:0098687 | Gene Ontology | Cellular component | 19.8 | 12.9 | 1.5 | 1.1E-07 |
| AYA | Differentiation | GO main axon | GO:0030426 | Gene Ontology | Cellular component | 3.6 | 5.5 | 0.6 | 3.9E-05 |
| AYA | Differentiation | GO synaptonemal complex | GO:0007130 | Gene Ontology | Cellular component | 0.7 | 0.4 | 1.8 | 1.5E-05 |
| AYA | Differentiation | RUNX3 regulates YAP1-mediated transcription | R-HSA-8951671 | Reactome | - | 47.7 | 44 | 1.1 | 1.4E-05 |
| AYA | Differentiation | RUNX1 regulates genes involved in megakaryocyte differentiation and platelet function | R-HSA-8936459 | Reactome | - | 19.4 | 13.1 | 1.5 | 6.7E-07 |
| AYA | Differentiation | Activation of *HOX* genes during differentiation | R-HSA-5619507 | Reactome | - | 15.1 | 9 | 1.7 | 1.6E-07 |
| AYA | Differentiation | RUNX1 regulates transcription of genes involved in differentiation of HSCs | R-HSA-8939236 | Reactome | - | 25.9 | 19.6 | 1.3 | 4.9E-08 |
| AYA | Development | Meiotic recombination | R-HSA-912446 | Reactome | - | 3.7 | 0.6 | 5.8 | 7.6E-06 |
| AYA | Development | Reproduction | R-HSA-1474165 | Reactome | - | 1.3 | 0.3 | 5 | 2.2E-06 |
| AYA | Development | Meiosis | R-HSA-1500620 | Reactome | - | 4.2 | 1.2 | 3.4 | 1.7E-06 |
| AYA | Development | Activation of anterior *HOX* genes in hindbrain development during early embryogenesis | R-HSA-5617472 | Reactome | - | 15.1 | 9 | 1.7 | 1.6E-07 |
| AYA | Development | GO male germ cell nucleus | GO:0001673 | Gene Ontology | Cellular component | 4.7 | 2.8 | 1.7 | 1.2E-07 |
| AYA | Development | GO germ cell nucleus | GO:0043073 | Gene Ontology | Cellular component | 5.4 | 2.9 | 1.9 | 5.6E-09 |
| AYA | DNA repair | HDR through single strand annealing (SSA) | R-HSA-5685938 | Reactome | - | 6.8 | 2.3 | 3 | 7.7E-06 |
| AYA | DNA repair | GO mismatch repair complex | GO:0032300 | Gene Ontology | Cellular component | 24.5 | 15.9 | 1.5 | 4.0E-06 |
| AYA | DNA repair | Hallmark DNA repair | M5898 | MsigDB | Hallmark | 25.7 | 21.4 | 1.2 | 1.4E-06 |
| AYA | DNA repair | DNA repair | R-HSA-73894 | Reactome | - | 16.7 | 10.6 | 1.6 | 5.5E-07 |
| AYA | DNA repair | Recruitment and ATM-mediated phosphorylation of repair and signalling proteins at DNA double strand breaks | R-HSA-5693565 | Reactome | - | 15.9 | 9.7 | 1.6 | 4.5E-07 |
| AYA | DNA repair | HDR through MMEJ (alt-NHEJ) | R-HSA-5685939 | Reactome | - | 25.6 | 16.2 | 1.6 | 3.3E-07 |
| AYA | DNA repair | DNA double strand break response | R-HSA-5693606 | Reactome | - | 16.5 | 9.8 | 1.7 | 1.9E-07 |
| AYA | Hormones | Hallmark oestrogen response late | M5907 | MsigDB | Hallmark | 17.6 | 19.1 | 0.9 | 2.4E-03 |
| AYA | Hormones | Hallmark oestrogen response early | M5906 | MsigDB | Hallmark | 18.9 | 21.4 | 0.9 | 1.5E-04 |
| AYA | Hormones | Amine-derived hormones | R-HSA-209776 | Reactome | - | 0.8 | 1.8 | 0.5 | 3.4E-06 |
| AYA | Hormones | Activated PKN1 stimulates transcription of AR (androgen receptor) regulated genes *KLK2* and *KLK3* | R-HSA-5625886 | Reactome | - | 11.2 | 3.7 | 3 | 5.0E-07 |
| AYA | Hormones | KEGG steroid hormone biosynthesis | HSA00140 | KEGG | - | 14.6 | 34.4 | 0.4 | 3.5E-07 |
| AYA | Hormones | Growth hormone receptor signalling | R-HSA-982772 | Reactome | - | 20.6 | 27.2 | 0.8 | 5.8E-11 |
| AYA | Loss of hepatocytes feat. | Branched-chain amino acid catabolism | R-HSA-70895 | Reactome | - | 31.4 | 45.8 | 0.7 | 4.1E-07 |
| AYA | Loss of hepatocytes feat. | KEGG metabolism of xenobiotics by cytochrome P450 | HSA00980 | KEGG | - | 21.4 | 46.4 | 0.5 | 2.0E-07 |
| AYA | Loss of hepatocytes feat. | Hallmark xenobiotic metabolism | M5934 | MsigDB | Hallmark | 35.5 | 52 | 0.7 | 8.3E-08 |
| AYA | Loss of hepatocytes feat. | Fatty acids | R-HSA-211935 | Reactome | - | 24.7 | 50.9 | 0.5 | 6.7E-08 |
| AYA | Loss of hepatocytes feat. | Hallmark fatty acid metabolism | M5935 | MsigDB | Hallmark | 33.2 | 43.9 | 0.8 | 6.1E-08 |
| AYA | Loss of hepatocytes feat. | Hallmark bile acid metabolism | M5948 | MsigDB | Hallmark | 23.1 | 37.8 | 0.6 | 4.9E-08 |
| AYA | Loss of hepatocytes feat. | KEGG drug metabolism cytochrome P450 | HSA00982 | KEGG | - | 21.2 | 49.7 | 0.4 | 4.1E-08 |
| AYA | Loss of hepatocytes feat. | KEGG drug metabolism other enzymes | HSA00983 | KEGG | - | 22.4 | 53.2 | 0.4 | 1.7E-08 |
| AYA | Loss of hepatocytes feat. | Synthesis of (16-20)-hydroxyeicosatetraenoic acids (HETE) | R-HSA-2142816 | Reactome | - | 25.6 | 62.4 | 0.4 | 1.5E-08 |
| AYA | Loss of hepatocytes feat. | KEGG primary bile acid biosynthesis | HSA00120 | KEGG | - | 24.6 | 53.4 | 0.5 | 2.1E-09 |
| AYA | Methylation | Methylation | R-HSA-156581 | Reactome | - | 39.4 | 52.4 | 0.8 | 6.6E-06 |
| AYA | Methylation | GO methylosome | GO:0034709 | Gene Ontology | Cellular component | 30.7 | 20.3 | 1.5 | 1.5E-06 |
| AYA | Methylation | GO ESC E Z complex | GO:0035098 | Gene Ontology | Cellular component | 30.6 | 19.5 | 1.6 | 9.4E-07 |
| AYA | Methylation | DNA methylation | R-HSA-5334118 | Reactome | - | 10.5 | 2.9 | 3.6 | 9.1E-07 |
| AYA | Methylation | PRC2 methylates histones and DNA | R-HSA-212300 | Reactome | - | 13.4 | 4.4 | 3 | 5.8E-07 |
| AYA | Methylation | RMTs methylate histone arginines | R-HSA-3214858 | Reactome | - | 30.4 | 19.9 | 1.5 | 8.0E-09 |
| AYA | Metabolism | KEGG butanoate metabolism | HSA00650 | KEGG | - | 25.4 | 35.3 | 0.7 | 6.7E-06 |
| AYA | Metabolism | Signalling by Retinoic Acid | R-HSA-5362517 | Reactome | - | 19.3 | 25.1 | 0.8 | 2.8E-07 |
| AYA | Metabolism | KEGG arachidonic acid metabolism | HSA00590 | KEGG | - | 11.2 | 19.4 | 0.6 | 2.4E-07 |
| AYA | Metabolism | KEGG starch and sucrose metabolism | HSA00500 | KEGG | - | 13.2 | 29.3 | 0.5 | 1.7E-07 |
| AYA | Metabolism | KEGG glycine serine and threonine metabolism | HSA00260 | KEGG | - | 36.2 | 62.3 | 0.6 | 4.8E-08 |
| AYA | Metabolism | KEGG folate biosynthesis | HSA00790 | KEGG | - | 28.9 | 44.7 | 0.6 | 4.0E-08 |
| AYA | Metabolism | KEGG linoleic acid metabolism | HSA00591 | KEGG | - | 12 | 25.2 | 0.5 | 3.5E-08 |
| AYA | Metabolism | KEGG β alanine metabolism | HSA00410 | KEGG | - | 30.1 | 47.2 | 0.6 | 2.6E-08 |
| AYA | Metabolism | KEGG retinol metabolism | HSA00830 | KEGG | - | 16.9 | 43.3 | 0.4 | 1.9E-08 |
| AYA | Metabolism | KEGG tryptophan metabolism | HSA00380 | KEGG | - | 20.4 | 38.2 | 0.5 | 4.3E-09 |
| AYA | Metabolism | KEGG histidine metabolism | HSA00340 | KEGG | - | 23.9 | 38.3 | 0.6 | 1.7E-10 |
| AYA | Metabolism | KEGG sulphur metabolism | HSA00920 | KEGG | - | 29.9 | 40.4 | 0.7 | 2.5E-12 |
| AYA | Transcription | KEGG basal transcription factors | HSA03022 | KEGG | - | 12 | 8.1 | 1.5 | 1.4E-05 |
| AYA | Transcription | GO saga complex | GO:0000124 | Gene Ontology | Cellular component | 28.7 | 18.3 | 1.6 | 2.4E-06 |
| AYA | Transcription | RNA polymerase I promoter opening | R-HSA-73728 | Reactome | - | 14.4 | 4.8 | 3 | 5.7E-07 |
| AYA | Transcription | RNA polymerase I chain elongation | R-HSA-73777 | Reactome | - | 14.5 | 6.1 | 2.4 | 1.6E-07 |
| AYA | Transcription | NoRC negatively regulates rRNA expression | R-HSA-427413 | Reactome | - | 17.5 | 8.7 | 2 | 9.2E-08 |
| AYA | Transcription | HDACs deacetylate histones | R-HSA-3214815 | Reactome | - | 20.1 | 10 | 2 | 8.3E-08 |
| AYA | Transcription | HATs acetylate histones | R-HSA-3214847 | Reactome | - | 23.9 | 14.8 | 1.6 | 6.6E-08 |
| AYA | Transcription | B-WICH complex positively regulates rRNA expression | R-HSA-5250924 | Reactome | - | 19.7 | 10.4 | 1.9 | 5.8E-08 |
| AYA | Transcription | RNA polymerase I promoter clearance | R-HSA-73854 | Reactome | - | 19.6 | 10.6 | 1.8 | 2.8E-08 |
| AYA | Transcription | RNA polymerase I transcription | R-HSA-73857 | Reactome | - | 20.1 | 11 | 1.8 | 2.3E-08 |
| AYA | Transcription | Positive epigenetic regulation of rRNA expression | R-HSA-5250913 | Reactome | - | 23.3 | 13.6 | 1.7 | 1.6E-08 |
| AYA | Pathways in cancer | PI3KAKT signalling in cancer | R-HSA-8853334 | Reactome | - | 8.7 | 10.3 | 0.8 | 1.4E-04 |
| AYA | Pathways in cancer | Signalling by Wnt in cancer | R-HSA-4791275 | Reactome | - | 31.5 | 25.9 | 1.2 | 7.1E-06 |
| AYA | Pathways in cancer | Hallmark Wnt β-catenin signalling | M5895 | MsigDB | Hallmark | 26 | 19.3 | 1.3 | 3.3E-06 |
| AYA | Pathways in cancer | Down-regulation of ERBB2:ERBB3 signalling | R-HSA-1358803 | Reactome | - | 45.6 | 51.6 | 0.9 | 1.6E-06 |
| AYA | Pathways in cancer | Hallmark MYC targets v1 | M5926 | MsigDB | Hallmark | 50.1 | 41.5 | 1.2 | 1.0E-06 |
| AYA | Pathways in cancer | GRB7 events in ERBB2 signalling | R-HSA-1306955 | Reactome | - | 33.7 | 46.5 | 0.7 | 9.6E-07 |
| AYA | Pathways in cancer | Deactivation of the β-catenin transactivating complex | R-HSA-3769402 | Reactome | - | 25.8 | 19.9 | 1.3 | 1.5E-07 |
| AYA | Pathways in cancer | Regulation of PTEN gene transcription | R-HSA-8943724 | Reactome | - | 30.4 | 24 | 1.3 | 1.2E-07 |
| AYA | Pathways in cancer | mTOR signalling | R-HSA-165159 | Reactome | - | 31.1 | 25.2 | 1.2 | 6.9E-08 |
| AYA | Pathways in cancer | Formation of the β-catenin:TCF transactivating complex | R-HSA-201722 | Reactome | - | 19.1 | 10.1 | 1.9 | 2.4E-08 |
| AYA | Pathways in cancer | SLIT2:ROBO1 increases RHOA activity | R-HSA-8985586 | Reactome | - | 48.1 | 24.1 | 2 | 1.8E-08 |
| AYA | Misc. - Up | KEGG systemic lupus erythematosus | HSA05322 | KEGG | - | 15.4 | 9.6 | 1.6 | 5.6E-06 |
| AYA | Misc. - Up | Hallmark protein secretion | M5910 | MsigDB | Hallmark | 40.3 | 34.1 | 1.2 | 3.1E-06 |
| AYA | Misc. - Up | Resolution of sister chromatid cohesion | R-HSA-2500257 | Reactome | - | 11.9 | 4.6 | 2.6 | 2.1E-06 |
| AYA | Misc. - Up | Export of viral ribonucleoproteins from nucleus | R-HSA-168274 | Reactome | - | 37.9 | 25 | 1.5 | 1.9E-06 |
| AYA | Misc. - Up | SIRT1 negatively regulates rRNA expression | R-HSA-427359 | Reactome | - | 11.5 | 3.5 | 3.3 | 7.4E-07 |
| AYA | Misc. - Up | AURKA activation by TPX2 | R-HSA-8854518 | Reactome | - | 16.4 | 9.6 | 1.7 | 5.2E-07 |
| AYA | Misc. - Up | SUMOylation | R-HSA-2990846 | Reactome | - | 29.1 | 20.4 | 1.4 | 1.4E-07 |
| AYA | Misc. - Up | Senescence-associated secretory phenotype (SASP) | R-HSA-2559582 | Reactome | - | 16.3 | 10 | 1.6 | 7.3E-08 |
| AYA | Misc. - Up | ERCC6 (CSB) and EHMT2 (G9a) positively regulate rRNA expression | R-HSA-427389 | Reactome | - | 16.7 | 7 | 2.4 | 7.1E-08 |
| AYA | Misc. - Up | KEGG small cell lung cancer | HSA05222 | KEGG | - | 20.6 | 14.5 | 1.4 | 5.3E-08 |
| AYA | Misc. - Up | Transferrin endocytosis and recycling | R-HSA-917977 | Reactome | - | 22.7 | 18.8 | 1.2 | 5.6E-09 |
| AYA | Misc. - Up | Formation of senescence-associated heterochromatin foci (SAHF) | R-HSA-2559584 | Reactome | - | 17.3 | 11.5 | 1.5 | 4.2E-09 |
| AYA | Misc. - Up | Activation of RAC1 | R-HSA-418856 | Reactome | - | 31.1 | 18.4 | 1.7 | 2.4E-09 |
| AYA | Misc. - Up | Iron uptake and transport | R-HSA-917937 | Reactome | - | 33.9 | 29.3 | 1.2 | 2.6E-12 |
| AYA | Misc. - Down | Interleukin-20 family signalling | R-HSA-8854691 | Reactome | - | 5.9 | 7.4 | 0.8 | 5.2E-06 |
| AYA | Misc. - Down | ZBP1(DAI) mediated induction of type I IFNs | R-HSA-1606322 | Reactome | - | 23.6 | 27.8 | 0.8 | 2.4E-06 |
| AYA | Misc. - Down | DExH-box helicases activate type I IFN and inflammatory cytokines production | R-HSA-3134963 | Reactome | - | 37.3 | 42.4 | 0.9 | 9.8E-07 |
| AYA | Misc. - Down | GO sodium channel complex | GO:0034706 | Gene Ontology | Cellular component | 0 | 0.1 | 0.4 | 8.0E-07 |
| AYA | Misc. - Down | KEGG ABC transporters | HSA02010 | KEGG | - | 10.4 | 18.4 | 0.6 | 8.6E-09 |
| AYA | Misc. - Down | Aflatoxin activation and detoxification | R-HSA-5423646 | Reactome | - | 28.9 | 49 | 0.6 | 2.4E-09 |
| MOA | Cell cycle | Activation of the pre-replicative complex | R-HSA-68962 | Reactome | - | 10.2 | 3.1 | 3.3 | 8.5E-05 |
| MOA | Cell cycle | Activation of ATR in response to replication stress | R-HSA-176187 | Reactome | - | 8 | 2 | 4.1 | 7.3E-05 |
| MOA | Cell cycle | Activation of E2F1 target genes at G1S | R-HSA-539107 | Reactome | - | 11.5 | 4.8 | 2.4 | 2.0E-05 |
| MOA | Cell cycle | DNA strand elongation | R-HSA-69190 | Reactome | - | 17.1 | 6.9 | 2.5 | 2.3E-05 |
| MOA | Cell cycle | KEGG DNA replication | HSA03030 | KEGG | - | 18.9 | 9.6 | 2 | 1.9E-05 |
| MOA | Cell cycle | TP53 regulates transcription of genes involved in G1 cell cycle arrest | R-HSA-6804116 | Reactome | - | 12.6 | 7.1 | 1.8 | 3.4E-04 |
| MOA | Cell cycle | Unwinding of DNA | R-HSA-176974 | Reactome | - | 18.6 | 8 | 2.3 | 3.1E-05 |
| MOA | Cell cycle | MET activates PTK2 signalling | R-HSA-8874081 | Reactome | - | 24.1 | 16.8 | 1.4 | 3.0E-03 |
| MOA | Cell cycle | KEGG cell cycle | HSA04110 | KEGG | - | 14.8 | 8.3 | 1.8 | 1.5E-06 |
| MOA | Cell cycle | DNA replication | R-HSA-69306 | Reactome | - | 24.1 | 15.6 | 1.5 | 1.4E-06 |
| MOA | Cell cycle | TP53 regulates transcription of cell cycle genes | R-HSA-6791312 | Reactome | - | 22.2 | 14.8 | 1.5 | 6.0E-06 |
| MOA | Cell cycle | GO MCM complex | GO:0042555 | Gene Ontology | Cellular component | 26.3 | 13.6 | 1.9 | 1.8E-05 |
| MOA | Chromosome | GO centriole | GO:0005814 | Gene Ontology | Cellular component | 7.1 | 4.1 | 1.7 | 3.7E-07 |
| MOA | Chromosome | GO microtubule organizing center part | GO:0005815 | Gene Ontology | Cellular component | 9.3 | 6.2 | 1.5 | 1.2E-06 |
| MOA | Chromosome | GO condensed chromosome centromeric region | GO:0000780 | Gene Ontology | Cellular component | 9.2 | 3 | 3 | 6.4E-06 |
| MOA | Chromosome | Chromosome ,aintenance | GO:0000780 | Gene Ontology | Cellular component | 8.7 | 3 | 2.9 | 2.7E-05 |
| MOA | Chromosome | Condensation of prophase chromosomes | R-HSA-73886 | Reactome | - | 10.7 | 3.6 | 3 | 1.5E-05 |
| MOA | Chromosome | GO condensed chromosome | GO:0000793 | Gene Ontology | Cellular component | 6.8 | 2.2 | 3.1 | 2.8E-06 |
| MOA | Chromosome | GO chromosome centromeric region | GO:0000775 | Gene Ontology | Cellular component | 12.4 | 5.8 | 2.1 | 1.8E-06 |
| MOA | Chromosome | Packaging of telomere ends | R-HSA-171306 | Reactome | - | 10.7 | 4.1 | 2.6 | 1.4E-05 |
| MOA | Chromosome | DNA damagetelomere stress induced senescence | R-HSA-2559586 | Reactome | - | 11.4 | 5 | 2.3 | 7.4E-06 |
| MOA | Chromosome | GO chromosome | GO:0005694 | Gene Ontology | Cellular component | 29.3 | 19.1 | 1.5 | 4.4E-07 |
| MOA | Chromosome | GO chromosome telomeric region | GO:0098687 | Gene Ontology | Cellular component | 20.4 | 13.6 | 1.5 | 1.1E-06 |
| MOA | Differentiation | GO main axon | GO:0030426 | Gene Ontology | Cellular component | 3.3 | 5.4 | 0.6 | 1.7E-08 |
| MOA | Differentiation | GO synaptonemal complex | GO:0007130 | Gene Ontology | Cellular component | 0.8 | 0.4 | 1.9 | 5.3E-04 |
| MOA | Differentiation | RUNX3 regulates YAP1-mediated transcription | R-HSA-8951671 | Reactome | - | 47.3 | 44.3 | 1.1 | 2.5E-03 |
| MOA | Differentiation | RUNX1 regulates genes involved in megakaryocyte differentiation and platelet function | R-HSA-8936459 | Reactome | - | 19.6 | 14 | 1.4 | 1.6E-05 |
| MOA | Differentiation | Activation of *HOX* genes during differentiation | R-HSA-5619507 | Reactome | - | 14.8 | 9.6 | 1.5 | 1.4E-06 |
| MOA | Differentiation | RUNX1 regulates transcription of genes involved in differentiation of HSCs | R-HSA-8939236 | Reactome | - | 26.8 | 20.2 | 1.3 | 7.7E-07 |
| MOA | Development | Meiotic recombination | R-HSA-912446 | Reactome | - | 4.4 | 0.9 | 4.7 | 1.0E-04 |
| MOA | Development | Reproduction | R-HSA-1474165 | Reactome | - | 1.5 | 0.4 | 4.1 | 3.8E-05 |
| MOA | Development | Meiosis | R-HSA-1500620 | Reactome | - | 4.6 | 1.6 | 2.9 | 1.8E-05 |
| MOA | Development | Activation of anterior *HOX* genes in hindbrain development during early embryogenesis | R-HSA-5617472 | Reactome | - | 14.8 | 9.6 | 1.5 | 1.4E-06 |
| MOA | Development | GO male germ cell nucleus | GO:0001673 | Gene Ontology | Cellular component | 4.9 | 3.1 | 1.6 | 2.6E-05 |
| MOA | Development | GO germ cell nucleus | GO:0043073 | Gene Ontology | Cellular component | 5.4 | 3.1 | 1.8 | 3.5E-06 |
| MOA | DNA repair | HDR through single strand annealing (SSA) | R-HSA-5685938 | Reactome | - | 8 | 2.9 | 2.7 | 1.5E-05 |
| MOA | DNA repair | GO mismatch repair complex | GO:0032300 | Gene Ontology | Cellular component | 25.2 | 18.1 | 1.4 | 5.7E-05 |
| MOA | DNA repair | Hallmark DNA repair | M5898 | MsigDB | Hallmark | 26.7 | 22.1 | 1.2 | 1.1E-05 |
| MOA | DNA repair | DNA repair | R-HSA-73894 | Reactome | - | 17.8 | 11.5 | 1.5 | 1.1E-06 |
| MOA | DNA repair | Recruitment and ATM-mediated phosphorylation of repair and signalling proteins at DNA double strand breaks | R-HSA-5693565 | Reactome | - | 16.7 | 10.5 | 1.6 | 3.7E-06 |
| MOA | DNA repair | HDR through MMEJ (alt-NHEJ) | R-HSA-5685939 | Reactome | - | 27.4 | 17.2 | 1.6 | 9.5E-06 |
| MOA | DNA repair | DNA double strand break response | R-HSA-5693606 | Reactome | - | 17.3 | 10.7 | 1.6 | 2.0E-06 |
| MOA | Hormones | Hallmark estrogen response late | M5907 | MsigDB | Hallmark | 17.6 | 19.3 | 0.9 | 5.6E-06 |
| MOA | Hormones | Hallmark estrogen response early | M5906 | MsigDB | Hallmark | 18.8 | 21.7 | 0.9 | 6.5E-06 |
| MOA | Hormones | Amine-derived hormones | R-HSA-209776 | Reactome | - | 0.9 | 1.8 | 0.5 | 7.9E-05 |
| MOA | Hormones | Activated PKN1 stimulates transcription of AR (androgen receptor) regulated genes *KLK2* and *KLK3* | R-HSA-5625886 | Reactome | - | 11.6 | 4.4 | 2.7 | 9.2E-06 |
| MOA | Hormones | KEGG steroid hormone biosynthesis | HSA00140 | KEGG | - | 20.9 | 33.2 | 0.6 | 2.2E-04 |
| MOA | Hormones | Growth hormone receptor signalling | R-HSA-982772 | Reactome | - | 20.2 | 24.5 | 0.8 | 1.1E-06 |
| MOA | Loss of hepatocytes feat. | Branched-chain amino acid catabolism | R-HSA-70895 | Reactome | - | 34.2 | 46 | 0.7 | 1.9E-05 |
| MOA | Loss of hepatocytes feat. | KEGG metabolism of xenobiotics by cytochrome P450 | HSA00980 | KEGG | - | 28.4 | 45.1 | 0.6 | 8.6E-05 |
| MOA | Loss of hepatocytes feat. | Hallmark xenobiotic metabolism | M5934 | MsigDB | Hallmark | 37.9 | 51.3 | 0.7 | 1.9E-05 |
| MOA | Loss of hepatocytes feat. | Fatty acids | R-HSA-211935 | Reactome | - | 30.7 | 50.8 | 0.6 | 5.2E-05 |
| MOA | Loss of hepatocytes feat. | Hallmark fatty acid metabolism | M5935 | MsigDB | Hallmark | 35 | 43.5 | 0.8 | 3.9E-05 |
| MOA | Loss of hepatocytes feat. | HAllmark bile acid metabolism | M5948 | MsigDB | Hallmark | 25.5 | 37.5 | 0.7 | 1.4E-05 |
| MOA | Loss of hepatocytes feat. | KEGG drug metabolism cytochrome P450 | HSA00982 | KEGG | - | 28.7 | 48.9 | 0.6 | 1.9E-05 |
| MOA | Loss of hepatocytes feat. | KEGG drug metabolism other enzymes | HSA00983 | KEGG | - | 32.2 | 52.6 | 0.6 | 3.0E-05 |
| MOA | Loss of hepatocytes feat. | Synthesis of (16-20)-hydroxyeicosatetraenoic acids (HETE) | R-HSA-2142816 | Reactome | - | 35.6 | 61.4 | 0.6 | 3.0E-05 |
| MOA | Loss of hepatocytes feat. | KEGG primary bile acid biosynthesis | HSA00120 | KEGG | - | 28.5 | 51.2 | 0.6 | 2.3E-06 |
| MOA | Methylation | Methylation | R-HSA-156581 | Reactome | - | 41.2 | 51.8 | 0.8 | 2.2E-04 |
| MOA | Methylation | GO methylosome | GO:0034709 | Gene Ontology | Cellular component | 31.4 | 21.1 | 1.5 | 9.4E-06 |
| MOA | Methylation | GO ESC E Z complex | GO:0035098 | Gene Ontology | Cellular component | 30.3 | 20.5 | 1.5 | 2.5E-06 |
| MOA | Methylation | DNA methylation | R-HSA-5334118 | Reactome | - | 10.9 | 3.5 | 3.1 | 1.0E-05 |
| MOA | Methylation | PRC2 methylates histones and DNA | R-HSA-212300 | Reactome | - | 13.7 | 5.2 | 2.6 | 3.7E-06 |
| MOA | Methylation | RMTs methylate histone arginines | R-HSA-3214858 | Reactome | - | 30.1 | 21 | 1.4 | 1.5E-07 |
| MOA | Metabolism | KEGG butanoate metabolism | HSA00650 | KEGG | - | 27.8 | 37.2 | 0.7 | 9.4E-05 |
| MOA | Metabolism | Signalling by retinoic acid | R-HSA-5362517 | Reactome | - | 20.3 | 25 | 0.8 | 2.2E-04 |
| MOA | Metabolism | KEGG arachidonic acid metabolism | HSA00590 | KEGG | - | 12.8 | 19.8 | 0.6 | 5.9E-06 |
| MOA | Metabolism | KEGG starch and sucrose metabolism | HSA00500 | KEGG | - | 19.4 | 29 | 0.7 | 3.8E-04 |
| MOA | Metabolism | KEGG glycine serine and threonine metabolism | HSA00260 | KEGG | - | 39.5 | 61.1 | 0.6 | 6.2E-06 |
| MOA | Metabolism | KEGG folate biosynthesis | HSA00790 | KEGG | - | 31.1 | 43.7 | 0.7 | 2.2E-06 |
| MOA | Metabolism | KEGG linoleic acid metabolism | HSA00591 | KEGG | - | 14.2 | 25.9 | 0.5 | 3.3E-06 |
| MOA | Metabolism | KEGG β alanine metabolism | HSA00410 | KEGG | - | 31.8 | 45.8 | 0.7 | 5.2E-06 |
| MOA | Metabolism | KEGG retinol metabolism | HSA00830 | KEGG | - | 23.9 | 42.5 | 0.6 | 1.2E-05 |
| MOA | Metabolism | KEGG tryptophan metabolism | HSA00380 | KEGG | - | 22.7 | 37.6 | 0.6 | 3.2E-07 |
| MOA | Metabolism | KEGG histidine metabolism | HSA00340 | KEGG | - | 25.8 | 36.8 | 0.7 | 8.7E-07 |
| MOA | Metabolism | KEGG sulphur metabolism | HSA00920 | KEGG | - | 31.6 | 38.3 | 0.8 | 3.0E-04 |
| MOA | Transcription | KEGG basal transcription factors | HSA03022 | KEGG | - | 12 | 8.6 | 1.4 | 3.0E-04 |
| MOA | Transcription | GO saga complex | GO:0000124 | Gene Ontology | Cellular component | 27.3 | 19.7 | 1.4 | 2.9E-07 |
| MOA | Transcription | RNA polymerase I promoter opening | R-HSA-73728 | Reactome | - | 15.1 | 5.7 | 2.6 | 8.7E-06 |
| MOA | Transcription | RNA polymerase I chain elongation | R-HSA-73777 | Reactome | - | 15.1 | 7.1 | 2.1 | 1.5E-06 |
| MOA | Transcription | NoRC negatively regulates rRNA expression | R-HSA-427413 | Reactome | - | 18.1 | 9.9 | 1.8 | 7.7E-07 |
| MOA | Transcription | HDACs deacetylate histones | R-HSA-3214815 | Reactome | - | 20.3 | 11.3 | 1.8 | 6.5E-07 |
| MOA | Transcription | HATs acetylate histones | R-HSA-3214847 | Reactome | - | 23.7 | 15.8 | 1.5 | 4.2E-07 |
| MOA | Transcription | B-WICH complex positively regulates rRNA expression | R-HSA-5250924 | Reactome | - | 20.3 | 11.5 | 1.8 | 7.5E-07 |
| MOA | Transcription | RNA polymerase I promoter clearance | R-HSA-73854 | Reactome | - | 20.1 | 11.8 | 1.7 | 6.3E-07 |
| MOA | Transcription | RNA polymerase I transcription | R-HSA-73857 | Reactome | - | 20.6 | 12.2 | 1.7 | 4.8E-07 |
| MOA | Transcription | Positive epigenetic regulation of rRNA expression | R-HSA-5250913 | Reactome | - | 23.9 | 14.8 | 1.6 | 3.6E-07 |
| MOA | Pathways in cancer | PI3KAKT signalling in cancer | R-HSA-8853334 | Reactome | - | 8.3 | 10.3 | 0.8 | 1.4E-06 |
| MOA | Pathways in cancer | Signalling by Wnt in cancer | R-HSA-4791275 | Reactome | - | 29.3 | 26.1 | 1.1 | 1.2E-03 |
| MOA | Pathways in cancer | Hallmark Wnt β-catenin signalling | M5895 | MsigDB | Hallmark | 23.8 | 19.2 | 1.2 | 5.1E-05 |
| MOA | Pathways in cancer | Down-regulation of ERBB2:ERBB3 signalling | R-HSA-1358803 | Reactome | - | 48.1 | 52.6 | 0.9 | 1.2E-02 |
| MOA | Pathways in cancer | Hallmark myc targets v1 | M5926 | MsigDB | Hallmark | 51.4 | 41.8 | 1.2 | 1.7E-06 |
| MOA | Pathways in cancer | GRB7 events in ERBB2 signalling | R-HSA-1306955 | Reactome | - | 39.1 | 47.4 | 0.8 | 2.7E-03 |
| MOA | Pathways in cancer | Deactivation of the β-catenin transactivating complex | R-HSA-3769402 | Reactome | - | 24.8 | 20.6 | 1.2 | 4.2E-05 |
| MOA | Pathways in cancer | Regulation of PTEN gene transcription | R-HSA-8943724 | Reactome | - | 29.8 | 25 | 1.2 | 5.8E-07 |
| MOA | Pathways in cancer | mTOR signalling | R-HSA-165159 | Reactome | - | 30.7 | 26.2 | 1.2 | 6.1E-06 |
| MOA | Pathways in cancer | Formation of the β-catenin:TCF transactivating complex | R-HSA-201722 | Reactome | - | 18.7 | 10.8 | 1.7 | 7.4E-07 |
| MOA | Pathways in cancer | SLIT2:ROBO1 increases RHOA activity | R-HSA-8985586 | Reactome | - | 44 | 28.5 | 1.5 | 1.1E-06 |
| MOA | Misc. - Up | KEGG systemic lupus erythematosus | HSA05322 | KEGG | - | 15.5 | 10.5 | 1.5 | 1.3E-04 |
| MOA | Misc. - Up | Hallmark protein secretion | M5910 | MsigDB | Hallmark | 40.4 | 35.4 | 1.1 | 1.0E-04 |
| MOA | Misc. - Up | Resolution of sister chromatid cohesion | R-HSA-2500257 | Reactome | - | 13 | 5.2 | 2.5 | 1.6E-06 |
| MOA | Misc. - Up | Export of viral ribonucleoproteins from nucleus | R-HSA-168274 | Reactome | - | 39.3 | 26 | 1.5 | 8.6E-07 |
| MOA | Misc. - Up | SIRT1 negatively regulates rRNA expression | R-HSA-427359 | Reactome | - | 11.9 | 4.1 | 2.9 | 4.4E-06 |
| MOA | Misc. - Up | AURKA activation by TPX2 | R-HSA-8854518 | Reactome | - | 17.4 | 10.7 | 1.6 | 5.7E-07 |
| MOA | Misc. - Up | SUMOylation | R-HSA-2990846 | Reactome | - | 30.5 | 21.3 | 1.4 | 1.9E-07 |
| MOA | Misc. - Up | Senescence-associated secretory phenotype (SASP) | R-HSA-2559582 | Reactome | - | 17.1 | 11 | 1.6 | 5.2E-06 |
| MOA | Misc. - Up | ERCC6 (CSB) and EHMT2 (G9a) positively regulate rRNA expression | R-HSA-427389 | Reactome | - | 17.3 | 8.1 | 2.1 | 1.7E-06 |
| MOA | Misc. - Up | KEGG small cell lung cancer | HSA05222 | KEGG | - | 20.3 | 15.9 | 1.3 | 1.8E-04 |
| MOA | Misc. - Up | Transferrin endocytosis and recycling | R-HSA-917977 | Reactome | - | 22.5 | 19.3 | 1.2 | 5.9E-04 |
| MOA | Misc. - Up | Formation of senescence-associated heterochromatin foci (SAHF) | R-HSA-2559584 | Reactome | - | 18.3 | 12.9 | 1.4 | 1.3E-06 |
| MOA | Misc. - Up | Activation of RAC1 | R-HSA-418856 | Reactome | - | 29.9 | 20.1 | 1.5 | 6.8E-08 |
| MOA | Misc. - Up | Iron uptake and transport | R-HSA-917937 | Reactome | - | 33 | 29.7 | 1.1 | 1.4E-04 |
| MOA | Misc. - Down | Interleukin-20 family signalling | R-HSA-8854691 | Reactome | - | 6.2 | 7.2 | 0.9 | 1.4E-03 |
| MOA | Misc. - Down | ZBP1(DAI) mediated induction of type I IFNs | R-HSA-1606322 | Reactome | - | 24.4 | 28.2 | 0.9 | 6.6E-04 |
| MOA | Misc. - Down | DExH-box helicases activate type I IFN and inflammatory cytokines production | R-HSA-3134963 | Reactome | - | 37.6 | 42.4 | 0.9 | 2.6E-04 |
| MOA | Misc. - Down | GO sodium channel complex | GO:0034706 | Gene Ontology | Cellular component | 0.1 | 0.1 | 0.5 | 2.8E-05 |
| MOA | Misc. - Down | KEGG ABC transporters | HSA02010 | KEGG | - | 12.7 | 18.3 | 0.7 | 8.7E-04 |
| MOA | Misc. - Down | Aflatoxin activation and detoxification | R-HSA-5423646 | Reactome | - | 33.2 | 49.5 | 0.7 | 2.3E-07 |

**Supplementary Table 2: Baseline clinicodemographic features of the Peruvian and non-Amerind HCC patients scrutinized using Gene Expression Omnibus (GEO) HCC/NTL datasets from Affymetrix Human Genome U133 Plus 2.0 array.**

|  | **Peruvian patients** | | | **non-Amerind patients** | | | | **Statistical significance (Peruvian vs. elsewhere)** | |
| --- | --- | --- | --- | --- | --- | --- | --- | --- | --- |
|  | **Overall**  **(*n* = 39)** | **AYA**  **(*n* = 19)** | **MOA**  **(*n* = 20)** | **Overall**  **(*n* = 170)** | **France**  **(*n* = 81)** | **Taiwan**  **(*n* = 72)** | **Turkey**  **(*n* = 17)** |  |  |
| **GEO series** | GSE111580, GSE136247 | | |  | GSE45436 | GSE62232 | GSE17548 |  |  |
| **Gender** |  |  |  |  |  |  |  | *p* < 0.05 (χ^2^ test) |  |
| Male | 27 (69.2%) | 13 (68.4%) | 14 (70%) | 128 (75.3%) | 67 (82.7%) | 46 (63.9%) | 15 (88.2%) |  |  |
| Female | 12 (30.8%) | 6 (31.6%) | 6 (30%) | 42 (24.7%) | 14 (17.3%) | 26 (36.1%) | 2 (11.8%) |  |  |
| **Age (years)** |  |  |  |  |  |  |  | *p* < 0.0001 (*t*-test) |  |
| Mean ± SD | 50.3 ± 22.1 | 30.4 ± 9.2 | 69.1 ± 11.5 | 56.4 ± 23.8 | 60.6 ± 13.6 | 49.6 ± 25.5 | 56.4 ± 23.3 |  |  |
| Median | 46 | 32 | 71 | 59 | 63 | 51 | 57.5 |  |  |
| Range | [13–94] | [13–94] | [13–94] | [13–94] | [13–94] | [13–94] | [13–94] |  |  |
| **HBV infection** |  |  |  |  |  |  |  | *p* < 0.0001 (χ^2^ test) | |
| Positive | 26 (66.7%) | 15 (78.9%) | 11 (55%) | 26 (15.3%) | 16 (80.3%) | - | 10 (58.8%) |  |  |
| Negative | 12 (33.3%) | 3 (21.1%) | 9 (45%) | 68 (40%) | 65 (19.7%) | - | 3 (17.7%) |  |  |
| ND | - | - | - | 76 (44.7%) | - | 72 (100) | 4 (23.5%) |  |  |

**Supplementary Table 3: Results of HCC/NTL SES analysis in non-Amerind patients.**

| **Gene set class** | **Gene set** | **Database ID** | **Database** | **Collection** | **HCC SES mean** | **NTL SES mean** | **Fold change** | ***p*-value** |
| --- | --- | --- | --- | --- | --- | --- | --- | --- |
| Cell cycle | Activation of the pre-replicative complex | R-HSA-68962 | Reactome | - | 11.37 | 2.36 | 4.81 | 5.10E-14 |
| Cell cycle | Activation of ATR in response to replication stress | R-HSA-176187 | Reactome | - | 8.13 | 1.44 | 5.65 | 3.17E-13 |
| Cell cycle | Activation of E2F1 target genes at G1S | R-HSA-539107 | Reactome | - | 9.91 | 3.28 | 3.02 | 2.06E-14 |
| Cell cycle | DNA strand elongation | R-HSA-69190 | Reactome | - | 16.58 | 4.12 | 4.02 | 1.13E-18 |
| Cell cycle | KEGG DNA replication | HSA03030 | KEGG | - | 19.41 | 7.57 | 2.57 | 3.32E-20 |
| Cell cycle | TP53 regulates transcription of genes involved in G1 cell cycle arrest | R-HSA-6804116 | Reactome | - | 5.27 | 1.59 | 3.31 | 3.56E-12 |
| Cell cycle | Unwinding of DNA | R-HSA-176974 | Reactome | - | 16.17 | 3.16 | 5.11 | 2.55E-15 |
| Cell cycle | MET activates PTK2 signalling | R-HSA-8874081 | Reactome | - | 7.34 | 6.23 | 1.18 | 7.73E-03 |
| Cell cycle | KEGG cell cycle | HSA04110 | KEGG | - | 12.39 | 5.85 | 2.12 | 2.04E-23 |
| Cell cycle | DNA replication | R-HSA-69306 | Reactome | - | 27.26 | 16.34 | 1.67 | 2.08E-23 |
| Cell cycle | TP53 regulates transcription of cell cycle genes | R-HSA-6791312 | Reactome | - | 9.25 | 4.46 | 2.07 | 1.04E-21 |
| Cell cycle | GO MCM complex | GO:0042555 | Gene Ontology | Cellular component | 30.91 | 9.25 | 3.34 | 1.12E-03 |
| Chromosome | GO centriole | GO:0005814 | Gene Ontology | Cellular component | 2.35 | 0.82 | 2.86 | 7.12E-05 |
| Chromosome | GO microtubule organizing centre part | GO:0005815 | Gene Ontology | Cellular component | 2.84 | 1.27 | 2.23 | 5.07E-06 |
| Chromosome | GO condensed chromosome centromeric region | GO:0000780 | Gene Ontology | Cellular component | 10.92 | 3.15 | 3.47 | 1.24E-05 |
| Chromosome | Chromosome maintenance | GO:0000780 | Gene Ontology | Cellular component | 4.36 | 1.09 | 4 | 1.21E-21 |
| Chromosome | Condensation of prophase chromosomes | R-HSA-73886 | Reactome | - | 1.69 | 0.61 | 2.77 | 2.73E-15 |
| Chromosome | GO condensed chromosome | GO:0000793 | Gene Ontology | Cellular component | 5.18 | 1.23 | 4.22 | 3.66E-05 |
| Chromosome | GO chromosome centromeric region | GO:0000775 | Gene Ontology | Cellular component | 10.31 | 3.79 | 2.72 | 9.47E-06 |
| Chromosome | Packaging of telomere ends | R-HSA-171306 | Reactome | - | 1.71 | 0.72 | 2.38 | 1.17E-10 |
| Chromosome | DNA damage/telomere stress induced senescence | R-HSA-2559586 | Reactome | - | 1.94 | 0.66 | 2.92 | 4.82E-19 |
| Chromosome | GO chromosome | GO:0005694 | Gene Ontology | Cellular component | 9.02 | 4.35 | 2.07 | 1.14E-05 |
| Chromosome | GO chromosome telomeric region | GO:0098687 | Gene Ontology | Cellular component | 4.42 | 1.96 | 2.25 | 6.23E-06 |
| Differentiation | GO main axon | GO:0030426 | Gene Ontology | Cellular component | 0.27 | 0.63 | 0.43 | 8.44E-07 |
| Differentiation | GO synaptonemal complex | GO:0007130 | Gene Ontology | Cellular component | 0.15 | 0.07 | 2.06 | 2.67E-06 |
| Differentiation | RUNX3 regulates YAP1-mediated transcription | R-HSA-8951671 | Reactome | - | 27.45 | 26.71 | 1.03 | 2.29E-01 |
| Differentiation | RUNX1 regulates genes involved in megakaryocyte differentiation and platelet function | R-HSA-8936459 | Reactome | - | 1.79 | 1.3 | 1.38 | 4.49E-06 |
| Differentiation | Activation of *HOX* genes during differentiation | R-HSA-5619507 | Reactome | - | 4.21 | 2.9 | 1.45 | 4.15E-15 |
| Differentiation | RUNX1 regulates transcription of genes involved in differentiation of HSCs | R-HSA-8939236 | Reactome | - | 16.47 | 13.96 | 1.18 | 1.94E-12 |
| Development | Meiotic recombination | R-HSA-912446 | Reactome | - | 0.5 | 0.08 | 6.56 | 1.52E-14 |
| Development | Reproduction | R-HSA-1474165 | Reactome | - | 0.13 | 0.02 | 6.27 | 1.39E-11 |
| Development | Meiosis | R-HSA-1500620 | Reactome | - | 0.73 | 0.21 | 3.42 | 3.48E-15 |
| Development | Activation of anterior *HOX* genes in hindbrain development during early embryogenesis | R-HSA-5617472 | Reactome | - | 4.21 | 2.9 | 1.45 | 4.15E-15 |
| Development | GO male germ cell nucleus | GO:0001673 | Gene Ontology | Cellular component | 2.06 | 0.93 | 2.21 | 1.93E-03 |
| Development | GO germ cell nucleus | GO:0043073 | Gene Ontology | Cellular component | 1.55 | 0.49 | 3.19 | 1.12E-03 |
| DNA repair | HDR through Single Strand Annealing (SSA) | R-HSA-5685938 | Reactome | - | 7.89 | 3.2 | 2.46 | 3.83E-19 |
| DNA repair | GO mismatch repair complex | GO:0032300 | Gene Ontology | Cellular component | 8.98 | 4.95 | 1.81 | 1.03E-06 |
| DNA repair | Hallmark DNA repair | M5898 | MsigDB | Hallmark | 18.97 | 16.6 | 1.14 | 3.05E-02 |
| DNA repair | DNA repair | R-HSA-73894 | Reactome | - | 9.25 | 5.37 | 1.72 | 3.95E-28 |
| DNA repair | Recruitment and ATM-mediated phosphorylation of repair and signalling proteins at DNA double strand breaks | R-HSA-5693565 | Reactome | - | 4.31 | 2.67 | 1.61 | 2.31E-19 |
| DNA repair | HDR through MMEJ (alt-NHEJ) | R-HSA-5685939 | Reactome | - | 20.93 | 13.55 | 1.54 | 1.89E-23 |
| DNA repair | DNA double strand break response | R-HSA-5693606 | Reactome | - | 4.76 | 2.93 | 1.63 | 5.94E-21 |
| Hormones | Hallmark oestrogen response late | M5907 | MsigDB | Hallmark | 5.88 | 6.93 | 0.85 | 3.98E-02 |
| Hormones | Hallmark oestrogen response early | M5906 | MsigDB | Hallmark | 6.42 | 8.22 | 0.78 | 3.40E-03 |
| Hormones | Amine-derived hormones | R-HSA-209776 | Reactome | - | 0.04 | 0.08 | 0.54 | 7.38E-09 |
| Hormones | Activated PKN1 stimulates transcription of AR (androgen receptor) regulated genes *KLK2* and *KLK3* | R-HSA-5625886 | Reactome | - | 1.25 | 0.44 | 2.83 | 1.03E-11 |
| Hormones | KEGG steroid hormone biosynthesis | HSA00140 | KEGG | - | 17.83 | 25.12 | 0.71 | 1.52E-10 |
| Hormones | Growth hormone receptor signalling | R-HSA-982772 | Reactome | - | 11.88 | 16.23 | 0.73 | 1.95E-20 |
| Loss of hepatocytes feat. | Branched-chain amino acid catabolism | R-HSA-70895 | Reactome | - | 35.24 | 44.4 | 0.79 | 1.22E-12 |
| Loss of hepatocytes feat. | KEGG metabolism of xenobiotics by cytochrome P450 | HSA00980 | KEGG | - | 24.23 | 34.15 | 0.71 | 1.60E-12 |
| Loss of hepatocytes feat. | Hallmark xenobiotic metabolism | M5934 | MsigDB | Hallmark | 31.34 | 37.68 | 0.83 | 2.88E-02 |
| Loss of hepatocytes feat. | Fatty acids | R-HSA-211935 | Reactome | - | 16.17 | 30.05 | 0.54 | 1.05E-19 |
| Loss of hepatocytes feat. | Hallmark fatty acid metabolism | M5935 | MsigDB | Hallmark | 28.07 | 33.04 | 0.85 | 2.47E-02 |
| Loss of hepatocytes feat. | Hallmark bile acid metabolism | M5948 | MsigDB | Hallmark | 18.67 | 22.48 | 0.83 | 9.79E-02 |
| Loss of hepatocytes feat. | KEGG drug metabolism cytochrome P450 | HSA00982 | KEGG | - | 27.94 | 40.81 | 0.68 | 9.69E-15 |
| Loss of hepatocytes feat. | KEGG drug metabolism other enzymes | HSA00983 | KEGG | - | 34.02 | 45.69 | 0.74 | 2.48E-12 |
| Loss of hepatocytes feat. | Synthesis of (16-20)-hydroxyeicosatetraenoic acids (HETE) | R-HSA-2142816 | Reactome | - | 39.77 | 62.43 | 0.64 | 8.18E-17 |
| Loss of hepatocytes feat. | KEGG primary bile acid biosynthesis | HSA00120 | KEGG | - | 31.01 | 46.55 | 0.67 | 3.72E-12 |
| Methylation | Methylation | R-HSA-156581 | Reactome | - | 53.96 | 63.59 | 0.85 | 1.25E-12 |
| Methylation | GO methylosome | GO:0034709 | Gene Ontology | Cellular component | 60.66 | 51.77 | 1.17 | 3.81E-01 |
| Methylation | GO ESC E Z complex | GO:0035098 | Gene Ontology | Cellular component | 30 | 22.13 | 1.36 | 9.03E-06 |
| Methylation | DNA methylation | R-HSA-5334118 | Reactome | - | 0.95 | 0.2 | 4.73 | 7.93E-14 |
| Methylation | PRC2 methylates histones and DNA | R-HSA-212300 | Reactome | - | 3.21 | 1.09 | 2.93 | 1.17E-20 |
| Methylation | RMTs methylate histone arginines | R-HSA-3214858 | Reactome | - | 8.55 | 5.15 | 1.66 | 4.41E-24 |
| Metabolism | KEGG butanoate metabolism | HSA00650 | KEGG | - | 24.74 | 29.84 | 0.83 | 3.35E-09 |
| Metabolism | Signalling by retinoic acid | R-HSA-5362517 | Reactome | - | 15.31 | 20.01 | 0.76 | 3.20E-17 |
| Metabolism | KEGG arachidonic acid metabolism | HSA00590 | KEGG | - | 3.77 | 7.98 | 0.47 | 4.79E-30 |
| Metabolism | KEGG starch and sucrose metabolism | HSA00500 | KEGG | - | 16.43 | 20.3 | 0.81 | 3.16E-06 |
| Metabolism | KEGG glycine serine and threonine metabolism | HSA00260 | KEGG | - | 33.88 | 48.87 | 0.69 | 9.44E-20 |
| Metabolism | KEGG folate biosynthesis | HSA00790 | KEGG | - | 18.38 | 24.13 | 0.76 | 3.60E-16 |
| Metabolism | KEGG linoleic acid metabolism | HSA00591 | KEGG | - | 6.18 | 11 | 0.56 | 6.98E-20 |
| Metabolism | KEGG β alanine metabolism | HSA00410 | KEGG | - | 27.48 | 38.35 | 0.72 | 3.19E-23 |
| Metabolism | KEGG retinol metabolism | HSA00830 | KEGG | - | 22.39 | 37.36 | 0.6 | 4.71E-20 |
| Metabolism | KEGG tryptophan metabolism | HSA00380 | KEGG | - | 18.31 | 30.65 | 0.6 | 1.49E-30 |
| Metabolism | KEGG histidine metabolism | HSA00340 | KEGG | - | 13.42 | 20.49 | 0.65 | 8.58E-29 |
| Metabolism | KEGG sulphur metabolism | HSA00920 | KEGG | - | 24.8 | 30.72 | 0.81 | 4.19E-14 |
| Transcription | KEGG basal transcription factors | HSA03022 | KEGG | - | 9.27 | 6.21 | 1.49 | 5.11E-23 |
| Transcription | GO SAGA complex | GO:0000124 | Gene Ontology | Cellular component | 14.99 | 13.03 | 1.15 | 3.15E-03 |
| Transcription | RNA polymerase I promoter opening | R-HSA-73728 | Reactome | - | 1.81 | 0.77 | 2.35 | 8.19E-11 |
| Transcription | RNA polymerase I chain elongation | R-HSA-73777 | Reactome | - | 1.81 | 0.77 | 2.35 | 8.19E-11 |
| Transcription | NoRC negatively regulates rRNA expression | R-HSA-427413 | Reactome | - | 6.55 | 3.82 | 1.72 | 3.43E-23 |
| Transcription | HDACs deacetylate histones | R-HSA-3214815 | Reactome | - | 3.4 | 1.91 | 1.78 | 1.32E-16 |
| Transcription | HATs acetylate histones | R-HSA-3214847 | Reactome | - | 5.56 | 2.88 | 1.93 | 3.18E-22 |
| Transcription | B-WICH complex positively regulates rRNA expression | R-HSA-5250924 | Reactome | - | 5.58 | 3.5 | 1.59 | 3.34E-16 |
| Transcription | RNA polymerase I promoter clearance | R-HSA-73854 | Reactome | - | 6.7 | 4.21 | 1.59 | 3.13E-21 |
| Transcription | RNA polymerase I transcription | R-HSA-73857 | Reactome | - | 7.26 | 4.71 | 1.54 | 1.48E-21 |
| Transcription | Positive epigenetic regulation of rRNA expression | R-HSA-5250913 | Reactome | - | 8.33 | 5.55 | 1.5 | 2.08E-21 |
| Pathways in cancer | PI3KAKT Signalling in Cancer | R-HSA-8853334 | Reactome | - | 0.75 | 1.14 | 0.66 | 7.43E-12 |
| Pathways in cancer | Signalling by Wnt in cancer | R-HSA-4791275 | Reactome | - | 15.35 | 13.28 | 1.16 | 4.51E-07 |
| Pathways in cancer | Hallmark Wnt β-catenin signalling | M5895 | MsigDB | Hallmark | 6.81 | 6.26 | 1.09 | 3.89E-01 |
| Pathways in cancer | Downregulation of ERBB2:ERBB3 signalling | R-HSA-1358803 | Reactome | - | 28.95 | 29.1 | 0.99 | 7.37E-01 |
| Pathways in cancer | Hallmark myc targets v1 | M5926 | MsigDB | Hallmark | 53.35 | 48.27 | 1.11 | 6.50E-02 |
| Pathways in cancer | GRB7 events in ERBB2 signalling | R-HSA-1306955 | Reactome | - | 12.77 | 12.67 | 1.01 | 8.29E-01 |
| Pathways in cancer | Deactivation of the β-catenin transactivating complex | R-HSA-3769402 | Reactome | - | 14.4 | 11.95 | 1.2 | 4.57E-09 |
| Pathways in cancer | Regulation of PTEN gene transcription | R-HSA-8943724 | Reactome | - | 16.03 | 13.02 | 1.23 | 4.43E-14 |
| Pathways in cancer | mTOR signalling | R-HSA-165159 | Reactome | - | 23.17 | 20.98 | 1.1 | 6.84E-07 |
| Pathways in cancer | Formation of the β-catenin:TCF transactivating complex | R-HSA-201722 | Reactome | - | 3.08 | 1.57 | 1.96 | 2.05E-16 |
| Pathways in cancer | SLIT2:ROBO1 increases RHOA activity | R-HSA-8985586 | Reactome | - | 40.93 | 34.09 | 1.2 | 8.08E-04 |
| Misc. - Up | KEGG systemic lupus erythematosus | HSA05322 | KEGG | - | 0.41 | 0.29 | 1.42 | 1.35E-02 |
| Misc. - Up | Hallmark protein secretion | M5910 | MsigDB | Hallmark | 36.06 | 34.2 | 1.05 | 2.47E-01 |
| Misc. - Up | Resolution of sister chromatid cohesion | R-HSA-2500257 | Reactome | - | 13.05 | 4.49 | 2.91 | 2.40E-22 |
| Misc. - Up | Export of viral ribonucleoproteins from nucleus | R-HSA-168274 | Reactome | - | 32.26 | 24.94 | 1.29 | 5.50E-16 |
| Misc. - Up | SIRT1 negatively regulates rRNA expression | R-HSA-427359 | Reactome | - | 1.25 | 0.4 | 3.09 | 1.36E-12 |
| Misc. - Up | AURKA activation by TPX2 | R-HSA-8854518 | Reactome | - | 17.57 | 11.18 | 1.57 | 5.03E-22 |
| Misc. - Up | SUMOylation | R-HSA-2990846 | Reactome | - | 12.98 | 7.87 | 1.65 | 1.65E-25 |
| Misc. - Up | Senescence-associated secretory phenotype (SASP) | R-HSA-2559582 | Reactome | - | 7.59 | 5.39 | 1.41 | 9.20E-16 |
| Misc. - Up | ERCC6 (CSB) and EHMT2 (G9a) positively regulate rRNA expression | R-HSA-427389 | Reactome | - | 3.63 | 1.78 | 2.05 | 1.18E-18 |
| Misc. - Up | KEGG small cell lung cancer | HSA05222 | KEGG | - | 7.17 | 5.7 | 1.26 | 4.40E-08 |
| Misc. - Up | Transferrin endocytosis and recycling | R-HSA-917977 | Reactome | - | 14.55 | 12.49 | 1.16 | 1.07E-08 |
| Misc. - Up | Formation of senescence-associated heterochromatin foci (SAHF) | R-HSA-2559584 | Reactome | - | 5.13 | 3.52 | 1.46 | 3.47E-11 |
| Misc. - Up | Activation of RAC1 | R-HSA-418856 | Reactome | - | 16.22 | 14.33 | 1.13 | 8.90E-03 |
| Misc. - Up | Iron uptake and transport | R-HSA-917937 | Reactome | - | 23.87 | 23.44 | 1.02 | 3.91E-01 |
| Misc. - Down | Interleukin-20 family signalling | R-HSA-8854691 | Reactome | - | 2.79 | 3.79 | 0.74 | 5.02E-11 |
| Misc. - Down | ZBP1(DAI) mediated induction of type I IFNs | R-HSA-1606322 | Reactome | - | 12.8 | 15.52 | 0.82 | 4.48E-11 |
| Misc. - Down | DExH-box helicases activate type I IFN and inflammatory cytokines production | R-HSA-3134963 | Reactome | - | 19.63 | 25.17 | 0.78 | 5.57E-15 |
| Misc. - Down | GO sodium channel complex | GO:0034706 | Gene Ontology | Cellular component | 0 | 0 | 0.94 | 4.87E-01 |
| Misc. - Down | KEGG ABC transporters | HSA02010 | KEGG | - | 3.02 | 4.09 | 0.74 | 1.14E-06 |
| Misc.- Down | Aflatoxin activation and detoxification | R-HSA-5423646 | Reactome | - | 21.17 | 32.11 | 0.66 | 7.88E-28 |

**Supplementary Table 4: Data on the 961 HCC/NTL-differentially expressed genes constituent of the Amerind signature**

| **Gene symbol (HGNC)** | **Expression** | **Fold change (log2)** | ***q*-value** |
| --- | --- | --- | --- |
| *AAAS* | Up | 1.13312998137014 | 3.20864031950604E-36 |
| *AACS* | Up | 1.26162609183678 | 8.63210038295593E-22 |
| *AADAT* | Up | 0.651133248255484 | 0.000387563713610574 |
| *ABAT* | Down | -0.808260547003901 | 0.00239329409354546 |
| *ABCA10* | Down | -0.813571154841584 | 8.21558504383644E-13 |
| *ABCA12* | Up | 0.455982315630519 | 3.33761498842088E-21 |
| *ABCA13* | Down | -0.299066906484071 | 1.98909245197821E-09 |
| *ABCA4* | Up | 0.438703935262795 | 0.0000825813089812728 |
| *ABCA6* | Down | -1.32617628244152 | 0.0000469665310720128 |
| *ABCA7* | Up | 0.646455955460821 | 2.71157172033738E-14 |
| *ABCB11* | Down | -1.59990823232873 | 3.87882857767315E-06 |
| *ABCB6* | Up | 1.06455577233757 | 3.05100925532392E-27 |
| *ABCC10* | Up | 0.926982017295361 | 7.31633717409462E-17 |
| *ABCC11* | Up | 0.431826474701581 | 0.0000537195851142406 |
| *ABCC12* | Up | 0.491998279138099 | 1.27206678377E-14 |
| *ABCC5* | Up | 0.943756468162693 | 1.53752297287926E-15 |
| *ABCC9* | Down | -0.575219662681528 | 0.00414348738076348 |
| *ABCD1* | Up | 0.611092673384451 | 2.60869401419222E-08 |
| *ABCD4* | Up | 0.293094315104472 | 0.00211189171921087 |
| *ABCG2* | Down | -0.948079812918321 | 0.000814203533931692 |
| *ABCG4* | Down | -0.480919683859677 | 0.0000486161140252671 |
| *ABLIM1* | Up | 0.607912101551756 | 0.000003253424415988 |
| *ACAA2* | Down | -0.584706251355314 | 0.00210245068584686 |
| *ACADM* | Down | -1.35893759216231 | 4.61767862853828E-11 |
| *ACADS* | Down | -0.283609730027281 | 0.0105552683226245 |
| *ACADSB* | Down | -0.745940268509116 | 0.000800909326915039 |
| *ACAT1* | Down | -0.756367077092662 | 0.0000293499947053219 |
| *ACO1* | Down | -0.485883814470045 | 0.00224258124702396 |
| *ACOX2* | Down | -0.481537322572454 | 0.0234203790874686 |
| *ACP1* | Up | 0.437355824818098 | 0.000069826699273462 |
| *ACSL4* | Up | 1.1724762901285 | 0.00149294225979708 |
| *ACSM1* | Up | 0.550801121539187 | 0.0263128623088611 |
| *ACTN1* | Up | 1.10438573308344 | 6.15440932066922E-18 |
| *ACTN4* | Up | 1.58987778271464 | 1.05938128629872E-36 |
| *ADAM10* | Up | 0.490383634513309 | 0.002214933356465 |
| *ADAM17* | Up | 1.62086369165347 | 4.31241130801208E-37 |
| *ADH1A* | Down | -1.33688827109654 | 0.000799809078857535 |
| *ADH1B* | Down | -1.41135575079409 | 0.00189676985072085 |
| *ADH1C* | Down | -1.32473664597313 | 0.00267059055223999 |
| *ADH4* | Down | -1.7898851180485 | 0.00268080781835768 |
| *ADRM1* | Up | 0.553228920966477 | 1.40693577576501E-09 |
| *AEBP2* | Up | 0.504880610831658 | 0.00016706771296351 |
| *AFF1* | Up | 0.441840511116707 | 0.000332559516766828 |
| *AGBL4* | Down | -0.940908056262895 | 3.70907927410716E-17 |
| *AHCTF1* | Up | 0.826671527453053 | 1.60855342863138E-09 |
| *AHI1* | Up | 1.86642356108274 | 2.90776184392123E-31 |
| *AK3* | Down | -0.405678297515584 | 0.0191042338170859 |
| *AKR1C4* | Down | -1.26227200623527 | 0.00073192883027028 |
| *AKT1* | Up | 0.935037465840995 | 7.58441644134333E-12 |
| *AKT2* | Down | -0.42953661752541 | 0.0000240920671504431 |
| *ALAD* | Down | -0.429954262009735 | 0.0232511885956801 |
| *ALDH1A2* | Up | 1.33642457635573 | 1.20440281789714E-20 |
| *ALDH1A3* | Up | 1.14501526036234 | 1.39931113480396E-08 |
| *ALDH6A1* | Down | -0.568976290045626 | 0.0155106321638903 |
| *ALDH9A1* | Down | -0.351074758761204 | 0.00879393881045404 |
| *ALDOA* | Up | 0.341250646492814 | 0.0349271499104916 |
| *ALMS1* | Up | 1.91380346626311 | 1.12445293731126E-41 |
| *ALPL* | Up | 0.56337575821421 | 0.00418774701477325 |
| *ALPP* | Down | -0.745081061560511 | 2.39660661125671E-09 |
| *AMY1A* | Up | 0.880684156064497 | 0.0000358868234056869 |
| *AMY1B* | Up | 0.880684156064497 | 0.0000358868234056869 |
| *AMY1C* | Up | 0.880684156064497 | 0.0000358868234056869 |
| *AMY2A* | Up | 0.763572848740153 | 0.000353278512780894 |
| *ANAPC4* | Up | 1.31121857252299 | 5.98433123346182E-26 |
| *ANAPC7* | Up | 1.01574319447864 | 9.23425704701938E-34 |
| *ANGPTL3* | Down | -1.35696083662467 | 0.0000509696219763966 |
| *ANP32E* | Up | 0.558105513801971 | 9.98995356925951E-07 |
| *ANXA9* | Up | 0.449193802072731 | 0.00773624485572305 |
| *AOC3* | Up | 0.801887458185178 | 4.91031276260392E-08 |
| *AP2B1* | Down | -0.900491473345275 | 2.00067328817032E-06 |
| *AP3B1* | Up | 0.592745000079888 | 3.5495521517508E-07 |
| *APAF1* | Up | 0.588902954881276 | 2.13531550065018E-08 |
| *AQP7* | Up | 0.58895647397479 | 1.39459803816554E-06 |
| *AQP9* | Down | -1.57223943268396 | 0.000221738044920015 |
| *AR* | Down | -1.33970482522128 | 4.04177899433911E-07 |
| *AREG* | Up | 1.31589264247976 | 5.87461725130933E-12 |
| *ARFGAP3* | Up | 0.679763515691183 | 6.51117711000143E-08 |
| *ARFGEF1* | Up | 0.427717470122862 | 0.0000878892443043904 |
| *ARFGEF2* | Down | -0.651890608361297 | 0.000557293390694829 |
| *ARL6IP1* | Down | -0.470737397759144 | 0.00312853090702025 |
| *ATN1* | Up | 1.05306367047105 | 6.54584911140543E-23 |
| *ATOH8* | Up | 0.652345581377625 | 1.74815634176907E-16 |
| *ATP1A1* | Up | 0.409208213577786 | 0.00814768679045245 |
| *ATP2B4* | Up | 0.882748801957555 | 1.31689125748847E-08 |
| *ATP6AP1* | Up | 0.275058812174422 | 0.0184930922492216 |
| *ATP6V0A1* | Up | 0.788962520114493 | 3.31262749687408E-13 |
| *ATP6V0A2* | Up | 0.456711325404876 | 0.00021066343154779 |
| *ATP6V0A4* | Up | 0.372513494483323 | 2.48589566182617E-10 |
| *ATP6V1E1* | Up | 0.907184823453115 | 4.40904525449558E-18 |
| *ATP6V1E2* | Down | -0.36686207963775 | 0.00238042611172446 |
| *ATP6V1F* | Up | 0.540636366248215 | 1.436226463683E-10 |
| *ATXN7L3* | Up | 1.14553533357083 | 9.90848528147175E-35 |
| *AURKA* | Up | 1.12572648234243 | 6.24298530074596E-08 |
| *AURKB* | Up | 0.748362969513146 | 9.69803877246486E-08 |
| *BANF1* | Up | 0.736007024674812 | 4.94203459212501E-14 |
| *BATF* | Up | 0.98421789392944 | 3.93198567241882E-15 |
| *BAZ1B* | Up | 0.435813115520244 | 3.15414504020233E-06 |
| *BBS4* | Up | 0.529512134577099 | 2.4326777128324E-07 |
| *BCAM* | Up | 1.12696476132797 | 2.64946538818175E-15 |
| *BCAP31* | Up | 0.680254071419951 | 2.11683119025139E-08 |
| *BCAR1* | Up | 0.593584922635317 | 3.41444614214933E-23 |
| *BCAR3* | Down | -0.969057941987805 | 2.15143085050624E-10 |
| *BCAT1* | Up | 1.09820620712364 | 8.51664990930249E-07 |
| *BCL2L1* | Up | 1.12829805457768 | 1.39495475695655E-21 |
| *BCL9* | Up | 0.796925348764508 | 1.09975782975891E-13 |
| *BCL9L* | Up | 1.40800923915055 | 2.55604617048524E-25 |
| *BDH1* | Down | -0.612905202538338 | 0.0139769557690435 |
| *BDH2* | Up | 0.70404893980347 | 0.0000255118646495543 |
| *BIRC3* | Up | 1.22843756927658 | 0.0000332209403735718 |
| *BLM* | Up | 1.4117306544527 | 2.07962215511607E-21 |
| *BLVRA* | Up | 1.26273084979697 | 9.01234637561356E-16 |
| *BMP6* | Up | 0.388800099852816 | 0.00283839722746906 |
| *BMPR1B* | Up | 1.56053286492366 | 5.04135554911361E-34 |
| *BRCA1* | Up | 0.898520434348337 | 4.02073892338094E-12 |
| *BRF2* | Up | 0.383967383425243 | 9.72089826627145E-07 |
| *BTG3* | Up | 0.656210577030395 | 2.53737878084523E-06 |
| *BUB1* | Up | 1.37244067971236 | 7.42247240900172E-12 |
| *BUB1B* | Up | 1.2355866162812 | 5.01206388280552E-09 |
| *BUB3* | Up | 0.523869665813725 | 2.28396114470732E-07 |
| *C1QA* | Up | 1.15773971933473 | 6.52291811177521E-08 |
| *C1QB* | Up | 1.06666454528236 | 4.99268898525701E-06 |
| *C1QC* | Up | 0.756740375162089 | 0.000455451124083185 |
| *C1S* | Down | -0.534419146425204 | 0.0272016513690282 |
| *C7* | Up | 2.15259674098766 | 4.24554916472296E-07 |
| *C8B* | Down | -0.744987761191167 | 0.00495895163055999 |
| *CA4* | Down | -0.496201389768062 | 2.07638647030391E-08 |
| *CABIN1* | Up | 1.52030173205625 | 3.69816523111155E-42 |
| *CAD* | Up | 1.1367872785584 | 3.41129429608469E-18 |
| *CALB2* | Down | -0.805923015802872 | 1.96363667643509E-12 |
| *CALCR* | Up | 0.463555264452382 | 0.0000101653254951458 |
| *CAND1* | Up | 0.638067869615356 | 3.19108479441495E-08 |
| *CANT1* | Up | 1.52395310700687 | 4.22164214591378E-28 |
| *CASP9* | Up | 0.359954356085159 | 0.000279469320135883 |
| *CBFA2T3* | Up | 0.999496363664782 | 3.80236001854148E-19 |
| *CBR3* | Up | 0.242154328021082 | 0.00348204503486515 |
| *CBX2* | Up | 0.380798276268066 | 0.00259731596599027 |
| *CBX3* | Up | 0.479767805413058 | 9.35785063146716E-07 |
| *CBX6* | Up | 1.09196026274382 | 8.52838944484345E-16 |
| *CBX8* | Up | 0.70680798701626 | 4.9230358976643E-18 |
| *CCDC78* | Up | 0.142650045432387 | 0.0193296803130054 |
| *CCDC92* | Up | 0.690615274508543 | 5.95966349616286E-09 |
| *CCDC99* | Up | 0.655636723733093 | 3.19667591489269E-07 |
| *CCNA1* | Up | 0.61616646102108 | 2.3361648748002E-16 |
| *CCND2* | Up | 0.849778792286914 | 0.0000212051923592458 |
| *CCNE1* | Up | 0.427927615217521 | 0.00458627375904426 |
| *CCNF* | Up | 1.05259770543824 | 2.1546978258877E-27 |
| *CCT3* | Up | 0.398882719469173 | 0.000581942902072219 |
| *CCT5* | Up | 0.800286582950264 | 4.07271749669096E-13 |
| *CCT7* | Up | 0.387080017238299 | 0.0000183964902201762 |
| *CD1D* | Up | 0.974353120088989 | 3.29435662150958E-06 |
| *CD80* | Up | 0.641115285705877 | 3.48381836221567E-11 |
| *CD86* | Up | 0.687870850377447 | 0.000168696594468586 |
| *CDC27* | Up | 0.87438864065984 | 1.38710142156919E-11 |
| *CDCA5* | Down | -0.620961877088058 | 1.00315262567442E-06 |
| *CDH1* | Up | 0.69437289051965 | 0.00164600556116935 |
| *CDK4* | Up | 0.522473866127534 | 0.0000156397199209824 |
| *CDK6* | Up | 1.17178288502211 | 0.000106512721119318 |
| *CDK7* | Up | 0.770740734156389 | 1.46464639819546E-08 |
| *CDKN1A* | Up | 1.19235879985477 | 1.26268711058464E-10 |
| *CDKN2A* | Down | -0.543694103170409 | 0.00383333465970424 |
| *CDO1* | Down | -1.3507695764781 | 0.000191599375023602 |
| *CEBPB* | Up | 0.435078162732562 | 0.000193246309946041 |
| *CENPA* | Up | 0.883423311036133 | 2.48802271850046E-06 |
| *CENPE* | Up | 1.16726920777236 | 3.22656569505573E-14 |
| *CENPJ* | Up | 0.880071027108299 | 8.34842375470038E-10 |
| *CENPK* | Up | 0.783643983394463 | 0.000100953395909044 |
| *CENPO* | Up | 0.420892542725391 | 0.000825687313769684 |
| *CEP135* | Up | 1.19414994881755 | 5.53472047025362E-20 |
| *CEP152* | Up | 1.31563736241309 | 3.77538296461238E-19 |
| *CEP170* | Up | 1.10553898499781 | 1.41060574033002E-14 |
| *CEP192* | Up | 1.05773776491097 | 5.04736798251097E-16 |
| *CEP250* | Up | 0.523819350156442 | 1.91942194381305E-15 |
| *CEP290* | Up | 1.31613795399191 | 1.54111755652759E-29 |
| *CEP55* | Up | 0.639313453501238 | 0.00184077950401776 |
| *CES2* | Down | -1.05434293375978 | 0.0000781448808526583 |
| *CETN2* | Up | 0.477826503503256 | 0.0000671082461609515 |
| *CETN3* | Up | 0.239979289672231 | 0.0367269944673385 |
| *CFTR* | Up | 1.9763178036598 | 7.37073677889019E-21 |
| *CH25H* | Up | 0.976152784675577 | 0.0000129621235859545 |
| *CHD3* | Up | 1.57554412840427 | 1.04143052043829E-32 |
| *CHD4* | Up | 0.659895029304625 | 1.15688367473348E-13 |
| *CHD8* | Up | 0.946459743849978 | 1.21892872294474E-14 |
| *CHST8* | Up | 1.06830316030663 | 9.77204187207377E-30 |
| *CLIC3* | Down | -0.342202941220144 | 0.0101815799110386 |
| *CLNS1A* | Up | 0.763939328684614 | 2.73425268686518E-14 |
| *CLTA* | Up | 0.331960832315051 | 0.000316931733400413 |
| *CNBP* | Down | -0.297677001176302 | 0.0181682361948708 |
| *CNDP1* | Up | 0.717413127122424 | 0.0146172996708234 |
| *CNOT6* | Up | 0.879042985807405 | 8.27710664723993E-14 |
| *COG2* | Up | 0.833047972180198 | 2.91735631592778E-14 |
| *COL4A1* | Up | 1.27919427515681 | 1.72951515477298E-11 |
| *COL4A2* | Up | 1.22178975536193 | 4.83385692794157E-11 |
| *COL4A4* | Up | 1.09106558111872 | 2.71726835089173E-09 |
| *COMT* | Up | 0.439928625698675 | 0.0185006862422336 |
| *COPB1* | Up | 0.741387322119833 | 4.74403072724695E-16 |
| *COPB2* | Up | 0.347637549552953 | 0.0010717306388441 |
| *COPE* | Up | 0.390900487310224 | 0.000258137889425373 |
| *COX5A* | Up | 0.498199417476409 | 1.23829020183522E-07 |
| *CPOX* | Down | -0.531419599334906 | 0.0000435253927509725 |
| *CROCC* | Up | 0.413588081032982 | 0.00467249434615867 |
| *CSAD* | Up | 1.41123345125849 | 6.42169017767949E-13 |
| *CSH1* | Up | 0.333904906697348 | 0.0000314217783889285 |
| *CSNK1A1* | Up | 0.475532072956751 | 2.26404865083585E-06 |
| *CSNK1E* | Up | 1.2684414048161 | 3.67154096082266E-26 |
| *CSTF2* | Up | 0.482270537882266 | 2.43438175890031E-06 |
| *CSTF3* | Up | 0.380634335850266 | 0.0000058755035638388 |
| *CTBP1* | Up | 0.601186210169663 | 9.00915791598975E-12 |
| *CTCF* | Up | 0.385403597196966 | 0.0000822455154394533 |
| *CTNNB1* | Down | -0.712757379802047 | 1.46420299221404E-08 |
| *CXCL12* | Up | 1.61716015822684 | 9.03950703894579E-07 |
| *CXCL14* | Up | 2.37349127803687 | 1.44210403954607E-18 |
| *CYB5A* | Down | -0.580287495793174 | 0.0026548341508817 |
| *CYBRD1* | Up | 0.747717681052547 | 0.00432891267902921 |
| *CYCS* | Up | 0.481300725239432 | 0.0000536881184914982 |
| *CYFIP2* | Up | 1.34062945448185 | 5.54367336264815E-09 |
| *CYP11B1* | Down | -0.736816656679034 | 1.57954722108581E-23 |
| *CYP11B2* | Up | 0.732715401463325 | 2.40960816834088E-14 |
| *CYP1A1* | Down | -1.00682737886738 | 0.00289842212702543 |
| *CYP1B1* | Up | 0.970144087710498 | 0.000139750392528013 |
| *CYP26B1* | Down | -0.760242989036467 | 0.00037345476942993 |
| *CYP27A1* | Down | -0.52103031459357 | 0.0180329463482876 |
| *CYP2A13* | Down | -1.14295422541505 | 0.000282829642076677 |
| *CYP2A7* | Down | -1.27615379681857 | 0.000106258663280203 |
| *CYP2J2* | Down | -0.732911807322811 | 0.00233771187268794 |
| *CYP3A5* | Up | 1.63951540667753 | 8.65299490950499E-12 |
| *CYP46A1* | Down | -0.544393641503125 | 7.57796990537925E-09 |
| *CYP4F2* | Down | -0.969348458023562 | 0.000459629769965295 |
| *DAK* | Up | 0.528347947694916 | 0.00278239992079473 |
| *DBT* | Up | 1.30199760297537 | 2.07432651059503E-22 |
| *DCTN5* | Up | 0.781204276970952 | 4.98257148260696E-14 |
| *DCTN6* | Up | 0.552951686981351 | 0.0000683052666868498 |
| *DDB1* | Up | 0.267984128777982 | 0.00040308750781083 |
| *DDT* | Down | -0.356320382935927 | 0.0282324398216136 |
| *DDX18* | Up | 1.01892887732482 | 1.53623943938863E-21 |
| *DDX21* | Up | 0.927623669596196 | 4.74392819467258E-14 |
| *DEK* | Down | -0.240949535581645 | 0.046687371248318 |
| *DFNA5* | Up | 1.01170110906666 | 1.02002721865292E-06 |
| *DGCR8* | Up | 1.45518685189213 | 6.71237267636048E-39 |
| *DHDH* | Down | -0.211618901252153 | 0.00142282265505147 |
| *DHX15* | Up | 0.809251551208826 | 6.85816373368483E-22 |
| *DHX9* | Up | 1.32235302562028 | 1.0792671173545E-35 |
| *DKK1* | Up | 2.07554723703718 | 0.000024566654887978 |
| *DLAT* | Up | 0.766458173386241 | 1.09256231147699E-06 |
| *DLC1* | Up | 0.777598015865856 | 5.50458332071659E-06 |
| *DLG5* | Up | 0.837329799146868 | 1.83360623669555E-07 |
| *DLL1* | Up | 1.12569464195635 | 7.20054826915833E-07 |
| *DMC1* | Down | -0.721616398709379 | 1.79063761285449E-16 |
| *DNM1L* | Up | 1.32287584151608 | 4.95631009922666E-28 |
| *DOPEY1* | Up | 0.910708369298708 | 4.29316085483902E-13 |
| *DUSP2* | Up | 1.40141472667949 | 1.52142983058561E-29 |
| *DUT* | Up | 0.434370271629772 | 0.0000365270650699488 |
| *DVL2* | Up | 0.901227528368707 | 9.79186871950482E-20 |
| *DZIP1* | Up | 0.778014531979286 | 3.66114047520219E-09 |
| *E2F1* | Up | 1.29697321457795 | 4.5872823674219E-27 |
| *E2F3* | Up | 2.14134376013197 | 1.96332431793515E-29 |
| *EED* | Up | 0.600202695358004 | 6.40572157117718E-07 |
| *EGR2* | Up | 0.728880292552438 | 0.000237178841802047 |
| *EGR3* | Up | 1.11237227394528 | 7.13780207768297E-12 |
| *EHHADH* | Down | -0.815046650016241 | 0.00320882327295117 |
| *EHMT2* | Up | 0.529477250904259 | 0.0000306820943125873 |
| *EID3* | Up | 1.35155042453127 | 3.650103846873E-20 |
| *EIF2S2* | Up | 0.257985515411265 | 0.015510723781506 |
| *EIF4E* | Down | -0.371899513242202 | 0.005689640557202 |
| *ELF3* | Up | 1.47607839110159 | 1.22180631136314E-15 |
| *ELOVL5* | Down | -0.337446555956946 | 0.00496450813035377 |
| *ENO2* | Up | 2.00651636849812 | 1.02112184604292E-21 |
| *ENO3* | Up | 1.2622739478566 | 3.13286626937006E-07 |
| *ENY2* | Up | 0.513028495880084 | 0.0000270469121686938 |
| *EP300* | Up | 1.24201091599438 | 1.97795429160416E-20 |
| *EP400* | Up | 0.805673037708485 | 1.41908262675173E-15 |
| *EPHA2* | Up | 1.17291214104027 | 2.82644207704605E-16 |
| *EPRS* | Up | 1.09088130633191 | 1.18911509936722E-13 |
| *ERCC1* | Up | 0.50377796886674 | 1.16705648357332E-08 |
| *ERCC2* | Up | 0.71324003880243 | 5.0605159643817E-18 |
| *ERCC3* | Up | 1.17177682654983 | 5.2342028733766E-29 |
| *ERCC5* | Up | 0.510827942018908 | 0.0000314276516103891 |
| *ERCC8* | Up | 0.485987992332888 | 0.0000930971888051024 |
| *ERGIC3* | Up | 0.303747861224865 | 0.00129374943411172 |
| *ETF1* | Up | 0.306255621760439 | 0.00445724392551684 |
| *ETS2* | Up | 0.740326617774496 | 3.81457103620778E-06 |
| *EZH1* | Up | 0.931308384577029 | 8.01612231842027E-17 |
| *EZH2* | Up | 1.84128074148513 | 6.68803446051859E-19 |
| *FABP5* | Up | 1.28285996160774 | 4.10185601956015E-09 |
| *FAIM3* | Up | 0.873161703680854 | 1.48102221012998E-10 |
| *FAM48A* | Up | 1.35088401282329 | 1.41527252225494E-31 |
| *FANCD2* | Up | 0.757363872184723 | 1.03700674680098E-08 |
| *FASN* | Up | 1.04367401323046 | 6.61326049921237E-09 |
| *FBL* | Up | 0.757282007317015 | 3.02670576268957E-10 |
| *FCGR3A* | Down | -0.853183903219647 | 0.000290738398457635 |
| *FEN1* | Up | 0.565977651773298 | 0.000149736370879172 |
| *FGFR3* | Up | 1.29723491922559 | 3.06950042733982E-10 |
| *FHL2* | Up | 1.17464676651943 | 3.17892099959594E-07 |
| *FKBP4* | Up | 0.556062940651227 | 4.55692798520785E-08 |
| *FKBP6* | Up | 0.600633716276436 | 2.03785483358017E-18 |
| *FMO1* | Up | 1.98891970574261 | 7.71976256730101E-11 |
| *FOS* | Up | 0.95791095496527 | 0.00111799391014183 |
| *FOXC1* | Up | 1.57343057222547 | 1.83405156336268E-15 |
| *FPGS* | Up | 0.450860997813401 | 2.84099287511928E-07 |
| *FRAT1* | Up | 0.437774575804006 | 0.000121570720136891 |
| *FTL* | Up | 0.480757537408266 | 4.19367599837162E-12 |
| *FZD1* | Up | 1.07046713373743 | 1.39868248673319E-09 |
| *FZD4* | Up | 0.336382295725752 | 0.00809170917350266 |
| *FZD6* | Up | 0.735407229762289 | 0.00721199107751145 |
| *G6PC2* | Down | -0.519037261723826 | 4.0966369836864E-10 |
| *GAA* | Up | 0.408682076579979 | 0.000329140434217185 |
| *GAB2* | Up | 0.824924295518296 | 2.12857834133264E-16 |
| *GAD2* | Up | 0.336085970125854 | 0.0000914945891653863 |
| *GART* | Up | 1.92876965822863 | 1.17715359015934E-27 |
| *GATA3* | Up | 1.21165442825776 | 5.94743710887894E-13 |
| *GATAD2B* | Up | 1.33410206361165 | 4.44375416032448E-27 |
| *GBF1* | Up | 1.32019181943296 | 8.91867671808594E-31 |
| *GCKR* | Up | 0.717360871443047 | 0.0000434085632974862 |
| *GINS2* | Down | -0.506355500485768 | 5.23896534762486E-06 |
| *GJB3* | Up | 0.68373140566985 | 3.8789626753287E-11 |
| *GLA* | Up | 0.662190292441362 | 6.98370649718403E-08 |
| *GNL3* | Up | 0.839009742755779 | 1.94648582656095E-12 |
| *GNPAT* | Up | 0.611950292767546 | 4.79529486126666E-08 |
| *GOSR2* | Up | 0.797947173444659 | 1.37511354247446E-20 |
| *GP1BA* | Up | 0.690461846260709 | 6.22447208972936E-24 |
| *GPI* | Up | 0.395882389313 | 0.00325986200198363 |
| *GSTA2* | Down | -0.715238067619916 | 0.000646146331355609 |
| *GSTA3* | Down | -0.665264715901137 | 0.000444103231451625 |
| *GSTM1* | Up | 0.521936803858144 | 0.0225063753529825 |
| *GSTM5* | Up | 0.356271608544904 | 0.000468004878054461 |
| *GSTP1* | Up | 1.41645991701628 | 8.43506345884158E-17 |
| *GSTT2* | Down | -0.870435607548151 | 0.0000302855489313594 |
| *GTF2A2* | Up | 0.372325420405482 | 0.0012766300862812 |
| *GTF2F2* | Down | -0.739169190618639 | 1.02579142135554E-06 |
| *GTF2H1* | Up | 1.02451904420824 | 2.84244324476085E-22 |
| *GTF2H2* | Up | 0.643262009551545 | 1.25064292038985E-06 |
| *GTF2I* | Up | 0.561147349133874 | 0.0000506284171129855 |
| *GTF2IRD1* | Up | 0.944732672629745 | 7.45459266426505E-16 |
| *GTF3C5* | Up | 0.894880970007293 | 3.55116802399615E-22 |
| *GYS1* | Up | 1.12265607409968 | 5.90166586496365E-21 |
| *H1F0* | Down | -0.667442480810365 | 0.0000178325240951385 |
| *H1FOO* | Up | 0.432438883527865 | 3.48763856810189E-14 |
| *H2AFX* | Up | 1.39564547685848 | 5.70643692955662E-16 |
| *H2AFY* | Up | 0.83450720645236 | 9.37897881913978E-16 |
| *H2AFY2* | Up | 1.41035663758445 | 5.27210543250445E-18 |
| *H2BFM* | Up | 1.24403413374287 | 7.07598238836501E-43 |
| *HADH* | Down | -0.47185842276734 | 0.00438476245576573 |
| *HADHA* | Down | -0.643875428858152 | 2.35783437997425E-06 |
| *HADHB* | Down | -0.278916355525469 | 0.00834164690935596 |
| *HAO1* | Down | -1.44950576947752 | 0.0000500693214179237 |
| *HAP1* | Down | -0.260868557525913 | 0.000957408240235356 |
| *HCLS1* | Up | 1.3485647192782 | 5.37370635695524E-11 |
| *HDAC1* | Up | 0.566900256878769 | 5.83970099103939E-08 |
| *HDAC5* | Up | 0.65793557874689 | 7.96231981878936E-10 |
| *HDGF* | Up | 0.849351261251401 | 3.06436859639794E-19 |
| *HERC2* | Up | 0.689007100139885 | 5.40718614744406E-11 |
| *HES6* | Down | -0.519901242805843 | 0.0000434085632974862 |
| *HEY1* | Up | 1.00069169467668 | 7.52117507051227E-10 |
| *HGFAC* | Up | 1.11932506761854 | 0.000266366574911982 |
| *HIBADH* | Down | -1.50827605335954 | 3.21839898955777E-14 |
| *HILS1* | Up | 0.725229213682526 | 8.8841797001442E-18 |
| *HIRA* | Down | -1.07964069073508 | 1.8462586057229E-07 |
| *HIST1H1B* | Down | -0.413851815713683 | 0.0000263278226904287 |
| *HIST1H1E* | Up | 1.24969958146149 | 3.27757808075309E-28 |
| *HIST1H2AB* | Up | 0.503890499591182 | 2.11843909939316E-07 |
| *HIST1H2AD* | Down | -0.201824393137507 | 0.0219206265722268 |
| *HIST1H2AK* | Up | 1.06375515212303 | 3.5292547210585E-25 |
| *HIST1H2AL* | Up | 0.685574456089099 | 1.28915253581266E-30 |
| *HIST1H2BA* | Up | 0.536537170942845 | 1.45105934470911E-25 |
| *HIST1H2BB* | Up | 0.792788034412645 | 3.73498800834958E-24 |
| *HIST1H2BF* | Down | -0.400010401949221 | 0.0262583596517862 |
| *HIST1H2BI* | Down | -0.487890050650015 | 0.00263441759274374 |
| *HIST1H2BJ* | Up | 0.779659282686709 | 4.04834483894683E-28 |
| *HIST1H2BL* | Up | 1.77908544477911 | 9.32703008395321E-49 |
| *HIST1H2BM* | Up | 0.416298092507329 | 1.88348390565211E-08 |
| *HIST1H2BN* | Up | 0.853095025477277 | 3.19309829478395E-13 |
| *HIST1H2BO* | Up | 1.03575709009794 | 9.31379363314757E-36 |
| *HIST1H3A* | Up | 0.232709100956296 | 0.0000811179398547968 |
| *HIST1H3C* | Up | 0.930791981949908 | 9.025976498465E-27 |
| *HIST1H3D* | Up | 0.488984806919474 | 8.29929306572072E-06 |
| *HIST1H3E* | Up | 1.33138063656173 | 1.00878780026713E-20 |
| *HIST1H3F* | Up | 0.563308103336868 | 1.27838735542715E-21 |
| *HIST1H3G* | Up | 0.826441155829561 | 5.47291516332676E-16 |
| *HIST1H3I* | Up | 0.739144629635454 | 6.98666898146588E-27 |
| *HIST1H3J* | Up | 1.33276800064565 | 1.24885799890338E-29 |
| *HIST1H4A* | Up | 0.990942635793234 | 2.15457669057334E-29 |
| *HIST1H4B* | Up | 0.388081116610825 | 1.72551507598549E-11 |
| *HIST1H4C* | Up | 0.654900707957081 | 6.82503690452545E-06 |
| *HIST1H4D* | Up | 0.803221772499706 | 2.9593239958342E-23 |
| *HIST1H4E* | Up | 0.879727584019815 | 3.71053866232933E-15 |
| *HIST1H4F* | Up | 0.643027510515151 | 3.09282904931114E-24 |
| *HIST1H4J* | Up | 0.502503804593768 | 0.000206930611481863 |
| *HIST1H4K* | Up | 0.502503804593768 | 0.000206930611481863 |
| *HIST1H4L* | Down | -0.683330724502922 | 3.3349416776901E-11 |
| *HIST2H2AA3* | Up | 0.663085622196131 | 2.16045121265136E-10 |
| *HIST2H2BE* | Up | 0.74519197400194 | 0.000107093990488587 |
| *HIST3H2A* | Up | 1.2835691296433 | 6.66451854310737E-17 |
| *HIST3H3* | Up | 0.56398620529034 | 6.06913692089792E-14 |
| *HK3* | Up | 0.800473034007193 | 2.69596433050894E-13 |
| *HLA-DOA* | Up | 0.926360497401324 | 1.15833661055582E-18 |
| *HLA-DQB1* | Up | 0.635809653735163 | 0.00192291861625057 |
| *HLA-DRB1* | Up | 0.712214182942066 | 0.000789255277133407 |
| *HLA-DRB4* | Up | 0.990905377643454 | 0.000123935630914337 |
| *HMBS* | Up | 0.343571469693808 | 8.17480868901094E-06 |
| *HMGA1* | Up | 1.17280996062644 | 5.41060131325124E-13 |
| *HMGB2* | Up | 1.14513621656873 | 9.98804625939416E-11 |
| *HMGCS2* | Down | -1.3590881540664 | 9.54609036145127E-06 |
| *HOMER2* | Up | 0.653999998061439 | 0.00378573732765137 |
| *HORMAD1* | Up | 0.451504446882419 | 1.54449128239856E-09 |
| *HORMAD2* | Down | -0.55155471236048 | 0.000015261825328719 |
| *HOXA1* | Up | 0.537113428186587 | 4.03101540607056E-07 |
| *HOXA2* | Up | 1.20413991036705 | 6.46057617311161E-31 |
| *HOXA3* | Up | 1.14036510729229 | 6.99732490295346E-41 |
| *HOXB1* | Up | 0.810451168994199 | 4.55550870241224E-17 |
| *HOXB2* | Up | 1.28446151765527 | 8.05858042357743E-19 |
| *HPRT1* | Down | -0.759218302148948 | 6.43850208929493E-06 |
| *HSD11B1* | Down | -1.23468592118772 | 0.0148000499233342 |
| *HSD11B2* | Up | 0.458648326023879 | 0.000296424240682538 |
| *HSD17B4* | Down | -0.431811704687409 | 0.0112771184335965 |
| *HSD17B6* | Down | -1.72333941789955 | 4.40446461800813E-06 |
| *HSD17B7* | Up | 0.840729810672926 | 4.63245826828954E-13 |
| *HSP90AA1* | Up | 0.363211623335591 | 0.0000112391524345404 |
| *HSP90AB1* | Up | 0.440084023236924 | 0.00015223781739472 |
| *HSPH1* | Up | 0.459399890024716 | 0.004817632585003 |
| *IARS* | Up | 0.646606580652765 | 1.44018190444744E-08 |
| *ICA1* | Up | 1.47216352595484 | 1.86306230342187E-17 |
| *ID2* | Up | 0.457441639113913 | 0.00561970108628277 |
| *IDH3B* | Up | 0.2241613826967 | 0.0254069838054553 |
| *IFRD1* | Up | 1.13276151634691 | 1.38739579949768E-12 |
| *IFT52* | Up | 0.610376397074871 | 4.01905209214904E-10 |
| *IGF1R* | Up | 1.92351109775645 | 2.47339257839146E-40 |
| *IGFBP4* | Up | 0.724959755060766 | 1.41121784792456E-06 |
| *IKBKB* | Up | 1.41144245128211 | 1.42986295289375E-21 |
| *IL10* | Up | 0.488415311728349 | 9.61457857615157E-08 |
| *IL19* | Up | 0.551231698957775 | 2.50302421924318E-13 |
| *IL20* | Up | 0.535960982586446 | 8.49399859493988E-09 |
| *IL22* | Down | -0.139537396036189 | 0.037730783095867 |
| *IL24* | Up | 0.152212649989362 | 0.00857478088564438 |
| *IL6* | Up | 1.80377407215279 | 5.63142975632411E-25 |
| *INCENP* | Up | 0.539286712670724 | 2.91630058845192E-07 |
| *INMT* | Up | 1.29796915873451 | 6.9725628265738E-09 |
| *INPP5F* | Up | 0.975945347417953 | 3.06821081576416E-10 |
| *IREB2* | Up | 0.320484319908821 | 0.0140226493452695 |
| *IRF7* | Up | 1.09336714872357 | 1.28480052223525E-09 |
| *ISG20L2* | Up | 0.371820274021697 | 0.00154258667646186 |
| *ISOC1* | Down | -0.55925343876301 | 0.000489737619046412 |
| *ITCH* | Down | -0.355294463690659 | 0.00115207375830497 |
| *ITGA2* | Up | 1.02805654868773 | 7.84130555988921E-11 |
| *ITGA2B* | Down | -0.651827572248588 | 4.50314809541008E-19 |
| *ITGAV* | Up | 0.852149571264842 | 0.0000141328195352774 |
| *ITGB3BP* | Up | 0.501743707573493 | 0.000224338385660605 |
| *ITIH1* | Down | -0.783227143214635 | 0.000963407215170781 |
| *ITPA* | Up | 0.576090791552547 | 3.07004372070434E-11 |
| *ITPK1* | Down | -0.362822157143172 | 0.00882012208811842 |
| *JAG1* | Up | 1.25224529995607 | 9.93527286224787E-11 |
| *JARID2* | Up | 0.960544325217937 | 1.88854780409401E-23 |
| *KCNK15* | Down | -0.649163151332287 | 6.10860378955555E-12 |
| *KIAA1009* | Up | 0.510536177319987 | 4.97823359605989E-07 |
| *KIAA1731* | Up | 1.28442664685062 | 1.86853264579566E-28 |
| *KIF20A* | Up | 1.02160912237223 | 5.8504044767612E-07 |
| *KIF24* | Up | 0.939340633755177 | 1.68365147152226E-38 |
| *KIF2B* | Up | 0.595719572004963 | 3.77169568230732E-25 |
| *KIFAP3* | Up | 0.597103264745106 | 0.0000419974578559351 |
| *KLF1* | Up | 0.160668959883633 | 0.00972033733171124 |
| *KLF10* | Up | 0.714449084987661 | 3.47037624147124E-06 |
| *KLF4* | Up | 0.781335521575668 | 0.00165358126658665 |
| *KLK10* | Up | 0.733842851539687 | 5.3077155296779E-16 |
| *KLK11* | Up | 0.250905922814104 | 0.0187304283383778 |
| *KNTC1* | Up | 1.74874342231301 | 1.50240678890128E-18 |
| *KPNA2* | Up | 0.835991403672601 | 3.28306129480534E-09 |
| *KPNB1* | Up | 0.518240908231734 | 1.73496041797421E-07 |
| *KREMEN2* | Up | 0.542063248762201 | 1.03632838433374E-13 |
| *KRT13* | Up | 0.144201955345772 | 0.0221403645243792 |
| *KRT15* | Up | 0.355924485574378 | 1.23155575423263E-08 |
| *LAD1* | Up | 1.25734570859479 | 3.35409224439103E-11 |
| *LAMA2* | Up | 1.28723576681728 | 2.37651397426655E-10 |
| *LAMA4* | Up | 1.07934874875339 | 1.63582179335492E-10 |
| *LAMB4* | Up | 0.646037715179117 | 1.055783805814E-22 |
| *LAMC1* | Up | 0.940298225337884 | 2.7971070779289E-07 |
| *LAMC3* | Up | 1.5544871033023 | 1.41657839069974E-21 |
| *LARGE* | Up | 0.45302107806539 | 0.00005107442447355 |
| *LCAT* | Up | 1.29045513663904 | 2.57152959178874E-08 |
| *LCK* | Up | 1.38577390807779 | 4.90110668894447E-14 |
| *LDB1* | Up | 1.22784278475905 | 2.6750811152165E-37 |
| *LEF1* | Up | 1.15004557381949 | 0.0000676856522510476 |
| *LIG1* | Up | 0.739158587360262 | 4.80477353689925E-10 |
| *LIPE* | Up | 1.13886235261417 | 1.06065145764645E-25 |
| *LLGL2* | Up | 0.675962204667834 | 3.25354039902299E-08 |
| *LMNB1* | Down | -1.11052807718066 | 2.07629899276086E-08 |
| *LMO2* | Up | 0.584465503060996 | 0.0000307769463638012 |
| *LRPPRC* | Up | 0.534619543756584 | 5.89405579064582E-08 |
| *LSM7* | Up | 0.49375472896652 | 8.87208956568121E-06 |
| *LTC4S* | Up | 0.913299488095901 | 3.42535649807994E-30 |
| *LTF* | Up | 0.83662102981241 | 2.57930007776657E-14 |
| *MAF1* | Up | 0.859633447398652 | 2.60283611688708E-11 |
| *MAML1* | Up | 0.701536787306877 | 3.5138701818551E-14 |
| *MAN1A1* | Down | -0.484663337156601 | 0.0193445428959012 |
| *MAPK1* | Up | 0.687382918187383 | 9.76753025939241E-10 |
| *MAPK13* | Up | 2.25446314616111 | 4.86052416153283E-23 |
| *MAPK3* | Up | 1.13860019329897 | 1.08253963262047E-39 |
| *MAT2A* | Up | 0.629786065645265 | 1.36448865130649E-10 |
| *MCCC1* | Down | -0.836327872294881 | 7.17809409981822E-09 |
| *MCCC2* | Down | -0.407971496578813 | 0.0159888771615339 |
| *MCEE* | Down | -0.684265596384013 | 0.0000507706104887819 |
| *MCM3* | Up | 0.550392081937304 | 0.000268801754944314 |
| *MCM4* | Up | 0.753152621666985 | 0.0000205696182592109 |
| *MCM5* | Up | 1.09744888025418 | 7.83469815820124E-12 |
| *MCM6* | Up | 0.674934999866327 | 0.0000259761966792499 |
| *MCM7* | Up | 0.576741452308168 | 4.40892991119994E-06 |
| *MCM8* | Up | 1.31029046486834 | 1.38929663275237E-13 |
| *MDM1* | Up | 0.92059625201134 | 6.74382045440732E-10 |
| *METAP1* | Up | 0.487957635765734 | 0.000037586584267912 |
| *METTL3* | Up | 1.09709611564307 | 1.18844226862762E-22 |
| *MGST1* | Down | -0.691114224889391 | 0.0118287456565373 |
| *MIS12* | Up | 0.812726332368234 | 1.91506995997147E-08 |
| *MKI67* | Up | 0.821536479375926 | 5.78045560066677E-08 |
| *MKI67IP* | Up | 0.857481540165916 | 6.17161218804829E-12 |
| *MKS1* | Up | 1.13259509672555 | 1.87073392835684E-27 |
| *MLH1* | Up | 0.561572811973787 | 5.71165186121568E-11 |
| *MLH3* | Up | 0.914044388013158 | 2.50302421924318E-13 |
| *MPG* | Up | 0.560636907353397 | 5.61869158068784E-11 |
| *MPHOSPH9* | Up | 0.743694432756179 | 1.77365411054341E-08 |
| *MPP2* | Up | 0.868314718945163 | 2.03047382347462E-18 |
| *MRE11A* | Up | 0.971423692989953 | 4.67457459294246E-16 |
| *MRPL9* | Up | 0.479382013490385 | 1.02401380276142E-06 |
| *MSH5* | Up | 1.2882732621617 | 5.99171822570137E-15 |
| *MT2A* | Up | 0.855368109481415 | 0.00349282555734028 |
| *MTA2* | Up | 0.578938358544405 | 3.72821363057551E-09 |
| *MTA3* | Up | 0.755263704722385 | 5.143591033175E-11 |
| *MTF2* | Up | 1.01423136717909 | 3.55483678091115E-15 |
| *MTR* | Up | 0.996394104750405 | 1.41561197320121E-11 |
| *MTRR* | Up | 0.762464374492301 | 2.67023246240625E-11 |
| *MYBL1* | Up | 0.737121420003272 | 0.0000211830364791714 |
| *NAB2* | Up | 1.06762486185515 | 5.74519997747406E-22 |
| *NAP1L1* | Up | 0.686522593642859 | 1.040804205538E-08 |
| *NBL1* | Up | 0.936149035830783 | 9.49235699711945E-12 |
| *NBN* | Up | 0.467107953856554 | 0.00306172336081391 |
| *NCAPD2* | Up | 1.04587934886121 | 7.5282512618767E-13 |
| *NCAPD3* | Up | 0.613100722794185 | 3.9249534870329E-09 |
| *NCAPH* | Up | 0.656790781433604 | 3.81350430290891E-08 |
| *NCBP2* | Up | 0.683026705979056 | 4.01418897126096E-10 |
| *NCK2* | Up | 1.49415425928669 | 6.87612273804478E-16 |
| *NCOA2* | Up | 1.98442657615322 | 1.69486655249392E-29 |
| *NCOA6* | Up | 0.748298126242998 | 4.53337287218244E-13 |
| *NCSTN* | Up | 0.553629157334891 | 2.71330995725023E-07 |
| *NDEL1* | Up | 0.92286995186609 | 2.26833483233749E-16 |
| *NEDD1* | Up | 0.462064228013243 | 0.0000175299477499583 |
| *NFE2* | Up | 0.243890004043077 | 0.00803927922587357 |
| *NFKBIA* | Up | 1.02569927635772 | 1.62062515747846E-13 |
| *NFKBIB* | Down | -0.623400020031744 | 2.26187099189168E-11 |
| *NFS1* | Up | 0.682599676908089 | 2.7820235875286E-12 |
| *NKIRAS2* | Up | 0.653186559106225 | 7.03144447644332E-15 |
| *NMT1* | Up | 0.444041423278183 | 1.35972280202787E-07 |
| *NOL6* | Up | 1.00463317191824 | 1.77068471072011E-24 |
| *NOLC1* | Up | 0.691038006714008 | 1.75065311678697E-11 |
| *NPC1* | Up | 0.638228436947233 | 0.0000071051725748544 |
| *NPY1R* | Up | 2.05200059692114 | 2.00997298721736E-19 |
| *NR2E1* | Up | 0.495861438776042 | 6.47678836694384E-10 |
| *NR4A3* | Up | 1.85403236328677 | 8.18925064384841E-29 |
| *NRG1* | Up | 1.38917682526318 | 1.94198654186739E-24 |
| *NT5C* | Up | 0.512503486080019 | 7.65852352272293E-11 |
| *NUBP2* | Up | 0.872808159410886 | 1.44551673796411E-24 |
| *NUP107* | Up | 0.678450408893197 | 1.87597178494232E-10 |
| *NUP133* | Up | 0.789515993959211 | 2.58136458862844E-09 |
| *NUP153* | Up | 0.653289833932417 | 1.01367367588006E-07 |
| *NUP160* | Up | 0.699783099220578 | 4.08652880670167E-07 |
| *NUP188* | Up | 0.621239498597451 | 4.19087805102186E-12 |
| *NUP205* | Up | 0.841773236143325 | 8.53296410295575E-11 |
| *NUP210* | Up | 0.778456238769464 | 1.03723992142765E-10 |
| *NUP214* | Up | 0.745547410072946 | 7.60013900246258E-18 |
| *NUP35* | Up | 0.615876224492258 | 1.11871193413148E-06 |
| *NUP43* | Up | 0.875145977057501 | 1.39252575758573E-13 |
| *NUP50* | Up | 0.977606802796988 | 5.36456937748181E-19 |
| *NUP62* | Up | 1.19975791900465 | 7.41797729034726E-24 |
| *NUP85* | Up | 0.634023989080045 | 3.13974177396413E-09 |
| *NUP93* | Up | 0.903479955388018 | 6.72585606846776E-16 |
| *NUP98* | Up | 1.28203065809043 | 3.95915032357436E-33 |
| *NUPL2* | Up | 0.594461047006936 | 1.98367159243231E-09 |
| *OFD1* | Up | 1.17855807579343 | 2.63439980891454E-30 |
| *OLFM1* | Down | -0.232060205754017 | 0.00232538404742518 |
| *OLFML3* | Up | 1.26467296279909 | 2.23116593234828E-07 |
| *OPTN* | Up | 0.321282490468567 | 0.00902878040070146 |
| *OXCT2* | Up | 0.93430390425091 | 1.30939373313688E-30 |
| *PA2G4* | Up | 1.05916128714108 | 1.86023004195561E-28 |
| *PABPC1* | Up | 0.42160560872024 | 0.00033615352557057 |
| *PAK1* | Up | 0.974667077136334 | 4.34833694715659E-19 |
| *PAK2* | Up | 1.01899196073901 | 6.56399577746571E-22 |
| *PAK6* | Up | 0.954910256729899 | 2.36973658921205E-15 |
| *PAOX* | Up | 0.411577351433014 | 0.00498132868366065 |
| *PAPSS1* | Up | 0.76870158243855 | 4.84514808419051E-07 |
| *PARP3* | Up | 0.298965668612484 | 0.0137958317447896 |
| *PAX6* | Down | -0.331498738964799 | 0.000692390843722727 |
| *PAXIP1* | Up | 0.822760974428704 | 3.67143187205112E-13 |
| *PCBD1* | Down | -0.417634767739056 | 0.00914052823669513 |
| *PCBP1* | Down | -0.274770732061945 | 0.0455801552891072 |
| *PCGF2* | Up | 0.646126989530345 | 3.4504141373356E-07 |
| *PCNT* | Up | 0.648381775980017 | 4.63098275031203E-15 |
| *PDCD4* | Up | 1.42926648394186 | 2.95538587142303E-23 |
| *PDE4B* | Up | 0.882813131377991 | 0.0000266137860772751 |
| *PDK2* | Up | 0.42252871501199 | 6.98663615828535E-06 |
| *PECR* | Up | 0.608476846378315 | 4.52132558800823E-08 |
| *PES1* | Up | 0.803893458658613 | 5.97005764634958E-17 |
| *PEX11G* | Up | 0.937397977722512 | 7.67759501682978E-17 |
| *PEX13* | Down | -0.44351011681064 | 0.000550742530589967 |
| *PEX16* | Up | 0.32343389570668 | 0.000188648250810944 |
| *PEX26* | Up | 0.685786574655517 | 4.23523237037421E-11 |
| *PF4* | Up | 0.987720302460706 | 3.7578640965227E-23 |
| *PGM1* | Down | -0.900497081648615 | 1.90348148265391E-08 |
| *PGRMC1* | Down | -0.629497209013843 | 0.00002085723695421 |
| *PHC2* | Up | 1.12131893376059 | 9.40462329811954E-26 |
| *PHC3* | Up | 0.530929143728208 | 3.04791320501222E-06 |
| *PHF1* | Up | 0.318247972365256 | 0.00141269139228443 |
| *PHF6* | Up | 1.15856255217334 | 5.04674803201503E-24 |
| *PHYH* | Down | -0.75321460735017 | 0.0000150286855530033 |
| *PIAS1* | Up | 0.662845013009891 | 8.18927914769555E-13 |
| *PIAS2* | Up | 0.732216917321239 | 9.48605984682411E-08 |
| *PIAS3* | Up | 1.14231468595046 | 7.87293372138134E-24 |
| *PIAS4* | Up | 0.566635865240285 | 3.65905705632392E-08 |
| *PIK3CB* | Up | 0.676679529299017 | 1.38138352934867E-08 |
| *PIK3CD* | Up | 0.775841223737444 | 5.01321626595008E-08 |
| *PIK3R1* | Down | -0.861823841835884 | 0.0000123784154880084 |
| *PIK3R2* | Up | 0.654493367520837 | 5.09337194143576E-10 |
| *PIK3R5* | Up | 0.825563438022893 | 2.90799023819897E-10 |
| *PINK1* | Up | 0.299994250682391 | 0.0213289217467965 |
| *PKNOX1* | Up | 1.39859313007091 | 8.58579213071982E-39 |
| *PLA2G3* | Down | -0.594462512627604 | 3.52525761355457E-06 |
| *PLAC1* | Up | 1.10899457093707 | 5.68894945294718E-17 |
| *PLG* | Down | -1.19414270356224 | 0.0000985903436694361 |
| *PLK1* | Up | 0.409757957773046 | 3.12970610497624E-12 |
| *PLK2* | Up | 0.767017644671609 | 0.0000103971716473591 |
| *PLK4* | Up | 1.13400490593715 | 1.61540233522399E-19 |
| *PMS1* | Up | 1.3038614889591 | 1.18943754497323E-26 |
| *PODXL* | Up | 0.709884416784625 | 0.0000142043982524908 |
| *POLA1* | Up | 0.755027539056136 | 5.73228019131137E-09 |
| *POLA2* | Up | 0.572723199460818 | 1.59491664616946E-09 |
| *POLD3* | Up | 1.54532432539434 | 2.25473274683656E-28 |
| *POLD4* | Up | 0.545142550910031 | 0.0000116945914709678 |
| *POLH* | Up | 0.859323051777831 | 1.53369543760004E-15 |
| *POLR1C* | Up | 0.973018014391766 | 3.62477817636253E-17 |
| *POLR2B* | Up | 0.961230205601758 | 6.02832081395061E-24 |
| *POLR2D* | Up | 0.557563934087056 | 3.21633278271897E-08 |
| *POLR2G* | Up | 0.310751979911331 | 0.000455324101899756 |
| *POLR2H* | Up | 1.00358135981055 | 3.47563360125096E-27 |
| *POLR2I* | Up | 0.289141068837488 | 0.00104458589194965 |
| *POLR3C* | Up | 0.806705237531268 | 1.36632508318187E-10 |
| *POM121* | Up | 1.07629845131562 | 1.69115137138009E-25 |
| *POR* | Up | 0.680725214041038 | 7.29400931109341E-06 |
| *PORCN* | Up | 0.718099753195643 | 3.34578679732999E-11 |
| *PPARD* | Up | 1.05322836521384 | 2.80201791681103E-23 |
| *PPIA* | Up | 0.537114806840902 | 1.92473233445863E-13 |
| *PPM1A* | Down | -0.492946094155233 | 5.91260904469919E-06 |
| *PPM1K* | Up | 0.662276531332395 | 0.00174392857430812 |
| *PPP2CA* | Up | 0.248137725293208 | 0.0401030468011954 |
| *PPP2R1A* | Up | 0.312627700780402 | 0.00239100357806769 |
| *PPP2R1B* | Up | 0.633165673525692 | 0.0000471154583366924 |
| *PPP2R5A* | Up | 0.429601722240186 | 0.000423478361164481 |
| *PPP2R5D* | Up | 1.03964895348675 | 4.34899075340758E-27 |
| *PRDX3* | Down | -0.332647032252108 | 0.0098167138122041 |
| *PRDX4* | Down | -0.555733270421538 | 0.0000171091403934305 |
| *PRKAA2* | Up | 1.75808056715498 | 4.51411809813361E-23 |
| *PRKAB1* | Up | 0.450012270335884 | 0.0000269482976386339 |
| *PRKAG1* | Up | 0.302968252802787 | 0.00149945516257165 |
| *PRKAG3* | Up | 0.899815701424846 | 5.92153796325683E-25 |
| *PRKAR2B* | Up | 1.18045689447089 | 3.02154658436581E-09 |
| *PRKCQ* | Up | 1.39475742126294 | 9.2594841022877E-25 |
| *PRL* | Up | 0.478837373684391 | 2.37848831222782E-06 |
| *PROS1* | Down | -0.512855035843977 | 0.0206992444924798 |
| *PRPF31* | Up | 0.716255080961824 | 2.7334099047834E-13 |
| *PSMA2* | Down | -0.450495984670995 | 0.000226970441981628 |
| *PSMA4* | Up | 0.656306343396833 | 4.02252329258929E-15 |
| *PSMA6* | Up | 0.351302919361732 | 0.0000865738223313184 |
| *PSMA8* | Up | 0.473529645756186 | 4.98751667639628E-10 |
| *PSMB1* | Up | 0.479193190847484 | 1.21720478552114E-07 |
| *PSMC2* | Up | 0.579350520674279 | 1.77585170646297E-09 |
| *PSMC4* | Up | 0.707568192208198 | 3.68354709024725E-12 |
| *PSMD10* | Up | 0.385031825008991 | 0.00638963459331888 |
| *PSMD11* | Up | 0.375971023180865 | 0.000988062184213967 |
| *PSMD2* | Up | 0.577256199936867 | 2.04120862164643E-10 |
| *PSMD4* | Up | 0.287253316500639 | 0.00744150003204973 |
| *PSMD5* | Up | 0.46671246777511 | 0.0000409336463991067 |
| *PSME3* | Up | 0.665431750326485 | 5.42211506044074E-10 |
| *PTGS2* | Up | 2.18290349478263 | 1.68102715653757E-21 |
| *PTPN1* | Up | 1.33007364755685 | 4.86937075554663E-38 |
| *PTPN6* | Up | 0.976783726756158 | 8.94010026160053E-18 |
| *PTPRG* | Up | 0.696393633685845 | 4.18817718741705E-07 |
| *PTS* | Up | 0.637570926591985 | 1.19057521066703E-06 |
| *PWP1* | Up | 1.10372047101825 | 4.50616539171157E-22 |
| *PXMP2* | Down | -0.92908538615072 | 1.61149885533296E-07 |
| *PYGB* | Up | 0.394679293461734 | 0.00195547544440731 |
| *PYGL* | Down | -0.437820697019491 | 0.0107281630880701 |
| *PYGM* | Up | 0.871265896886476 | 1.0255994870832E-22 |
| *PYGO1* | Up | 0.525171681199071 | 2.30886864942245E-22 |
| *PYGO2* | Up | 0.964415748979967 | 8.37794320043719E-21 |
| *RAB5A* | Up | 0.676197574694102 | 6.97489743343433E-11 |
| *RAC1* | Up | 0.395087344908024 | 0.0000763128635172971 |
| *RAD1* | Up | 1.00485417733767 | 2.68212246658011E-16 |
| *RAD51* | Up | 0.32404287363131 | 0.00120128426386079 |
| *RAD52* | Up | 1.27880693433662 | 4.3126328355717E-22 |
| *RAD9A* | Up | 0.721916682263019 | 7.18333701339512E-12 |
| *RAD9B* | Down | -0.204753313849881 | 0.0000695974455788872 |
| *RAE1* | Up | 0.879660515626422 | 2.38418558950966E-17 |
| *RAN* | Up | 0.439521015044932 | 0.00210272164456848 |
| *RANGAP1* | Up | 0.957889647049372 | 5.21086158857255E-17 |
| *RARB* | Up | 0.574298453722577 | 0.000121335938204263 |
| *RBBP7* | Up | 0.234802785539365 | 0.0192988149613178 |
| *RBP1* | Up | 1.40967752980297 | 0.0000108647330685894 |
| *RBX1* | Up | 0.497261071848015 | 4.15205696401263E-07 |
| *RDH10* | Down | -0.467937156895073 | 0.0188852585194913 |
| *REEP1* | Up | 1.18942535671875 | 2.34263628572155E-07 |
| *REST* | Up | 1.98537188594185 | 2.76292380434038E-50 |
| *RET* | Up | 0.284432428289148 | 0.00152273976917085 |
| *RETSAT* | Down | -0.694426257978498 | 3.33576693665787E-06 |
| *RFC4* | Up | 0.595136647869169 | 0.000109326317135055 |
| *RGS12* | Up | 0.754804331651751 | 3.59704537254149E-11 |
| *RHOD* | Up | 0.617022295398014 | 1.1521150450299E-10 |
| *RIPK1* | Up | 0.837163648324003 | 3.57683876576374E-18 |
| *RIPK3* | Up | 0.81857814262946 | 3.73995683852302E-12 |
| *RNASEH2A* | Up | 0.443339516533431 | 0.00366753353900596 |
| *RNF2* | Up | 0.686693528642521 | 1.19410508546311E-13 |
| *RNF43* | Up | 0.737266140611523 | 0.000120152131065395 |
| *RNMT* | Up | 0.848971609194194 | 9.74822156418335E-20 |
| *ROBO1* | Up | 1.41940975507999 | 3.67226110197603E-06 |
| *ROCK1* | Up | 0.468139268297948 | 1.72420984939772E-14 |
| *RPL14* | Up | 0.560597507693949 | 9.74692450933965E-11 |
| *RPLP0* | Up | 0.324539656501681 | 0.0000439639241573928 |
| *RPS10* | Up | 0.835041632784305 | 6.96355032889185E-20 |
| *RPS2* | Up | 0.413741230788135 | 0.0000170860891596896 |
| *RPS27A* | Up | 0.305236628919211 | 0.0000580425964129145 |
| *RPS3* | Up | 1.14169771953293 | 9.07160165940617E-22 |
| *RPS6KA1* | Up | 1.10980506063819 | 8.65348016962359E-16 |
| *RPS6KB1* | Up | 0.541669078392733 | 2.59884697012249E-06 |
| *RRAGB* | Up | 0.405973814342515 | 0.0000124581916447231 |
| *RRAGC* | Up | 1.1910811018318 | 2.91972549091751E-21 |
| *RRM1* | Up | 0.470244634482995 | 0.000144124456143328 |
| *RRS1* | Up | 0.786764336457249 | 8.13076444987207E-09 |
| *RSL1D1* | Up | 0.56463878564403 | 2.21188282418212E-06 |
| *RUNX1* | Up | 1.32821323231073 | 1.71977600532862E-16 |
| *RUNX3* | Up | 1.20410631784771 | 4.77389367034401E-31 |
| *RUVBL2* | Up | 0.457189583818923 | 6.41438303620537E-06 |
| *RXRB* | Up | 1.13905409024223 | 2.0964779164147E-32 |
| *RXRG* | Up | 0.984708051439673 | 6.53995528798535E-23 |
| *S100A10* | Up | 1.10319626179776 | 1.86978415978329E-07 |
| *S100A12* | Up | 0.397031648310302 | 0.00102106986063168 |
| *SAC3D1* | Up | 0.597151755681488 | 0.0000809055219839872 |
| *SALL4* | Up | 0.685009953543285 | 0.000206960367346136 |
| *SAR1B* | Down | -0.567560758129423 | 0.00107055205082319 |
| *SCAMP1* | Up | 0.39018663846428 | 0.000141089954143212 |
| *SCAMP3* | Up | 0.368309655128826 | 0.000995661644622139 |
| *SCN11A* | Up | 0.685670564514909 | 1.15873402077103E-23 |
| *SCN1A* | Up | 1.1605857524948 | 2.85297869965714E-32 |
| *SCN2B* | Up | 0.61462389645908 | 6.13666781571128E-19 |
| *SCN3B* | Up | 0.815311129604432 | 4.31698089275646E-19 |
| *SCN7A* | Up | 1.26149090199218 | 3.92476380803837E-34 |
| *SCNN1A* | Up | 0.326307819086709 | 0.000159528218717875 |
| *SCNN1G* | Up | 0.807018126300767 | 4.25191996932817E-29 |
| *SDCCAG8* | Up | 1.55504584580692 | 1.58169724233805E-19 |
| *SDHD* | Down | -0.867952250056666 | 2.03290636000119E-06 |
| *SEC24D* | Down | -0.739004366625974 | 0.0001480784479825 |
| *SEC31A* | Up | 0.514192152090422 | 0.0000238592319112443 |
| *SEC61A1* | Up | 0.640617136350349 | 1.33036128412486E-10 |
| *SEH1L* | Up | 0.458269308556538 | 0.0000206432742637582 |
| *SEMA3B* | Up | 0.39494589946014 | 0.00108179092733714 |
| *SEPT2* | Up | 1.04866634660233 | 5.66179439191029E-17 |
| *SEPT6* | Up | 0.859834188577559 | 1.70449264442474E-06 |
| *SEPT7* | Up | 0.676367729154471 | 1.75784888122798E-10 |
| *SERINC1* | Down | -0.740832653681341 | 0.000185692519500361 |
| *SERPINA1* | Up | 0.340048616730824 | 3.66043247939768E-07 |
| *SERTAD1* | Up | 0.291480347228386 | 0.0149057998812213 |
| *SETMAR* | Up | 0.726877620560447 | 2.44434961907847E-07 |
| *SF3A1* | Up | 0.677606528484675 | 8.70572100964439E-13 |
| *SF3A3* | Up | 0.831497199085624 | 2.23760683263234E-19 |
| *SF3B3* | Up | 0.744195882448192 | 2.25491637771506E-13 |
| *SFI1* | Up | 1.05205165808655 | 6.35147465341073E-24 |
| *SGOL1* | Up | 0.99370730749586 | 3.60825636882475E-29 |
| *SGOL2* | Up | 1.60621905372946 | 3.16068767175037E-14 |
| *SH3GL2* | Up | 0.29718710939679 | 2.67266084503922E-11 |
| *SHMT2* | Up | 0.402528822623376 | 0.000991340676546264 |
| *SIN3A* | Up | 0.432666238612184 | 0.000254922670830069 |
| *SIN3B* | Up | 1.4332507041518 | 3.2816970114815E-41 |
| *SKP2* | Up | 0.65813804911742 | 0.000039677511172963 |
| *SLC11A2* | Up | 0.590317598035052 | 4.76166309759849E-07 |
| *SLC12A4* | Up | 0.484638007062483 | 1.85850211648262E-06 |
| *SLC16A1* | Down | -0.686306178035764 | 0.00729071521652603 |
| *SLC19A2* | Up | 1.30972230688086 | 4.56426495088657E-16 |
| *SLC22A17* | Up | 1.20431889668521 | 4.94560076258649E-20 |
| *SLC22A5* | Up | 1.01938877241453 | 2.33175160565187E-15 |
| *SLC26A2* | Up | 0.657670735209209 | 5.79514003766538E-06 |
| *SLC27A2* | Down | -0.866185269916938 | 0.00825372729813412 |
| *SLC27A5* | Down | -0.970822996613245 | 0.00154848015351561 |
| *SLC2A8* | Up | 0.615446132260197 | 1.40505957732915E-14 |
| *SLC35B2* | Up | 0.958001442235051 | 6.9811596047525E-16 |
| *SLC35D1* | Down | -0.350761563273389 | 0.0429769774082148 |
| *SLC6A6* | Up | 0.946583453400031 | 0.0000117587709664505 |
| *SLCO1A2* | Up | 0.390819540995748 | 0.0146285117625598 |
| *SMAD5* | Up | 0.838301175224284 | 3.78630408823857E-09 |
| *SMARCC1* | Up | 0.718242192010507 | 7.02280121582815E-10 |
| *SMC1A* | Up | 0.820023062276562 | 1.09314108236719E-11 |
| *SMC1B* | Up | 0.683278343224421 | 1.37826131576327E-23 |
| *SMC3* | Up | 0.46421189120422 | 0.00118027557632002 |
| *SMC4* | Up | 1.04342691463225 | 2.562744066085E-11 |
| *SMC6* | Up | 0.80983337923953 | 3.18565320694138E-10 |
| *SMS* | Up | 0.586494607865471 | 1.71069427115778E-06 |
| *SNAI1* | Up | 0.56201666878547 | 1.30301621143742E-11 |
| *SNAP23* | Up | 0.281266281373998 | 0.00891315003015558 |
| *SNRPA* | Up | 1.02883152567415 | 9.59538493393013E-27 |
| *SNRPA1* | Up | 0.802169278485977 | 4.65453387262615E-13 |
| *SNRPB* | Up | 0.565070233054708 | 0.000003818322275993 |
| *SNRPB2* | Up | 0.344285699671016 | 0.0000374180898346755 |
| *SNRPD2* | Up | 0.464539643042249 | 0.0000014989049304097 |
| *SNRPF* | Up | 0.459338090691408 | 0.0000131285357444571 |
| *SNRPG* | Up | 0.645335397978247 | 4.34563690217344E-12 |
| *SNX2* | Down | -0.582233936184997 | 0.000605210839426118 |
| *SOCS1* | Up | 0.505442638330529 | 0.000180234302639304 |
| *SOCS3* | Up | 1.03395404609452 | 0.000150166476535038 |
| *SOS1* | Up | 1.02249220451321 | 6.22777611799196E-15 |
| *SOX13* | Up | 1.25364055773131 | 1.66567588390275E-29 |
| *SOX3* | Up | 0.774365612649322 | 1.2609198309418E-09 |
| *SOX4* | Up | 1.72507027950094 | 1.26039724875534E-07 |
| *SOX9* | Up | 1.90711294971086 | 9.6146515683807E-07 |
| *SPAG5* | Down | -0.395600498846528 | 0.00920407123129008 |
| *SRC* | Up | 1.00428530476565 | 5.9452638971765E-15 |
| *SRD5A1* | Down | -0.579979310266809 | 0.0128682448413044 |
| *SRM* | Up | 0.644753515475839 | 9.73556730425228E-07 |
| *SRPK1* | Up | 0.574051079422521 | 2.10009079245298E-07 |
| *SSB* | Up | 0.459043215527069 | 0.0000776442896536769 |
| *SSBP1* | Up | 0.510290807949554 | 2.35532222174565E-06 |
| *SSR3* | Up | 0.44203601467431 | 3.29435662150958E-06 |
| *SSRP1* | Up | 0.886575446782236 | 9.91215626098231E-22 |
| *STARD7* | Up | 0.355416054271913 | 4.40644053276679E-06 |
| *STAT4* | Up | 1.40273729699464 | 1.03708984233671E-16 |
| *STC2* | Up | 0.393045065851293 | 0.000925462920575896 |
| *STIL* | Up | 0.829176259804307 | 8.05190331698829E-09 |
| *STON1* | Up | 1.41214953911464 | 6.24134692936394E-12 |
| *STX12* | Up | 0.530611875958832 | 0.0000149695566865429 |
| *STX16* | Up | 0.855228523935756 | 4.92516731534737E-14 |
| *STX3* | Up | 1.11973431052787 | 1.198603100767E-13 |
| *STX7* | Up | 1.1320093701517 | 4.93015452894228E-16 |
| *SULT1A1* | Up | 0.327107633302825 | 0.045646632934664 |
| *SULT1A3* | Up | 1.495531157163 | 1.58682991957475E-21 |
| *SULT1A4* | Up | 1.495531157163 | 1.58682991957475E-21 |
| *SULT2B1* | Up | 1.07517622364462 | 9.47108631291647E-34 |
| *SUPT3H* | Up | 0.788718241096221 | 2.80070507115229E-12 |
| *SUPT5H* | Up | 0.779823246415204 | 9.19120053718238E-16 |
| *SURF1* | Down | -0.348737514256483 | 0.000426925823831746 |
| *SUV420H1* | Up | 0.897302181138053 | 2.6360706026941E-10 |
| *SUZ12* | Up | 0.590812274015308 | 1.40932185700861E-09 |
| *SYCE2* | Up | 0.921832611908861 | 6.13385734263541E-20 |
| *SYCP1* | Up | 1.06216248129195 | 9.52415607797806E-37 |
| *SYCP2* | Up | 0.682874850245434 | 3.06716347332405E-11 |
| *SYCP3* | Up | 1.08106142459397 | 1.09853839494195E-16 |
| *SYN1* | Up | 0.707770399359837 | 4.83128587885857E-16 |
| *SYNCRIP* | Up | 0.782396442737289 | 6.84280506885541E-11 |
| *SYT12* | Up | 0.484223002903557 | 0.00612635369772705 |
| *TAF1* | Up | 0.879519807206849 | 2.44983511772556E-12 |
| *TAF10* | Up | 0.31904652029057 | 0.000395889028401519 |
| *TAF11* | Up | 0.501270183311779 | 1.5918294553925E-07 |
| *TAF5* | Up | 0.853885746385824 | 3.71330587141157E-12 |
| *TAF5L* | Up | 0.383711418317428 | 0.000914532176327025 |
| *TAF6* | Up | 0.371374834556812 | 0.000238608739051927 |
| *TAF7L* | Up | 0.353629582329591 | 0.000000363847954319 |
| *TAL1* | Up | 0.448043315927918 | 3.48358405664376E-08 |
| *TBL1XR1* | Up | 0.497342942190274 | 5.58305035131468E-08 |
| *TBP* | Up | 0.842292723014923 | 1.66917265738159E-18 |
| *TCEB3* | Up | 0.884972395695712 | 1.00017688622401E-16 |
| *TCF12* | Up | 0.816728289265813 | 3.94397186331798E-10 |
| *TCIRG1* | Up | 1.34933299755479 | 1.57021880775998E-17 |
| *TEAD2* | Up | 0.709264061830476 | 0.0000777032369459484 |
| *TEAD3* | Up | 0.606175187831263 | 1.43472818814158E-09 |
| *TEX12* | Up | 0.232296363647088 | 3.03538252421743E-07 |
| *TEX14* | Down | -0.195439561113188 | 0.0118941680655132 |
| *TFAP2C* | Down | -0.368932903465672 | 0.000865962275727589 |
| *TFCP2L1* | Up | 0.441785348969602 | 4.96953301999844E-06 |
| *TFF1* | Up | 0.391136169703216 | 0.00656359523950365 |
| *TFPI2* | Up | 1.73832289090747 | 2.69947134064862E-12 |
| *TFR2* | Down | -0.903768236252525 | 0.0000517523109456969 |
| *TFRC* | Up | 1.3596628535245 | 6.61715582371544E-16 |
| *TGFB2* | Up | 1.20945253600378 | 6.68383641454167E-09 |
| *THSD4* | Up | 0.649117626389133 | 0.0000147513604507032 |
| *TIPARP* | Up | 0.611967264356736 | 5.73918216118038E-06 |
| *TJP3* | Up | 0.35154567386732 | 0.0227212980942591 |
| *TK2* | Up | 0.368306286049474 | 0.00142280449560784 |
| *TLE1* | Up | 0.743345421852371 | 7.56751674568952E-09 |
| *TLE3* | Up | 0.476625951937641 | 0.0000272051311539765 |
| *TLE4* | Up | 0.889627140865086 | 2.00217940726678E-07 |
| *TLR3* | Down | -0.816392906151833 | 0.00063712677155843 |
| *TMEM176B* | Down | -0.671659335565026 | 0.000179528889061714 |
| *TNFRSF1A* | Up | 0.410634109905272 | 0.000361990438217807 |
| *TNP2* | Up | 0.268540396943401 | 0.000296801148173181 |
| *TNRC6B* | Up | 1.7226620248186 | 1.37800023573606E-30 |
| *TOP3B* | Up | 1.43116925378275 | 1.83766826664354E-38 |
| *TOPBP1* | Up | 0.772042892999346 | 3.05307617740157E-12 |
| *TP53* | Up | 1.30814799020571 | 2.75346200263378E-15 |
| *TP53BP1* | Up | 1.03971717386714 | 1.00536791574948E-25 |
| *TP53INP2* | Up | 0.616551751788469 | 0.0000111913926038303 |
| *TPBG* | Up | 0.955605425270949 | 0.0000192616194069742 |
| *TPMT* | Down | -0.790553336983668 | 0.0000812393017533221 |
| *TPR* | Up | 0.623966300652073 | 0.0000105995806897041 |
| *TPSAB1* | Up | 0.66237628365993 | 4.57804609218372E-07 |
| *TPST1* | Up | 1.1494426735681 | 6.44724414595616E-15 |
| *TRAF2* | Up | 1.15938272295267 | 2.05781213877758E-31 |
| *TRAF3* | Up | 0.919463620955307 | 4.48083953344301E-12 |
| *TRAF5* | Up | 1.68246381261652 | 3.12418057081098E-16 |
| *TREH* | Up | 0.91869967154765 | 3.79832388888724E-12 |
| *TRIM28* | Up | 1.13624083348028 | 1.36065845100733E-17 |
| *TRIM29* | Up | 0.229920222648604 | 0.0333703603031907 |
| *TRIM37* | Up | 0.786227119276138 | 6.54410578295208E-13 |
| *TROVE2* | Up | 1.03717839475702 | 4.69527546524849E-20 |
| *TSC1* | Up | 1.09942061576804 | 2.05703764072056E-20 |
| *TSG101* | Up | 0.560707173005637 | 1.37002243021402E-11 |
| *TSSK2* | Down | -1.07297076163462 | 6.22447208972936E-24 |
| *TSTA3* | Up | 0.627997912493115 | 6.98289989447034E-06 |
| *TTPA* | Down | -2.49071946563731 | 5.05607887808158E-13 |
| *TUBB2B* | Up | 0.628364808050561 | 3.74615722805353E-06 |
| *TUBD1* | Up | 0.736944249760889 | 3.75785026205138E-12 |
| *TUBG1* | Up | 0.791263388148249 | 1.99894786765864E-08 |
| *TYR* | Up | 1.02777114792477 | 1.0262276665306E-27 |
| *UBE2D1* | Up | 0.51325195378983 | 0.000301626474098658 |
| *UBE2E1* | Up | 0.995889199338169 | 6.69203812765916E-18 |
| *UBE2L3* | Up | 0.696280634006587 | 2.00849118563766E-11 |
| *UBE2L6* | Up | 0.454931572252987 | 0.00226879266612035 |
| *UCK2* | Up | 0.32406904532485 | 0.0146261192559847 |
| *UCKL1* | Up | 0.839490636745067 | 4.96248494577197E-12 |
| *UGDH* | Down | -0.504794529824746 | 0.00517835929981664 |
| *UGP2* | Down | -0.712665101202516 | 0.0000850319186360576 |
| *UGT2A1* | Down | -0.968268607660393 | 5.70794706505799E-07 |
| *UGT2B15* | Down | -0.968395892090198 | 0.0124460631901219 |
| *UGT2B17* | Down | -3.12207293880601 | 4.07359638509718E-11 |
| *UGT2B4* | Down | -0.676797668959242 | 0.0214924875077333 |
| *UNC119* | Up | 1.12690726175082 | 4.5063830722498E-31 |
| *UNC13B* | Up | 0.718398177367992 | 5.60041543417332E-07 |
| *UPP1* | Up | 0.944731386073189 | 3.43310905831594E-11 |
| *UROS* | Up | 0.350821753586735 | 0.00103560663914113 |
| *USP11* | Up | 0.551760972653565 | 2.27317279260425E-07 |
| *USP22* | Up | 1.12139436101974 | 7.42238120333815E-16 |
| *USP27X* | Up | 0.574932931966483 | 2.44935157897018E-06 |
| *USP34* | Up | 2.11906970854109 | 2.24252193444545E-37 |
| *VAMP4* | Up | 0.981833809103025 | 6.55622965155348E-15 |
| *VDAC1* | Down | -0.410870028349329 | 0.00207250081231324 |
| *VDAC3* | Up | 1.05182890213605 | 6.61479520830337E-17 |
| *VPS28* | Up | 0.458224653408694 | 0.000748054740825999 |
| *VPS37B* | Up | 1.70423219531941 | 2.59422405162896E-27 |
| *VPS37D* | Up | 0.389261274865164 | 0.0000014871656234678 |
| *WDR34* | Up | 0.888956706215251 | 3.39341337922114E-11 |
| *WDR5* | Up | 1.27340485009983 | 1.30744526891245E-23 |
| *WDR75* | Up | 1.20898121886181 | 4.46369926338708E-23 |
| *WDR77* | Up | 0.75080295855866 | 3.26944222839435E-17 |
| *WFS1* | Up | 0.640479373098024 | 1.36251332919488E-07 |
| *WISP2* | Up | 1.02301035607849 | 2.46615737195211E-14 |
| *WNT6* | Up | 0.356950475365389 | 0.000522844155930782 |
| *XPC* | Up | 0.342703366038949 | 0.000274487068954512 |
| *XPO1* | Up | 0.712397930490848 | 1.7133305924886E-14 |
| *XRCC3* | Down | -0.254091250029479 | 0.00639672987093351 |
| *XRCC4* | Up | 0.519423625214627 | 0.000185888218070857 |
| *XRCC6* | Up | 0.480313522188943 | 3.82656308228301E-08 |
| *YAP1* | Up | 0.871594699829165 | 2.09544160393839E-08 |
| *YKT6* | Up | 0.841829377578791 | 2.18685919488538E-18 |
| *YWHAB* | Up | 0.664188874046321 | 6.68413189124727E-15 |
| *YWHAQ* | Up | 0.416412450161298 | 0.0000504105838173869 |
| *YWHAZ* | Up | 0.641182821764842 | 7.41828612728097E-06 |
| *YY1* | Up | 0.325742248457781 | 0.00018337486889544 |
| *ZBP1* | Up | 0.59035161422658 | 3.37142641427904E-12 |
| *ZFP36* | Up | 0.929798747625934 | 6.52727730063679E-07 |
| *ZFPM1* | Up | 1.18976521997476 | 1.4732383494381E-21 |
| *ZNF207* | Up | 0.708315118477346 | 4.74591474902602E-18 |
| *ZNRD1* | Up | 0.523357393291746 | 9.03895210311694E-08 |
| *ZW10* | Up | 0.519983078822214 | 6.169805173116E-09 |
| *ZWILCH* | Up | 0.53173255578735 | 0.0000089566701492067 |
